# Bimetallic Nanozyme Amplifier for Synergistic Ferroptosis‐Cuproptosis and Metabolic Reprogramming to Reshape Immunosuppressive Tumor Microenvironment

**DOI:** 10.1002/advs.202512764

**Published:** 2025-10-15

**Authors:** Jianzhang Luo, Kunzhao Huang, Xiaoyuan Yi, Pei Lu, Huaying Xie, Wen Li, Qingyu Zeng, Feifei He, Duo Wang, Liyan Wang

**Affiliations:** ^1^ Digestive Department The Affiliated Hospital of Guilin Medical University Lequn road No.15, Xiufeng district Guilin 541001 China; ^2^ Center of Interventional Radiology & Vascular Surgery Department of Radiology Zhongda Hospital Medical School Southeast University Nanjing 210009 China

**Keywords:** cuproptosis, ferroptosis, metabolic reprogramming, nanozyme, TME reprogramming

## Abstract

Although ferroptosis and cuproptosis have shown potential in tumor therapy, their anti‐tumor efficacy remains considerably limited due to aberrant metabolism within tumor cells and the immunosuppressive tumor microenvironment (TME). Herein, metal–organic framework nanoparticles (PEG@AuCZ@CC) are engineered by incorporating Au nanoparticles and α‐Cyano‐4‐hydroxycinnamic acid (CHCA), to maximize hydrogen peroxide production and induce metabolic shift from aerobic glycolysis to oxidative phosphorylation in tumor cells. Interestingly, metabolic reprogramming of tumor cells not only enhances both ferroptotic and cuproptotic signaling cascades, amplifies immunogenic cell death (ICD) effects, but also reduces intratumoral glucose consumption and extracellular lactate accumulation, thereby creating a glucose‐enriched but lactate‐depleted TME. Consequently, the platform significantly promotes dendritic cells maturation, enables epigenetic reprogramming of tumor‐associated macrophages (TAMs), and restores CD4⁺/CD8⁺ T‐cells functionality. This multimodal strategy reshapes the tumor microenvironment (TME) by integrating cell death modulation and cell metabolism regulation, effectively overcoming immune tolerance, presenting a promising paradigm for hepatocellular carcinoma (HCC) therapy.

## Graphical abstract

The Bimetallic Nanozyme Amplifier (PEG@AuCZ@CC) to maximize hydrogen peroxide production and reprogram tumor metabolism from glycolysis to oxidative phosphorylation. This shift enhances ferroptotic/cuproptotic signaling cascades and creates a glucose‐enriched but lactate‐depleted tumor microenvironment (TME), effectively overcoming immune tolerance in hepatocellular carcinoma therapy.

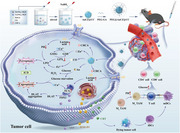

## 1 Introduction

Immunotherapy, which serves as a remedial therapeutic measure by leveraging or enhancing the patient's immune system, has garnered worldwide attention since its emergence.^[^ ^1^ ^]^ Unfortunately, hepatocellular carcinoma (HCC) is considered a “cold tumor” exhibiting limited sensitivity to immunotherapy.^[^ ^2^, ^3^ ^]^ With advances in the understanding of HCC progression and metastasis, the tumor immunosuppressive microenvironment (TME) has emerged as a crucial factor contributing to these undesirable outcomes.^[^ ^4^, ^5^, ^6^, ^7^ ^]^ It has been reported that the fluctuation in tumor‐associated macrophages (TAMs) is recognized as a hallmark of immunosuppression, e.g., pro‐tumorigenic M2 TAMs upregulation and antitumorigenic M1 TAMs decrease.^[^ ^8^, ^9^, ^10^ ^]^ Meanwhile, within the glucose‐deprived tumor microenvironment, CD4^+^/CD8^+^ T cells cannot maintain aerobic glycolysis for robust effector function.^[^ ^11^, ^12^ ^]^ In addition, exposure to the high concentration of lactate disturbs the metabolic activity of CD4^+^/CD8^+^ T cells, particularly dampening the nuclear factor of activated T cell expression and interferon‐gamma (IFN‐γ) production.^[^ ^13^, ^14^ ^]^ However, unlike CD4^+^/CD8^+^ T cells, immunosuppressive cells such as regulatory T (Treg) cells are highly adaptable to the glucose‐deprived and lactate‐enriched TME with stable immunosuppressive performance.^[^ ^15^, ^16^ ^]^ On this account, reprogramming glucose consumption and lactate concentration in the TME is beneficial for reshaping TAMs epigenetics to repolarize M2‐like TAMs into M1 ones. This metabolic intervention is also crucial for both restoring CD4^+^/CD8^+^ T cell function and triggering Treg cell destabilization, ultimately strengthening the antitumor immune performance of these immune cells.

Currently, there are many means that can be harnessed to reprogram the TME.^[^ ^17^ ^]^ Among them, reducing lactate levels in tumors is believed as the reliable and straightforward strategy. Lactate inhibition attenuates aerobic glycolysis, creating a glucose‐enriched but lactate‐depleted TME. This metabolic reprogramming facilitates the repolarization of M2 TAMs to M1 phenotypes. Concurrently, it may save CD4^+^/CD8^+^ T cells more nutritious food and establishes a favorable antitumor battlefield.

Certainly, only dependence on already existing CD4^+^/CD8^+^ T cells is insufficient to achieve satisfactory antitumor immunotherapy. Recent studies have revealed that immunogenic cell death (ICD) augments cancer cellular emission of damage‐associated molecular patterns (DAMPs), accelerates the rate of immune response, improves the tumor antigen presentation, recruits more CD4^+^/CD8^+^ T cells, and promotes CD4^+^/CD8^+^ T cells activation with subsequent intratumoral infiltration.^[^ ^18^, ^19^, ^20^ ^]^ However, ICD outcomes remain suboptimal due to the resistance of tumors to apoptosis and other mechanism.^[^ ^21^, ^22^, ^23^ ^]^ Given these challenges, there is an urgent need to synergistically induce multiple modes of cell death, while achieving high immunogenicity.^[^ ^24^, ^25^ ^]^

Here, we fabricate DSPE‐PEG2000‐GA(PEG‐GA)‐modified metal–organic framework nanomedicines, DSPE‐PEG2000‐GA@Au/Cu/ZIF‐8@CO&CHCA (denoted as PEG@AuCZ@CC). The α‐Cyano‐4‐hydroxycinnamic acid (CHCA) inhibits lactate transport, leading to the accumulation of lactate in tumor cells, which negatively feedback suppresses aerobic glycolysis, limits tumoral consumption of glucose, and establishes a M1 TAMs and CD4^+^/CD8^+^ T cells favorable TME with high glucose and low lactate levels, which however destabilizes Treg cells. Furthermore, caryophyllene oxide (CO) induces ferritinophagy, generating cytotoxic reactive oxygen species (ROS) and triggering ferroptosis.^[^ ^26^ ^]^ Cu^2+^ drives oxidative stress and mitochondrial dysfunction in tumor cells, ultimately inducing cuproptosis.^[^ ^27^, ^28^ ^]^ Thus, the synergistic effect of these two modes of cell death can activate ICD efficiently.^[^ ^29^ ^]^

Nanozymes are identified as one class of bioactive materials. Compared with natural enzymes, nanozymes circumvent the limitations of biological enzymes, such as poor biostability, severe systemic toxicity, high cost, and complex synthesis and purification processes, thereby exhibiting broad application prospects.^[^ ^30^ ^]^ In this report, Au nanozymes mimic glucose oxidase (GOx) to catalyze the oxidization of glucose in the presence of oxygen (O_2_), producing hydrogen peroxide (H_2_O_2_) and gluconic acid. This process can increase the concentration of H_2_O_2_ in tumors while depleting glucose supply of cancer cells, thereby intensifying ferroptosis and cuproptosis and eliciting robust ICD activation. Consequently, Ample DAMPs, such as adenosine triphosphate (ATP), calreticulin (CRT), and high mobility group box 1 protein (HMGB1), are released. These DAMPs triggering the maturation of dendritic cells (DCs), enabling efficient presentation of tumor antigens and secretion of anti‐inflammatory cytokines, ultimately eliciting a potent antitumor immune response.^[^ ^31^ ^]^

**Scheme 1:**
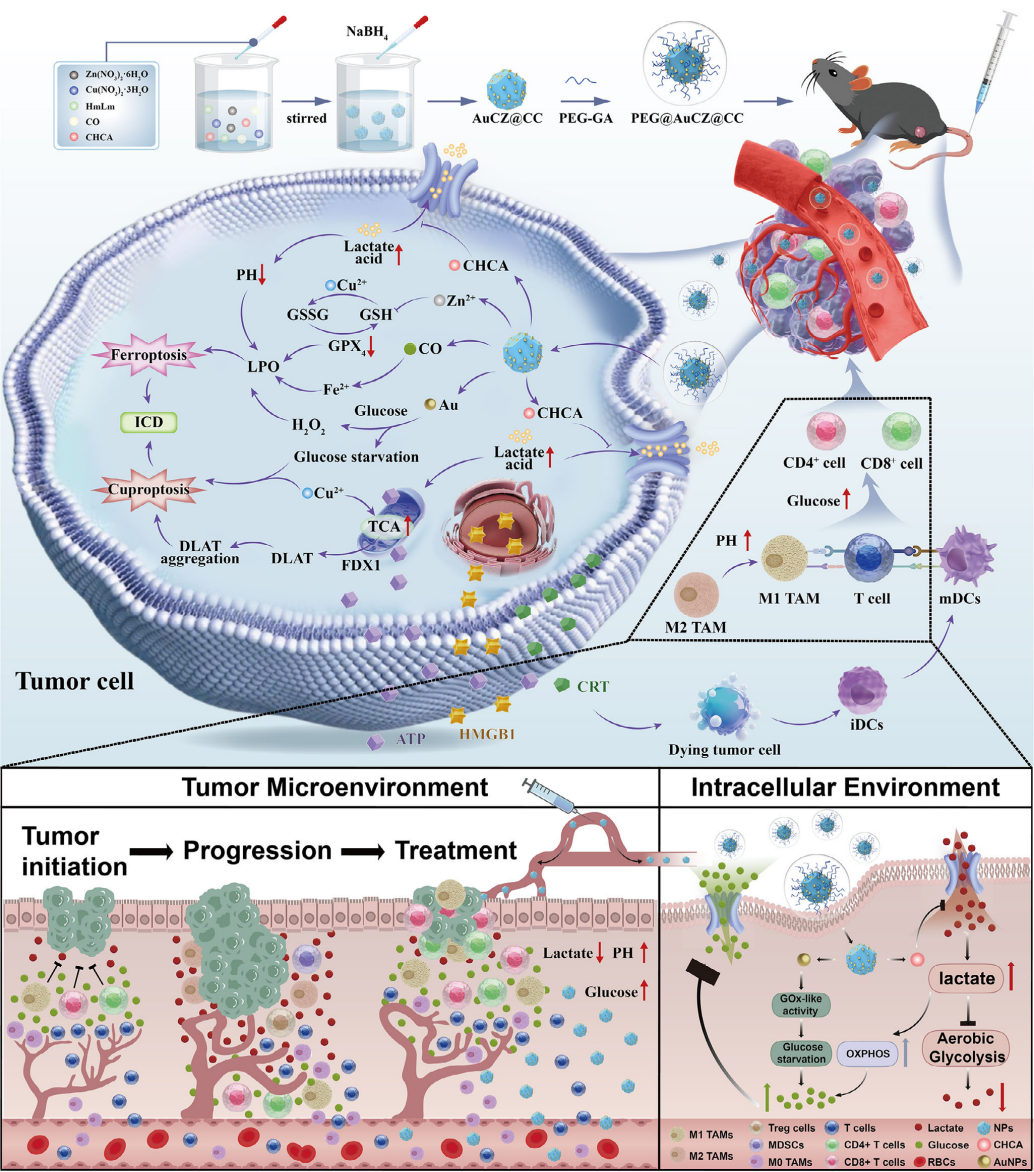
Synthesis procedures and design & action principles of PEG@AuCZ@CC unraveling how to synergistically combine ferroptosis and cuproptosis to enhance immunogenic cell death (ICD), while ameliorating the tumor immunosuppressive microenvironment, thereby collectively potentiating the overall diagram of antitumor immune responses.

Contributed by them, PEG@AuCZ@CC orchestrates bioenergetic and metabolic function to enhance the intensity of ICD triggered by ferroptosis and cuproptosis, remodels the TME, and suppress the activity of immunosuppressive cells (e.g., M2‐type macrophages, Treg cells, and myeloid‐derived suppressor cells). Concurrently, it promotes the activation and effector function of CD4^+^/CD8^+^ T cells, significantly boosting the infiltration and activation of effector T cells and memory T cells, thereby establishing a durable antitumor immune response and converting “cold tumors” into “hot tumors”. Consequently, systematic immune response activation and immunological surveillance recovery were anticipated, and tumor progression was tremendously blockaded via this approach (**Scheme** **1**).

Collectively, PEG@AuCZ@CC takes comprehensive and rational considerations into structural design and optimization to improve the biocompatibility and enhance immunotherapeutic efficacy, positioning it as a promising strategy to reverse immunosuppressive TME and treatment resistance or cancer plasticity.

## 2 Results and Discussion

### 2.1 Synthesis, Characterization of PEG@AuCZ@CC NPs

**Figure 1:**
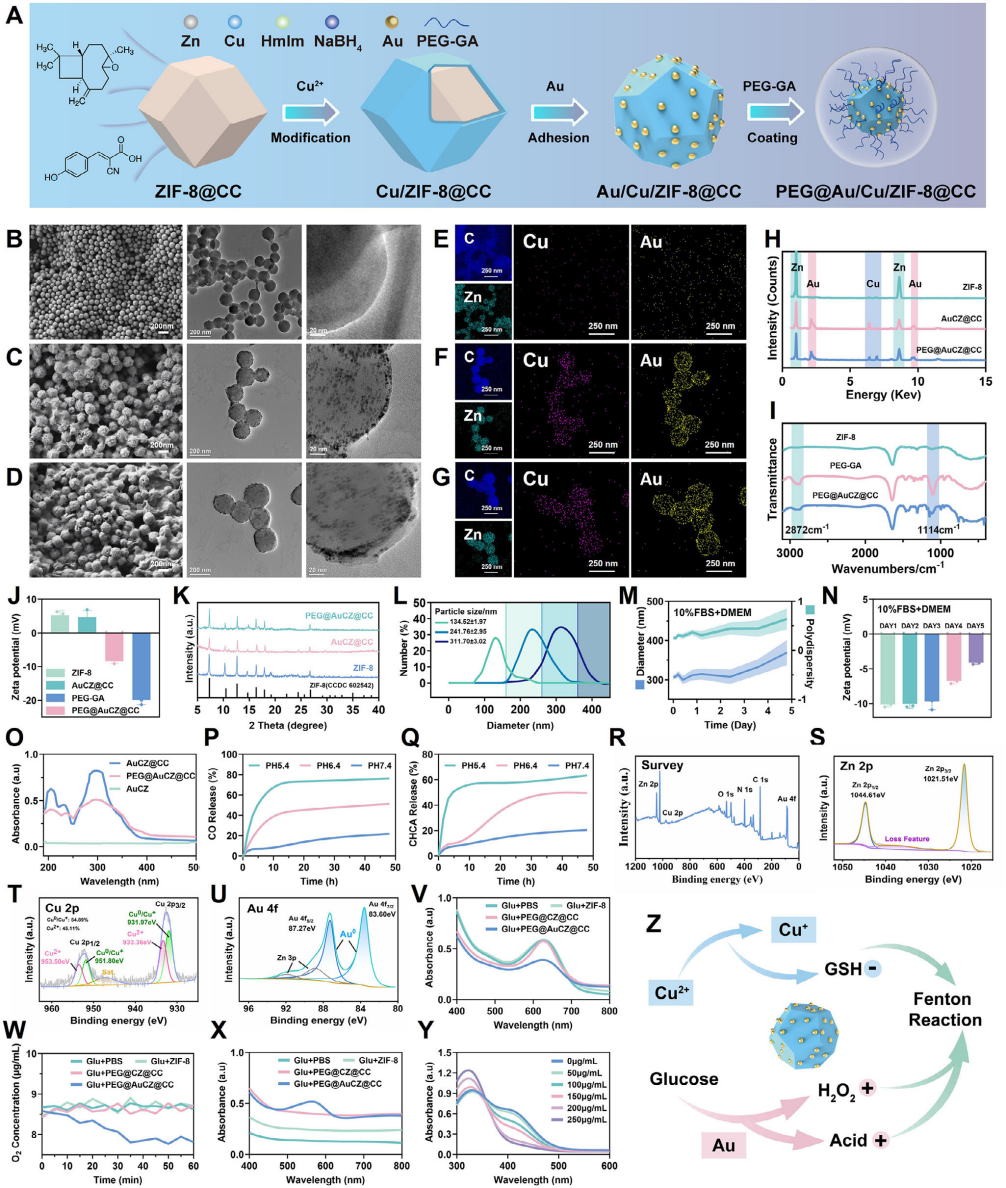
Characterization of the physicochemical properties of PEG@AuCZ@CC NPs. A) Schematic illustration depicting the preparation of PEG@AuCZ@CC. Representative SEM image and TEM image of B) ZIF‐8, C) AuCZ@CC, and D) PEG@AuCZ@CC. Scale bar = 200 nm. Inset: amplified nanomedicines (scale bar = 20 nm). Elemental mapping of E) ZIF‐8, F) AuCZ@CC, and G) PEG@AuCZ@CC. H) Energy diffraction spectrum (EDS) of nanomedicines. I) Fourier transform infrared spectra of the ZIF‐8, PEG‐GA, and PEG@AuCZ@CC NPs. J) Zeta potential of nanomedicines and DSPE‐PEG2000‐GA. (K) XRD patterns of ZIF‐8, AuCZ@CC, and PEG@AuCZ@CC NPs. L) Hydrated particle size of ZIF‐8, AuCZ@CC, and PEG@AuCZ@CC in PBS by Dynamic light scattering (DLS). The M) stability and N) zeta potential of PEG@AuCZ@CC in 10% FBS‐supplemented DMEM for 5 days. O) UV–vis absorption spectra of AuCZ, AuCZ@CC, and PEG@AuCZ@CC. Accumulative release profiles of P) CO and Q) CHCA from PEG@AuCZ@CC under different times and different pH. (R) PEG@AuCZ@CC NPs XPS survey spectrum and high‐resolution XPS spectra of S) Zn 2p, T) Cu 2p, and U) Au 4f. V) Test of glucose consumption by PEG@AuCZ@CC NPs in glucose solutions. W) Time‐dependent oxygen concentration curve during co‐incubation of glucose with ZIF‐8, PEG@CZ@CC, and PEG@AuCZ@CC. (X) H_2_O_2_‐generating ability under various treatments. Y) UV–vis spectra of DTNB in the presence of GSH and PEG@AuCZ@CC NPs under different concentrations. Z) Schematic of enzymes‐mimicking biocatalytic process. Data are expressed as mean ± standard deviation (SD, n=3).

ZIF‐8 was prepared following the hydrothermal synthesis protocol originally developed by Wiebcke's research.^[^ ^32^ ^]^ The AuCZ@CC was synthesized via a one‐pot method by co‐doping copper and the drugs CO/CHCA, followed by in situ reduction of HAuCl_4_ with NaBH_4_. Finally, we coated the AuCZ@CC with a layer of PEG‐GA to obtain PEG@AuCZ@CC (**Figure** **1**A). Scanning electron microscopy (SEM) images and Transmission electron microscopy (TEM) images revealed that the ZIF‐8 exhibited a smooth surface with a typical rhomboidal dodecahedron structure (Figure 1B). Compared to pristine ZIF‐8 particles, the AuCZ@CC NPs exhibited a distinct granular structure, with uniformly distributed Au nanoparticles (≈2–3 nm in size) on the surface (Figure 1C). The PEG@AuCZ@CC NPs was wrapped with a layer of PEG‐GA around the periphery, presenting translucent films covering the nanoparticles with spherical morphology and good dispersibility, while Au nanoparticles appear slightly blurred yet remain uniformly distributed (Figure 1D). Moreover, during the synthesis process, the solution color transitioned sequentially from milky white to ginger yellow, and finally to pale wine red, indicating the successful formation of the materials in each step (Figure S1, Supporting Information). Subsequently, elemental mapping was performed to visualize the distribution of constituent atoms in ZIF‐8, AuCZ@CC, and PEG@AuCZ@CC NPs. The ZIF‐8 is primarily composed of Zn and C atoms (Figure 1E). In the AuCZ@CC NPs, new atoms Cu and Au appeared, indicating the successful doping of Cu into the ZIF‐8 framework and effective decoration with Au nanoparticles (Figure 1F). Notably, PEG@AuCZ@CC NPs maintained these characteristics, demonstrating that the DSPE‐PEG2000‐GA coating preserved the structural integrity of AuCZ@CC NPs (Figure 1G). EDS inspection also validates the presence of Zn, Cu, and Au atoms (Figure 1H). The Fourier transform infrared spectra showed that PEG@AuCZ@CC NPs had characteristic peaks corresponding to DSPE‐PEG2000‐GA (2872 cm^−1^ for ─CH_2_ and 1114 cm^−1^ for C─O─C), confirming successful modification (Figure 1I). A subsequent decrease in zeta potential from +4.66 mV for AuCZ@CC NPs to −8.34 mV for PEG@AuCZ@CC NPs was observed, further confirming successful PEGylation (Figure 1J). Furthermore, we employed the x‐ray diffraction (XRD) to evaluate the phase structures of each synthesized products. The AuCZ@CC NPs displayed strong and weak peaks at 7.3°, 10.35°, 12.7°, 14.8°, 16.4°, 18.0°, and 26.7°, corresponding to the (110), (200), (211), (220), (310), (222), and (431) crystal planes of ZIF‐8, respectively^[^ ^33^ ^]^ (Figure 1K). This indicated that the loading of CO and CHCA, as well as the modification with Cu and Au, did not compromise the structural stability of AuCZ@CC. The material maintained the body‐centered cubic lattice and high crystallinity identical to ZIF‐8. And the PEG@AuCZ@CC NPs correspondingly presented the characteristic peaks of ZIF‐8 in XRD, even more distinct than AuCZ@CC. This indicates that the DSPE‐PEG2000‐GA coating not only does not compromise the crystalline structure of AuCZ@CC but may also contribute to its long‐term stabilization (Figure 1K).

Generally, nanoparticles with appropriate and stable particle sizes are more favorable for systemic pharmacokinetics and tumor‐specific deposition. Consequently, hydrodynamic diameters of ZIF‐8, AuCZ@CC, and PEG@AuCZ@CC NPs were characterized using dynamic light scattering (DLS). The average particle sizes of ZIF‐8, AuCZ@CC NPs, and PEG@AuCZ@CC NPs were measured to be 134.52 ± 1.67, 241.76 ± 2.95, and 311.70 ± 3.02 nm, respectively (Figure 1L). The hydrodynamic diameters measured by DLS for all three materials were consistently larger than those obtained from TEM analysis. This discrepancy arises because DLS measures the hydrated particles, including their surrounding water shells, whereas TEM characterizes dried individual nanoparticles. Notably, after dispersing the PEG@AuCZ@CC NPs in ultrapure water and 10% FBS‐supplemented DMEM, the nanoparticles maintained minimal fluctuations in both hydrodynamic size and Polydispersity Index (PDI) over 72 h, with no significant alterations in zeta potential (Figure 1M,N). Considering that the ultimate goal of this research is clinical translation, it is necessary to conduct more multidimensional stability tests in a serum‐based application environment. Therefore, we simulated a serum environment and performed a multidimensional stability assessment over 5 days. The results demonstrate the excellent stability of PEG@AuCZ@CC NPs under both storage and physiological conditions (Figure S2, Supporting Information).

Subsequently, we employed UV–vis spectroscopy to validate the successful loading of the CO and CHCA. Characteristic absorption peaks were observed at 208 nm for CO and 324 nm for CHCA, whereas the AuCZ NPs exhibited no significant UV absorption in the 200–800 nm wavelength range (Figure S3, Supporting Information). Due to the loading of CO and CHCA, both AuCZ@CC NPs and PEG@AuCZ@CC NPs exhibited characteristic absorption peaks at ≈200 and 300 nm (Figure 1O). Compared with the spectra of CO and CHCA, the positions of the peaks were slightly blue‐shifted, indicating that the drugs were successfully loaded into the nanomaterials. Furthermore, the characteristic peaks of PEG@AuCZ@CC NPs were attenuated compared to AuCZ@CC NPs, which can be attributed to the physical shielding effect of PEG‐GA. After confirming the successful loading of the drug, we measured the UV absorbance of CO and CHCA at various concentrations to establish standard calibration curves, both demonstrating excellent linearity (R^2^ > 0.99) (Figure S4, Supporting Information). Analysis of the supernatant, collected during nanoparticle preparation, we found that the content of free CO and CHCA were significantly lower than the initial input quantities, further verifying partial drug encapsulation within the nanoparticles (Figure S5, Supporting Information). We also calculated that the drug loading rate of 4.7% for CO and 6.6% for CHCA, with encapsulation rate was 51% and 70%, respectively. Then, the PEG@AuCZ@CC nanocomposites were loaded into a 3.5 kDa molecular weight cutoff dialysis bag and immersed in PBS with different pH (pH 7.4 simulating physiological environment, pH 6.4 simulating tumor microenvironment, and pH 5.4 simulating endosomal/lysosomal environment) under gentle shaking (100 rpm) at 37 °C (simulating the physiological temperature and the fluidity of body fluids). First, we evaluated the stability of PEG@AuCZ@CC NPs in solutions with different pH values by monitoring the release of Zn^2+^ and Cu^2+^ ions (Figure S6, Supporting Information). It can be seen that the infrastructure of PEG@AuCZ@CC NPs rapidly collapses under acidic environment, while the nanostructure remained stable for extended periods at neutral environment. These pH‐responsive characteristics enabled on‐demand drug release in acidic environments and sustained drug release under physiological conditions, thereby enhancing tumor cell killing efficacy while minimizing toxicity to normal tissues (Figure 1P,Q).

### 2.2 Enzyme‐Like Activities of PEG@AuCZ@CC NPs

Prior to investigating the enzyme‐like activity of the PEG@AuCZ@CC NPs, we used X‐ray photoelectron spectroscopy (XPS) to analyze the valence states and ratios of the metallic components Zn, Cu, and Au (Figure 1R–U). The Zn 2p spectrum exhibited two distinct peaks corresponding to the spin‐orbit coupling components of Zn 2p3/2 and Zn 2p1/2, with an energy difference of 23.1 eV between the two peak positions, indicating the presence of Zn^2+[^ ^34^ ^]^ (Figure 1S). In the Cu 2p spectrum, peaks at 931.97 and 951.80 eV were attributed to Cu^0/+^, while peaks at 933.36 and 953.50 eV were ascribed to Cu^2+^ (Figure 1T). This not only confirmed the coexistence of Cu^+^ and Cu^2+^ in PEG@AuCZ@CC but also demonstrated its potential to deplete glutathione (GSH) and directly induce cuproptosis. In the Au 4f spectrum, peaks at 83.60 and 87.27 eV were assigned to Au 4f7/2 and Au 4f5/2, respectively, indicating that the Au in the sample was in a zero‐valent state, which is beneficial for Au nanoparticles to exert enzyme‐like activities (Figure 1U). Additionally, a slight negative shift was observed in the Au 4f7/2 peak (83.60 eV) compared to the reference binding energy of Au 4f7/2 (84.00 eV). This shift is related to the interaction with ZIF‐8, indicating the successful encapsulation or impregnation of Au nanoparticles onto the surface of the ZIF‐8 material.^[^ ^35^ ^]^

The gold nanoparticles (AuNPs) exhibit glucose oxidase (GOx)‐like activity, mediating the oxidation of glucose, generating gluconolactone (hydrolyzed to gluconic acid) and hydrogen peroxide (H_2_O_2_) as byproducts. This process not only provides an acidic microenvironment but also generates essential reactants for Fenton reaction. To evaluate the GOx‐like activity of AuNPs, PBS, ZIF‐8, PEG@CZ@CC, and PEG@AuCZ@CC were separately mixed with glucose solution under mineral oil overlay (to prevent oxygen exchange). The enzymatic performance was systematically assessed through four parameters: i) glucose consumption, ii) dissolved oxygen depletion, iii) pH variation, and iv) H_2_O_2_ generation in the reaction system. First, the glucose concentration was quantified using the O‐toluidine method based on its chromogenic reaction with glucose. UV–vis spectra revealed strong absorption peaks at 630 nm for both Glucose + PBS, Glucose + ZIF‐8, and Glucose + PEG@CZ@CC groups, whereas the Glucose + PEG@AuCZ@CC group exhibited significantly attenuated peak intensity at the same wavelength (Figure 1V). Quantitative analysis demonstrated that ≈40% glucose consumption by PEG@AuCZ@CC NPs in the solution (Figure S7, Supporting Information). Concurrently, we monitored the dissolved oxygen (DO) for 1 hour, which revealed a significant decrease in DO levels in the Glucose + PEG@AuCZ@CC group, demonstrating oxygen consumption during the enzymatic reaction (Figure 1W). Similarly, the observed decrease in solution pH indicated the formation of acidic byproducts (Figure S8, Supporting Information). Subsequently, we detected substantial hydrogen peroxide (H_2_O_2_) generation in both the Glucose + ZIF‐8 group, Glucose + PEG@CZ@CC group and Glucose + PEG@AuCZ@CC group by ultraviolet spectrophotometer (Figure S9, Supporting Information). However, subsequent UV‐vis spectral measurements revealed that the absorbance increase in the Glucose + ZIF‐8 group and Glucose + PEG@CZ@CC group originated from intrinsic electronic transitions of the nanomaterials, whereas the Glucose + PEG@AuCZ@CC group exhibited a characteristic absorption peak at 560 nm, which unequivocally confirmed H_2_O_2_ generation (Figure 1X).

Reduced glutathione (GSH) serves as a pivotal component in cellular antioxidant systems. The Cu^2+^ ions in nanomaterials can be reduced to Cu^+^ ions by GSH, thereby depleting the overexpressed GSH in tumor cells. The ability of nanomaterials to deplete GSH was quantified using DTNB as a colorimetric probe. Compared with the strong absorption peaks at 412 nm observed in the GSH group, the addition of AuCZ@CC NPs and PEG@AuCZ@CC NPs to GSH solutions resulted in significant attenuation or even complete disappearance of the 412 nm peak (Figure S10, Supporting Information). This unequivocally demonstrates that Cu^2+^ ions doping in the nanomaterials enables efficient GSH depletion. In addition, we observed that the ability of PEG@AuCZ@CC NPs to consume GSH was positively correlated with its concentration (Figure 1Y). Finally, the concentration of GSH under different Nanomedicines and different concentrations of PEG@AuCZ@CC NPs were consistent with UV–vis spectrophotometric data (Figure S11A,B, Supporting Information). The observable color transition provided direct visual confirmation of these findings (Figure S11C,D, Supporting Information). In summary, the PEG@AuCZ@CC nanomaterial demonstrates excellent GOX‐like activity and the ability to deplete GSH, which is conducive to enhancing the intensity of the Fenton reaction (Figure 1Z).

### 2.3 Cell Uptake and Anti‐Tumor Mechanism of PEG@AuCZ@CC NPs

**Figure 2:**
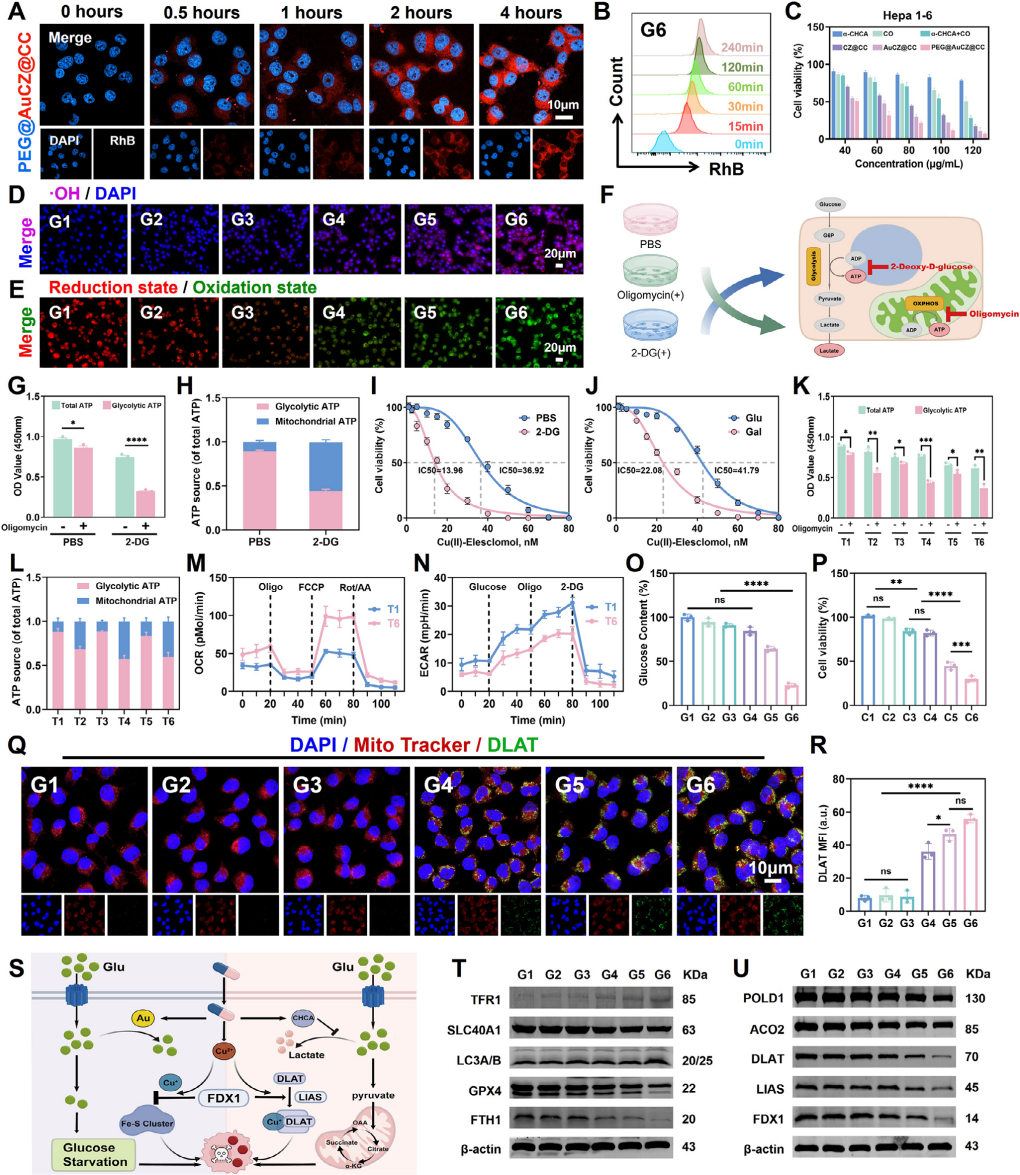
In vitro cellular uptake of PEG@AuCZ@CC NPs and anti‐tumor mechanism study. A) CLSM images of Hepa1‐6 cells following incubation with Rhodamine B‐labeled PEG@AuCZ@CC NPs for different durations, with cell nuclei stained using DAPI (Scale bar: 10 µm). B) Flow cytometry analysis of cellular uptake efficiency of Rhodamine‐B labeled PEG@AuCZ@CC NPs. C) Cytotoxicity assay of Hepa1‐6 cells after different treatments for 24 h, *n* = 6. D) Fluorescence inverted microscopy images of Hepa1‐6 cells stained with hydroxyl radicals after different treatments, wherein cell nuclei are stained with DAPI. (Scale bar: 20 µm). E) Fluorescence inverted microscopy images of Hepa1‐6 cells stained with C11‐BODIPY fluorescent probe after different treatments. (Scale bar: 20 µm). F) Schematic of metabolites altered after Oligomycin or 2‐deoxy‐D‐glucose treatment of Hepa1‐6 cells. G) The ATP source of Hepa1‐6 cells after different treatments. H) The main metabolic pathway of Hepa1‐6 cells shifted from glycolysis to oxidative phosphorylation. I) Viability of Hepa1‐6 cells grown in medium containing either PBS or 2‐DG treated with Cu(II)‐Elesclomol. J) Viability of Hepa1‐6 cells grown in medium containing either high‐glucose or galactose, treated with Cu(II)‐Elesclomol. K)The ATP source of Hepa1‐6 cells after different treatments. L)The main metabolic pathway of Hepa1‐6 cells shifted from glycolysis to oxidative phosphorylation after different treatments. Seahorse assay measuring the M) OCR and N) ECAR of Hepa1‐6 cells repeat treated with PEG@Au/Cu/ZIF‐8@CHCA. O) The glucose content in Hepa1‐6 cells with various treatments. P) Viability of Hepa1‐6 cells grown in different medium. Q) Representative CLSM images and R) Semi‐quantitative analysis of DLAT (green) expression in Hepa1‐6 cells with various treatments. Cell mitochondria were co‐stained with MitoTraker (red), and nuclei were co‐stained with Hoechst 33 342 (blue). (Scale bar: 10 µm). S) Schematic diagram of intracellular cuproptosis induced by PEG@AuCZ@CC NPs. T) Western blotting analysis of TFR1, SLC40A1, LC3A/B, GPX4, and FTH1 in Hepa1‐6 cells after different treatments. U) Western blotting analysis of POLD1, ACO2, DLAT, LIAS, and FDX1 in Hepa1‐6 cells after different treatments. Data are expressed as mean ± SD (*n* = 3). One‐way ANOVA or t‐test was used to analyze statistical differences between groups. ^*^ *p* <0.05, ^**^ *p* <0.01, ^***^ *p* <0.001, ^****^ *p* <0.0001. "ns" denotes no significant difference. Note, G1: Control, G2: CO, G3: CO&CHCA, G4: CZ@CC, G5: AuCZ@CC, G6: PEG@AuCZ@CC NPs.

Prior to investigating the antitumor mechanisms in vitro, it was essential to verify the cellular uptake of PEG@AuCZ@CC by Hepa1‐6 cells. Using rhodamine B‐labeled AuCZ@CC NPs and PEG@AuCZ@CC NPs, we systematically evaluated their time‐dependent cellular internalization. As can be seen, both nanoparticle groups exhibited time‐dependent enhancement of intracellular red fluorescence intensity with prolonged incubation (**Figure** **2**A; Figure S12A, Supporting Information). Importantly, the flow cytometry offers greater objectivity and sensitivity compared to fluorescence imaging. Therefore, additional time points (15 min group) were added to the flow cytometry analysis to better capture the continuous dynamic process (Figure 2B; Figure S12B, Supporting Information). Given the presence of zinc ions and copper ions in the carrier composition, we used zinc‐specific fluorescent probe (Zinpyr‐1) and Copper (Cu) Assay Kit to analyze the zinc and copper ion content in cells from different treatment groups (Figures S13 and S14, Supporting Information). Concurrently, β‐caryophyllene oxide (CO) induces ferritinophagy, so we analyzed the intracellular total iron and Fe^2+^ levels in different treatment groups (Figures S15 and S16, Supporting Information). Collectively, these results demonstrate that the treatment of PEG@AuCZ@CC NPs leads to a marked elevation of intracellular concentration of Zn^2+^, Cu^2+^, and Fe^2+^. These findings confirm successful cellular internalization of the PEG@AuCZ@CC NPs followed by controlled degradation to release both carrier components and therapeutic payloads. Prior to evaluating the cytotoxicity of various Nanomedicines, we assessed the biosafety of PEG@AuCZ@CC NPs in normal hepatocytes (AML‐12 cells and THLE‐2 cells). It can be seen that more than 60% of normal hepatocytes survived even at 120 µg mL^−1^ PEG@AuCZ@CC NPs. And due to the little GA expression on normal hepatocytes, PEG@AuCZ@CC NPs behaved more safely than AuCZ@CC NPs (Figure S17A,B, Supporting Information). In contrast, dose‐dependent cytotoxicity was observed in Hepa1‐6 cells with each Nanomedicines (Figure 2C). Among them, PEG@AuCZ@CC NPs showed the strongest killing effect with an IC50 of only 33 µg mL^−1^ (Figure S18, Supporting Information).

As established in preceding studies, we know that PEG@AuCZ@CC NPs significantly elevate intracellular Fe^2+^ and Cu^2+^ levels in Hepa1‐6 cells. Such dysregulation of transition metal homeostasis potently induces concurrent ferroptosis and cuproptosis. Therefore, we conducted cell rescue experiments using iron chelator (DFO), copper chelators (TTM and TEPA), necrosis inhibitor, and pyroptosis inhibitor. Compared to PEG@AuCZ@CC nanoparticles alone, the groups treated with PEG@AuCZ@CC nanoparticles in combination with either an iron chelator or a copper chelator showed an increase in cell survival rate. And the group treated with a combination of the iron chelator and copper chelator exhibited the highest cell survival rate. Additionally, neither the necrosis inhibitor nor the pyroptosis inhibitor improved cell viability (Figure S19, Supporting Information). This suggests that the PEG@AuCZ@CC NPs induce tumor cell death via iron/copper‐dependent pathways.

Ferroptosis damages cells via Fenton reaction, which generates oxidative stress.^[^ ^36^ ^]^ However, due to the neutral‐to‐alkaline intracellular environment and insufficient H_2_O_2_ levels in tumor cells, the intensity of the Fenton reaction is severely limited. Inspired by the Warburg effect, which states that tumor cells tend to favor glycolysis to produce abundant lactate even under aerobic conditions. We loaded CHCA, a non‐competitive monocarboxylate transporter inhibitor targeting MCT1/2/4, into the nanocarrier to block lactate efflux, thereby effectively increasing intracellular acidity. Furthermore, AuNPs were incorporated to mimic glucose oxidase (GOx) activity, generating gluconic acid and substantial H_2_O_2_. We anticipate that these measures will enhance the intensity of the Fenton reaction.

First, it is necessary to determine whether Hepa1‐6 cells exhibit the Warburg effect. We cultured Hepa1‐6 cells in either standard medium and cobalt chloride (CoCl_2_)‐supplemented medium for 48 h, respectively. Afterwards, we performed Western blot analysis to detect the hypoxia marker HIF‐1α. The results showed that Hepa1‐6 cells cultured in CoCl_2_‐supplemented medium established hypoxic conditions (Figure S20, Supporting Information). Concurrently, the lactate content in both cellular lysates and culture mediums was measured separately, we found that the final lactate content of Hepa1‐6 cells was basically the same in both normoxic and hypoxic conditions (Figure S21, Supporting Information). This proves that Hepa1‐6 cells exhibit the Warburg effect.

Subsequently, we compared the intracellular lactate content of Hepa1‐6 cells in different nanomedicines groups. The results showed that CHCA treatment significantly increased intracellular lactate accumulation (Figure S22, Supporting Information). The pH fluorescent probe also showed that intracellular pH drops or acidity rise (Figure S23, Supporting Information). At the same time, we examined the intracellular hydrogen peroxide content. Notably, AuNPs significantly enhanced H_2_O_2_ production in Hepa1‐6 cells (Figure S24, Supporting Information). On this basis, both AuCZ@CC NPs and PEG@AuCZ@CC NPs provoked a pronounced accumulation of reactive oxygen species (ROS) within cellular compared to other groups (Figures S25 and S26, Supporting Information). Given that ROS includes a variety of substances such as peroxides, superoxides, hydroxyl radicals (·OH), singlet oxygen, and α‐oxygen. we directly quantified ·OH as the terminal product of Fenton reaction. Consistent with the results of ROS detection, the most significant fluorescence signals were observed in the AuCZ@CC NPs and PEG@AuCZ@CC NPs groups (Figure 2D; Figure S27, Supporting Information). These results demonstrate that coordinated metabolic modulation of lactate and glucose by CHCA and AuNPs effectively acidified the intracellular environment while elevating H_2_O_2_ content, thereby significantly potentiating Fenton reaction efficiency.

However, the Fenton reaction efficacy is attenuated by tumor cells' sophisticated antioxidant systems, including GSH, GPX4, Nrf2, HMOX1, NQO1, and so on.^[^ ^37^, ^38^, ^39^, ^40^ ^]^ In the previous section, we found that PEG@AuCZ@CC NPs released substantial zinc ions in Hepa1‐6 cells. Zinc overload can reduce the cystine intake by cells, and consequently impairs GSH biosynthesis.^[^ ^41^, ^42^ ^]^ Concurrently, Cu^2+^ released from the nanocarriers will consume the existing GSH directly. Therefore, in the nanomedicine groups, GSH levels in Hepa1‐6 cells exhibited a significant decrease (Figures S28 and S29, Supporting Information). Through Western blot analysis, we can see that the expression levels of Nrf2, HMOX1, and NQO1 in cells were downregulated because β‐caryophyllene oxide (CO) directly inhibits multiple cellular redox regulators^[^ ^26^ ^]^ (Figure S30, Supporting Information). Under these conditions, both total antioxidant capacity (TAC) and DPPH radical scavenging activity in the cells were significantly weakened (Figure S31A,B, Supporting Information). Through potentiation of Fenton reaction and depletion of cellular reducing capacity, the accumulated ·OH will subsequently converted into lipid hydroperoxides via coordinated catalysis by acyl‐CoA synthetase long chain family 4 (ACSL4), lysophosphatidylcholine acyltransferase 3 (LPCAT3), and lipoxygenases (LOXs).^[^ ^43^, ^44^ ^]^ Therefore, we evaluated the progression of lipid peroxidation (LPO) in Hepa1‐6 cells using BODIPY 581/591 C11 fluorescent probe and malondialdehyde (MDA) assay kits (Figure 2E; Figures S32 and S33, Supporting Information). The results demonstrated that PEG@AuCZ@CC NPs significantly aggravates the LPO process in cells. Excessive lipid hydroperoxides induce catastrophic damage to biomembranes, amino acids, and nucleic acids, ultimately culminating in ferroptosis.

The critical step in cuproptosis involves oligomerization of dihydrolipoamide S‐acetyltransferase (DLAT), which triggers proteotoxic stress. Ferredoxin 1 (FDX1), as the upstream regulator of lipoamide acetylation, modulates acetylation of multiple proteins, including DLAT. Concurrently, FDX1 reduces Cu^2+^ to the more toxic Cu^+^, inhibits the synthesis of iron‐sulfur cluster proteins in the respiratory chain complex, induces Fe‐S cluster deficiency, thereby eliciting proteotoxic stress that ultimately culminates in cell death.^[^ ^45^, ^46^, ^47^ ^]^ This process is intimately linked to the tricarboxylic acid (TCA) cycle. Existing studies demonstrate that the main energy metabolism in cells will be transferred to the oxidative phosphorylation pathway of mitochondria after inhibiting glycolysis, that is, the TCA cycle is enhanced.^[^ ^48^, ^49^, ^50^ ^]^ To investigate whether potentiation of oxidative phosphorylation augments cuproptosis susceptibility, we conducted comparative analyses of ATP production from glycolysis versus oxidative phosphorylation across treatment groups. This experimental design enables determination of dominant metabolic pathways and their correlation with cuproptosis sensitivity (Figure 2F). In the control group, we measured the content of total ATP in the cells, then used Oligomycin to inhibit the oxidative phosphorylation pathway. The observed marginal ATP decrease represented oxidative phosphorylation‐derived ATP, while the residual ATP was glycolytically generated. This indicates that Hepa1‐6 cells predominantly rely on glycolysis for energy production under basal conditions. In the 2‐deoxy‐D‐glucose (2‐DG, glycolysis inhibitor) treatment group, the ATP produced by the cells decreased significantly, indicating that the oxidative phosphorylation as the dominant ATP source (Figure 2G). By comparing the ATP generated by different pathways, it can be seen that the main metabolic pathway of Hepa1‐6 cells shifted from glycolysis to oxidative phosphorylation (Figure 2H). We then treated cells with different concentrations of Cu(II)‐Elesclomol and measured changes in cell viability. Measurement results confirmed that the viability of Hepa1‐6 cells in the 2‐DG‐treated group was significantly reduced (Figure 2I). To exclude the potential toxicity of Oligomycin and 2‐DG, we cultured Hepa1‐6 cells in high‐glucose medium (promoting glycolysis) and galactose medium (inhibiting glycolysis),^[^ ^48^ ^]^ respectively, and followed by identical Cu(II)‐Elesclomol treatments. Similar to the previous results, Hepa1‐6 cells in galactose medium showed a significant decrease in viability compared to high glucose medium (Figure 2J). Notably, our findings demonstrated that the predominant energy metabolism in Hepa1‐6 cells is shifted to the oxidative phosphorylation pathway, thereby enhancing the tricarboxylic acid (TCA) cycle activity, which makes cells more sensitive to Cu(II)‐Elesclomol.

Using an identical analytical approach, we evaluated six treatment groups: Cu/ZIF‐8 (T1), Cu/ZIF‐8@CHCA (T2), Au/Cu/ZIF‐8 (T3), Au/Cu/ZIF‐8@CHCA (T4), PEG@Au/Cu/ZIF‐8 (T5), and PEG@Au/Cu/ZIF‐8@CHCA (T6). It can be seen that CHCA successfully inhibited glycolysis in Hepa1‐6 cells, and its energy metabolism was shifted to oxidative phosphorylation to different degrees (Figure 2K,L). Considering the tumor cells are metabolically plastic and can switch back to glycolysis under therapeutic pressure. We assessed the energy metabolism status of the cells after repeated treatments using the Seahorse assay. The results demonstrated that the oxidative phosphorylation phenotype induced by CHCA exhibited long‐term stability (Figure 2M,N(The order here is different from other places); and Figure S34, Supporting Information). Following the addition of CHCA, the survival rate of cells was relatively low, however, after TTM treatment, it was completely restored to the level of the group without CHCA (Figure S35, Supporting Information). This demonstrated that CHCA enhances cuproptosis in Hepa1‐6 cells by regulating intracellular lactate metabolism. Interestingly, by comparing T1 and T3, as well as T2 and T4, it was found that AuNPs induced additional reduction in Hepa1‐6 cells viability, while the levels could also recover by TTM. This finding suggests that AuNPs may be involved in the process of cuproptosis. During the process of mimicking GOx, AuNPs substantially depleted intracellular glucose reserves (Figure 2O). Published studies have shown that glucose starvation can alter the mRNA expression levels of key copper transporters (ATP7A/B, SLC31A1, and SLC25A3).^[^ ^51^, ^52^ ^]^ We further evaluated the protein‐level changes of ATP7A, ATP7B, and SLC31A1 in Hepa1‐6 cells using WB. The experimental results indicated that a low‐glucose environment activated the AMPK pathway, which in turn altered the expression of these key copper transport proteins (Figure S36A, Supporting Information). Similarly, the glucose‐starved environment induced by Au nanoparticles also affected the expression of these copper transporters via the AMPK pathway (Figure S36B, Supporting Information). Therefore, we speculate that glucose starvation had a positive effect on cuproptosis. We cultured Hepa1‐6 cells in six groups (named C1, C2, C3, C4, C5, and C6), including standard medium, low‐glucose medium, Elesclomol + standard medium, Elesclomol + low‐glucose medium, Cu(II)‐Elesclomol + standard medium, and Cu(II)‐Elesclomol + low‐glucose medium, respectively. We can see that the C1 group and the C2 group had no effect on the viability, the C3 group and the C4 group reduced the cell viability. Notably, the Cu(II)‐Elesclomol in medium markedly suppressed the proliferation of Hepa1‐6 cells, while the low‐glucose environment enhanced the inhibitory effect (Figure 2P). Then, we performed WB analysis on the expression of DLAT, FDX1, and LIAS proteins in groups C1 to C6. The results showed that the decrease in cell viability in groups C5 and C6 was associated with cuproptosis. These findings indicate that low glucose disrupts intracellular copper homeostasis and promotes the process of cuproptosis (Figure S37, Supporting Information).

Subsequently, we evaluated the intensity of cuproptosis across different nanomedicines. Given that cuproptosis induces mitochondrial dysfunction, we first detected the mitochondrial membrane potential (MMP) in Hepa1‐6 cells. Among them, the red fluorescence in the PEG@AuCZ@CC NPs group almost disappeared, while the green fluorescence was intensified significantly, indicating that the mitochondria were most severely damaged (Figure S38, Supporting Information). It should be noted that ferroptosis can also induce alterations in mitochondrial membrane potential. To avoid this interference, we performed immunofluorescence detection directly of the key protein DLAT, and stained the mitochondria at the same time to clarify that the observed changes were inside the mitochondria. It can be seen that after the addition of the NPs, blocky green fluorescence began to appear within the mitochondria, and the fluorescence intensity increased significantly with the addition of gold nanoparticles. Moreover, We performed co‐localization analysis between the green fluorescence of DLAT and the red mitochondrial fluorescence, which yielded a Pearson correlation coefficient exceeding 0.7. Thus, the increase in green fluorescence intensity reflects enhanced oligomerization of DLAT within the mitochondria, corresponding to a heightened intensity of cuproptosis (Figure 2Q,R; Figure S39, Supporting Information). Collectively, these findings indicate that PEG@AuCZ@CC NPs potentiate cuproptosis in Hepa1‐6 cells through metabolic reprogramming of both lactate and glucose pathways (Figure 2S). Finally, through Western blot analysis, we systematically determined the expression levels of ferroptosis‐related factors (TFR1, SLC40A1, LC3A/B, GPX4, FTH1) and cuproptosis‐related factors (POLD1, ACO2, DLAT, LIAS, FDX1) in cells (Figure 2T,U). These results demonstrated that PEG@AuCZ@CC NPs could induce both ferroptosis and cuproptosis more efficiently by regulating glucose and lactate metabolism in Hepa1‐6 cells.

### 2.4 The Anti‐Tumor Effect of PEG@AuCZ@CC In Vitro and its Underlying Mechanism in Inhibiting Proliferation and Migration

**Figure 3:**
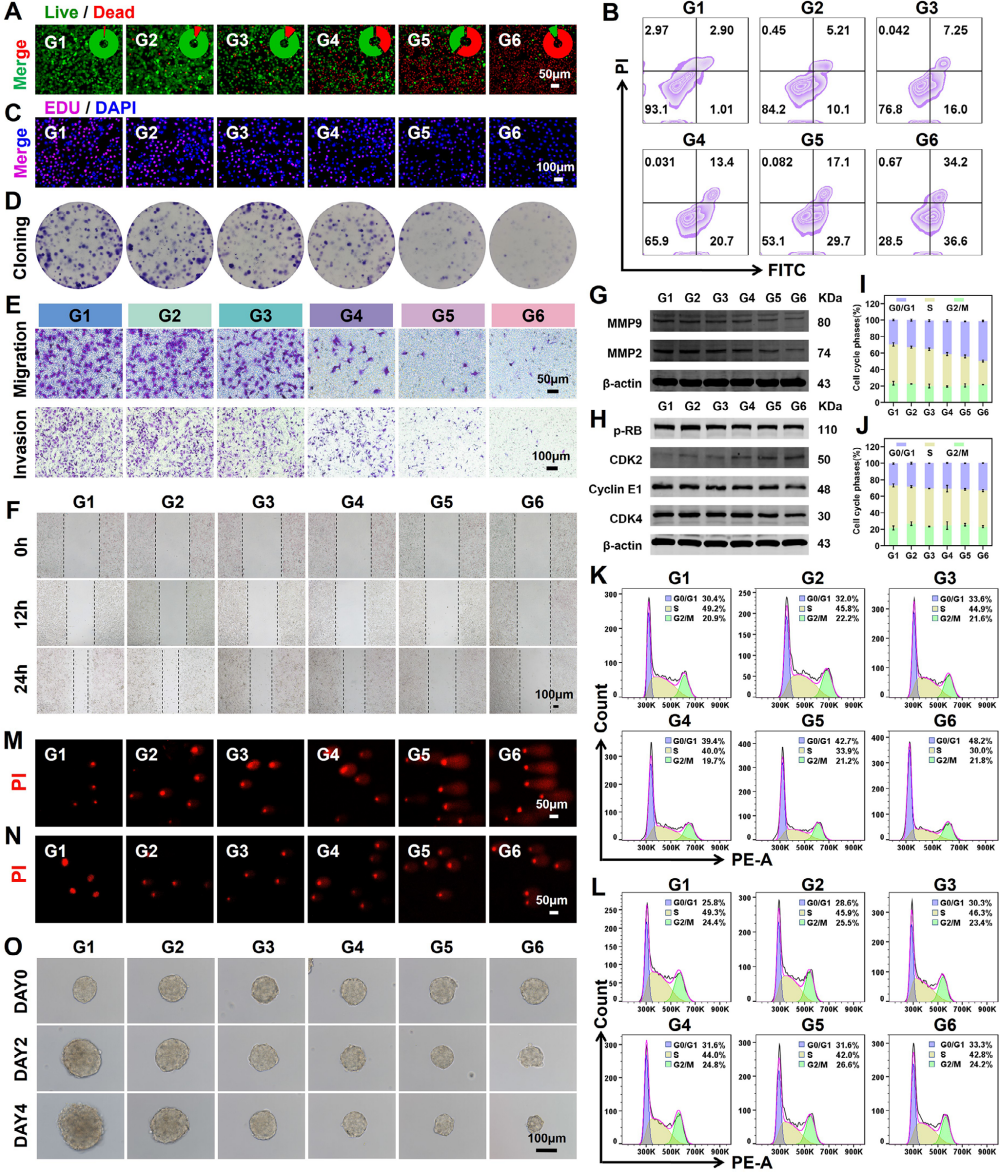
The anti‐tumor effect of PEG@AuCZ@CC in vitro and its underlying mechanism in inhibiting proliferation & migration. A) Fluorescence inverted microscopy images and Semi‐quantitative analysis of Hepa1‐6 cells stained with propidium iodide (PI) & calcein AM after different treatments. (Scale bar: 50 µm). B) Flow cytometry of Hepa1‐6 cells stained with propidium iodide (PI) & annexin V‐FITC assay kit after different treatments. C) Fluorescence inverted microscopy images of Hepa1‐6 cells stained with EDU after different treatments, wherein cell nuclei are stained with DAPI. (Scale bar: 100 µm). D) Colony test for evaluating Hepa1‐6 cells proliferation after different treatments. E) Transwell migration and invasion optical images for assessing the metastatic capability of Hepa1‐6 cells following different treatments (Scale bar: 50 and 100 µm). F) Brightfield images of wound healing assays at 0, 12, and 24 h post‐treatment (Scale bar: 100 µm). G) Western blotting analysis of MMP2 and MMP9 in Hepa1‐6 cells after different treatments. H) Western blotting analysis of p‐RB, Cyclin E1, CDK2, and CDK4 in Hepa1‐6 cells after different treatments. I,J) Quantitative data and (K, L) flow cytometry patterns of Hepa1‐6 cells stained with propidium iodide (PI) after different treatments. (M, N) Comet assay for evaluating the DNA damage of Hepa1‐6 cells after different treatments. (Scale bar: 50 µm). O) Tumor spheroid assays of Hepa1‐6 cells after different treatments. (Scale bar: 100 µm). Data are expressed as mean ± SD (*n* = 3). One‐way ANOVA or t‐test was used to analyze statistical differences between groups. ^*^ *p* <0.05, ^**^ *p* <0.01, ^***^ *p* <0.001, ^****^ *p* <0.0001. "ns" denotes no significant difference. Note, G1: Control, G2: CO, G3: CO&CHCA, G4: CZ@CC, G5: AuCZ@CC, G6: PEG@AuCZ@CC NPs.

First, we validated the tumoricidal effect of PEG@AuCZ@CC NPs by Calcein‐AM/PI staining, and a large number of dead cells with red fluorescence were observed, with only a few live cells could be detected (**Figure** **3**A). Similarly, the apoptosis rate in the PEG@AuCZ@CC NPs group exhibited statistically elevated values compared to other groups (Figure 3B; Figure S40A, Supporting Information). These results demonstrate that PEG@AuCZ@CC NPs can kill tumor cells efficiently. Subsequently, we evaluated the effect of PEG@AuCZ@CC NPs on tumor cells proliferation. During DNA synthesis, EdU competitively substitutes for thymidine in nascent DNA strands through DNA polymerase‐mediated incorporation, with red fluorescence representing proliferative signal. We found that the red fluorescence signal in the PEG@AuCZ@CC NPs group was the weakest among all groups, indicating significant suppression of Hepa1‐6 cells proliferation (Figure 3C; Figure S40B, Supporting Information). Similarly, in colony experiment, PEG@AuCZ@CC NPs could significantly inhibit the colony‐forming ability of Hepa1‐6 cells (Figure 3D; Figure S40C, Supporting Information). Transwell migration assay, transwell invasion assay, and wound healing assay clearly demonstrated that PEG@AuCZ@CC NPs impaired the motility of Hepa1‐6 cells, which is conducive to attenuating tumor invasion and metastasis (Figure 3E,F; Figure S40D–F, Supporting Information).

To further elucidate the mechanism by which PEG@AuCZ@CC NPs inhibits proliferation and motility in Hepa1‐6 cells, we reduced the drug concentration uniformly to avoid interference from direct cytotoxicity. At this time, although Hepa1‐6 cells remained adherent, their morphology changed significantly, becoming flattened and swollen, and the nucleus increased significantly (Figure S41A, Supporting Information). It can be observed that the density of Hepa1‐6 cells remained basically consistent between 12 and 48 h, indicating that the proliferation ability was significantly inhibited (Figure S41B, Supporting Information). Through Western blot analysis, we can see that the expression levels of MMP2 and MMP9 in cells were downregulated, confirming inhibition of motility (Figure 3G). At this point, FCM revealed that these Hepa1‐6 cells were arrested in the G0/G1 phase (Figure 3I,K). To clarify the mechanism of cell cycle arrest in Hepa1‐6 cells, we detected the expression of key cell cycle regulators (p‐RB, Cyclin E1, CDK2, CDK4) involved in G1/S transition by Western blot analysis. We can see that the expression levels of p‐RB, Cyclin E1, and CDK4 were downregulated, while CDK2 expression was upregulated (Figure 3H). This indicates that there is a change in the regulatory node proteins controlling the G1 to S phase transition in Hepa1‐6 cells. As is well known, cell cycle checkpoints mainly check the status of DNA.^[^ ^53^, ^54^ ^]^ Therefore, we used comet assays to discover that the DNA of these cells did indeed exhibit varying degrees of damage (Figure 3M; Figure S42A, Supporting Information). When treated with the antioxidant α‐Vitamin E, a significant reduction in DNA damage was observed (Figure 3N; Figure S42B, Supporting Information). Concurrently, the degree of cell cycle G0/G1 arrest was also decreased significantly (Figure 3J,L). These results demonstrate that PEG@AuCZ@CC NPs damages DNA by severe oxidative stress, leading to cell cycle arrest at the G0/G1 phase and subsequently inhibiting the proliferation and motility of Hepa1‐6 cells. Finally, to better simulate the effect of PEG@AuCZ@CC NPs on tumors in vivo, we cultured tumor spheroids of same size and treated with different nanomedicines. It can be seen that the number and diameter of tumor spheroids decreased most significantly in the PEG@AuCZ@CC NPs group (Figure 3O; Figure S43, Supporting Information).

In summary, PEG@AuCZ@CC NPs demonstrate dual anti‐tumor effects: potent direct cytotoxic effects against Hepa1‐6 cells, and significant G0/G1 phase arrest leading to suppression of both proliferative and migratory capacities. Clinically, during pharmacological treatment of solid tumors, intratumoral heterogeneity often results in variable drug concentrations across different tumor regions. Therefore, PEG@AuCZ@CC NPs show excellent potential for anti‐tumor applications.

### 2.5 Modifying Immune Cell Function In Vitro

**Figure 4:**
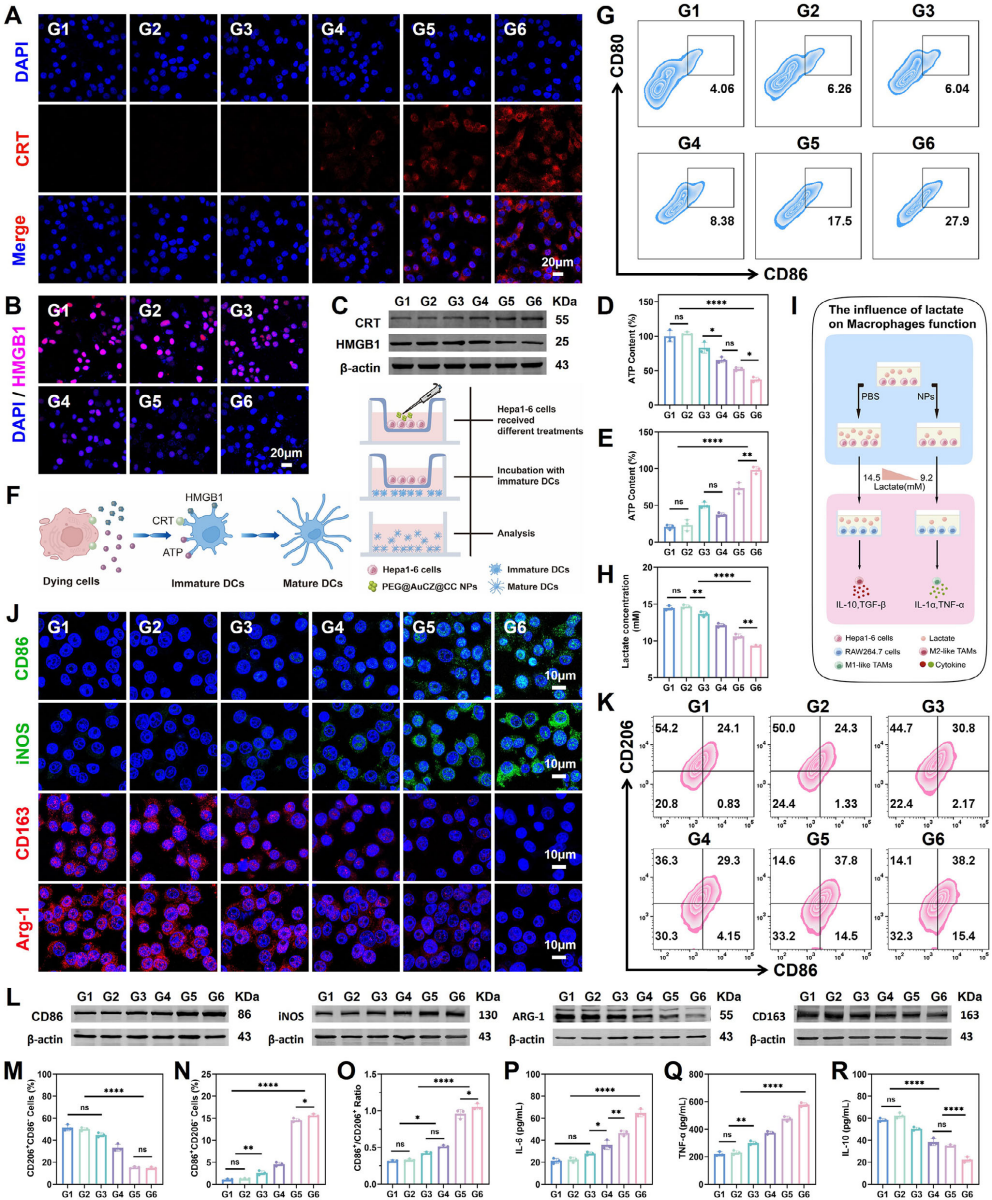
Modifying immune cell function in vitro. A) CLSM images of CRT localization on the cell membranes of Hepa1‐6 cells after different treatments, wherein cell nuclei are stained with DAPI. (Scale bar: 20 µm). B) CLSM images of HMGB1 localization in the nucleus of Hepa1‐6 cells after different treatments, wherein cell nuclei are stained with DAPI. (Scale bar: 20 µm). C) Western blotting analysis of CRT and HMGB1 in Hepa1‐6 cells after different treatments. D) Intracellular ATP level and E) extracellular ATP level of Hepa1‐6 cells under different treatments. F) Scheme of the co‐incubation system of immature DCs and Hepa1‐6 cells. G) Flow cytometry analysis of DC maturation in different treatment groups. (H) The levels of lactate measured in the Hepa1‐6 cells culture medium 24 h after various treatments. I) In vitro experimental design to examine the influence of lactate on RAW264.7 cells function. J) CLSM images of Hepa1‐6 cells stained with anti‐CD86, anti‐iNOS, anti‐CD163, and anti‐Arg‐1 after different treatments. (Scale bar: 10 µm). K) Flow cytometry analysis and M–O) Quantitative data of RAW264.7 cells in different treatment groups. L) Western blotting analysis of CD163, Arg‐1, CD86, and iNOS in RAW264.7 cells after different treatments. The secreted levels of cytokines, including P) IL‐6, Q) TNF‐α, and R) IL‐10 in different treatment groups. Data are expressed as mean ± SD (*n* = 3). One‐way ANOVA or *t*‐test was used to analyze statistical differences between groups. ^*^ *p* <0.05, ^**^ *p* <0.01, ^***^ *p* <0.001, ^****^ *p* <0.0001. "ns" denotes no significant difference. Note, G1: Control, G2: CO, G3: CO&CHCA, G4: CZ@CC, G5: AuCZ@CC, G6: PEG@AuCZ@CC NPs.

Therapeutic strategies based on ferroptosis and cuproptosis can induce immunogenic cell death (ICD). The key hallmarks of ICD include calreticulin (CRT) membrane translocation, high mobility group box 1 (HMGB1) secretion, and adenosine triphosphate (ATP) release. As shown by immunofluorescence, after PEG@AuCZ@CC NPs treatment, only weak HMGB1 fluorescence signals were observed in Hepa1‐6 cells, while the fluorescence intensity of CRT was the most significant (**Figure** **4**A,B; Figure S44, Supporting Information). Western blot analysis revealed consistent trends with the immunofluorescence results (Figure 4C). Furthermore, the intracellular ATP level in the PEG@AuCZ@CC NPs group decreased to 36.33% of the control group, while the ATP level in the culture medium increased to 477.18% of the control group (Figure 4D,E). These results demonstrate that PEG@AuCZ@CC NPs can trigger the ICD of Hepa1‐6 cells effectively. Next, we isolated primary dendritic cells (DCs) from the femurs of C57BL/6 mice and co‐incubation with Hepa1‐6 cells from different treatment groups. The debris of Hepa1‐6 cells released abundant tumor‐associated antigen, which then crossed the chamber membrane to promote DCs maturation (Figure 4F). Thus, after 24 h of co‐incubation, we can see that the morphological transformation of rounded immature DCs to mature DCs with obvious dendrites (Figure S45, Supporting Information). Subsequently, we used FCM to assess DC maturation of each treatment group. It can be seen that PEG@AuCZ@CC NPs effectively induced DC maturation with a proportion of 27.90%, which was significantly higher than other groups (Figure 4G; Figure S46, Supporting Information). We also quantified the secretion levels of immunostimulatory cytokines (IL‐6, IL‐12, TNF‐α, and IFN‐γ) by ELISA. The results showed that PEG@AuCZ@CC NPs significantly upregulated these cytokines compared to other treatment groups (Figure S47, Supporting Information). These findings demonstrate that PEG@AuCZ@CC NPs‐mediated ICD effect induced DC maturation for immunostimulatory cytokines secretion.

Hepa1‐6 cells lead to a large accumulation of lactate in the TME, which polarizes tumor‐associated macrophages (TAMs) into M2 phenotype to facilitate tumor progression. In our previous studies, we have demonstrated that PEG@AuCZ@CC NPs can inhibit lactate efflux by inhibiting the function of MCT 1/2/4 in Hepa1‐6 cells. Subsequently, we quantified lactate levels in culture medium to evaluate the alteration in the tumor extracellular environment. Compared with the control group (lactate: 14.5 ± 0.3 mm), the lactate concentration after PEG@AUCZ@CC NPs treatment was 9.2 ± 0.4 mm (Figure 4H). This suggests that PEG@AUCZ@CC NPs can ameliorate the high lactate in the TME, establishing a lactate‐depleted microenvironment. On this basis, to further validate macrophage phenotypic alterations, we established a co‐culture system of RAW264.7 cells with Hepa1‐6 cells from different treatments (Figure 4I). It can be seen that PEG@AuCZ@CC NPs could significantly down‐regulate the expressions of Arg‐1 and CD163 that are the characteristic markers of M2 TAMs. In contrast, PEG@AuCZ@CC NPs received the highest expressions of M1 TAMs biomarkers such as iNOS and CD86 (Figure 4J; Figure S48, Supporting Information). Western blot analysis obtains identical results. We can see that the expression levels of CD163 and Arg‐1 were downregulated, while CD86 and iNOS expression was upregulated (Figure 4L). Furthermore, Flow cytometry analysis demonstrated that PEG@AuCZ@CC NPs group exhibited the lowest M2 phenotype frequency (CD206^+^CD86^‐^) and the highest M1 phenotype frequency (CD86^+^CD206^‐^) among all treatment groups (Figure 4K,M–O[‐](The order here is different from other places)). Finally, cytokines profiles were analyzed using ELISA kits, demonstrating enhanced production of tumor‐suppressive cytokines (IL‐6, TNF‐α) post‐therapy, while the pro‐tumorigenic cytokine (IL‐10) was inhibited, particularly PEG@AuCZ@CC NPs (Figure 4P–R). These results demonstrate that PEG@AuCZ@CC NPs reprogram the tumor immunosuppressive microenvironment by inhibiting lactate efflux, thereby reversing the polarization of M2‐type TAMs to M1‐type TAMs and ultimately restoring the immune role of macrophages.

Similarly, studies have shown that Treg cells preferentially utilize tumor‐derived lactate as an energy source, demonstrating reduced dependence on glucose.^[^ ^55^, ^56^ ^]^ Consequently, restricting lactate supply compromises Treg cell stability.^[^ ^57^ ^]^ To validate the impact of lactate on Treg cells, we assessed Treg cells functionality through inhibition assays and IL‐10/TGF‐β secretion analysis. First, we isolated Treg cells from the spleens of C57 mice, purified and cultured them, then incubated them for 24 h in media with varying glucose concentrations (5, 10, 15, 20 mm) and lactate concentrations (0, 5, 10, and 15 mm). The levels of IL‐10 and TGF‐β in the supernatant were measured using ELISA. The results indicated that changes in lactate concentration had a more pronounced effect on Treg cells function compared to changes in glucose levels (Figure S49, Supporting Information). Next, we collected Treg cells cultured under different lactate concentrations and co‐cultured them with CTLL‐2 cells for 24 h. The activity of CTLL‐2 cells was evaluated by flow cytometry analysis of CD4+IFN‐γ+ and CD8+IFN‐γ+ expression. It was observed that Treg cells from low‐lactate conditions gradually lost their suppressive effect on CTLL‐2 cells, indicating that lactate depletion inhibits Treg cells function (Figure S50, Supporting Information).

Given that PEG@AuCZ@CC NPs can attenuate glycolysis and inhibit lactate efflux, we further investigated its impact on Treg cells. First, Hepa1‐6 cells were pretreated with different drugs for 24 h, after which the culture medium was replaced. Treg cells were then placed in a transwell insert and co‐cultured with the pretreated Hepa1‐6 cells for 24 h. Analysis of the collected supernatant showed a reduction in lactate levels in the G6 group, accompanied by decreased levels of IL‐10 and TGF‐β (Figure S51, Supporting Information). Next, Treg cells from the transwell system were co‐cultured with CTLL‐2 cells. The CTLL‐2 cells were collected at 24 and 48 h, their proliferation was assessed using CFSE‐based flow cytometry (Figure S52, Supporting Information). And their functional activity was evaluated by measuring CD4+IFN‐γ+ and CD8+IFN‐γ+ expression via flow cytometry (Figure S53, Supporting Information). Compared to other treatment groups, the G6 group exhibited stronger CTLL‐2 proliferation and enhanced functional activity. These results indicate that by reducing glycolysis and lactate export in Hepa1‐6 cells, PEG@AuCZ@CC NPs lower the lactate levels in the culture medium, thereby significantly suppressing Treg cell function.

In summary, PEG@AuCZ@CC NPs not only induce immunogenic cell death (ICD) but also promote macrophage polarization toward the M1 phenotype while simultaneously suppressing Treg function. Consequently, PEG@AuCZ@CC NPs demonstrate excellent potential for remodeling the immunosuppressive tumor microenvironment and advancing anti‐tumor immunotherapy.

### 2.6 In Vivo Distribution, Biosafety, and In Vivo Antitumor Effect

**Figure 5:**
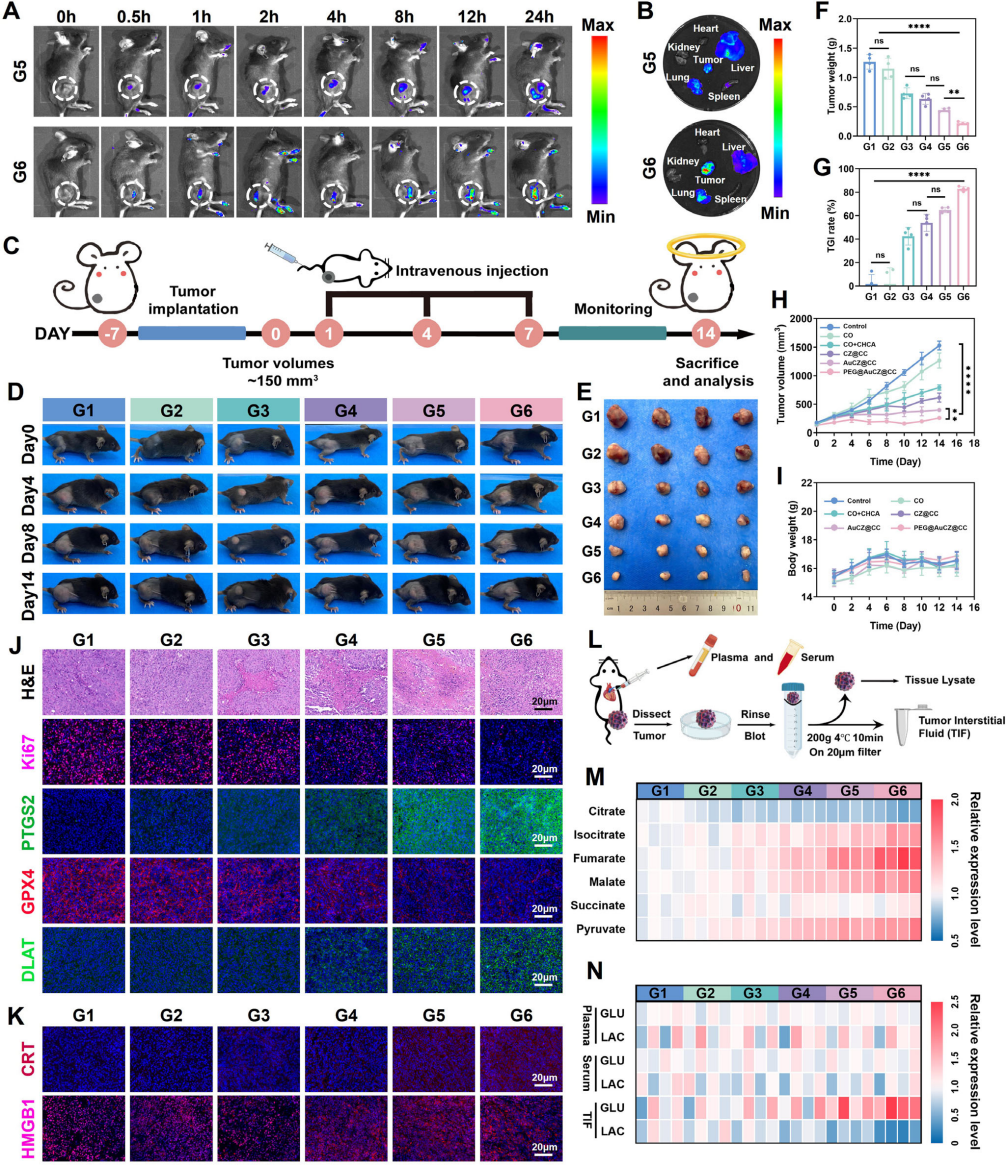
In vivo antitumor evaluations of PEG@AuCZ@CC NPs. A) Time‐dependent fluorescence images in vivo of mice bearing Hepa1‐6 tumor after injecting Cy5.5‐labeled AuCZ@CC NPs and Cy5.5‐labeled PEG@AuCZ@CC NPs. B) ex vivo fluorescence images of collected tumor and normal organs after 24 h post‐injection. C) Schematic on in vivo anti‐tumor procedures. D) Pictures of Hepa1‐6 tumor‐bearing mice that experienced different treatments in G1‐G6 on Day 0, Day 4, Day 8, and Day 14. E) Digital photograph of tumors after diverse treatments. F) Tumor weights collected on Day 14 in G1‐G6. (G) TGI rates in each group. H) Tumor volumes in each group as a function of time in G1‐G6 that experienced corresponding treatments. I) Time‐dependent body weight of mice in G1‐G6. (J) H&E, Ki67 immunofluorescence images, PTGS2 immunofluorescence images, GPX4 immunofluorescence images, and DLAT immunofluorescence images of collected tumor slices in G1‐G6 on Day 14. (Scale bar: 20 µm). K) Immunofluorescence images of CRT and HMGB1 in tumor tissues after various treatments. (Scale bar: 20 µm). L) Investigating metabolic reprogramming in tumors and its impact on the tumor microenvironment. M) Alterations in the expression of intermediate metabolites related to oxidative phosphorylation and glycolysis across treatment groups. (N) Glucose and lactate levels in serum, plasma, and tumor interstitial fluid after various treatments. Data are expressed as mean ± SD (*n* = 3). One‐way ANOVA or t‐test was used to analyze statistical differences between groups. ^*^ *p* <0.05, ^**^ *p* <0.01, ^***^ *p* <0.001, ^****^ *p* <0.0001. "ns" denotes no significant difference. Note, G1: Control, G2: CO, G3: CO&CHCA, G4: CZ@CC, G5: AuCZ@CC, G6: PEG@AuCZ@CC NPs.

Based on the in vitro experiments, we further investigated the anti‐tumor effects of different nanomedicines in vivo. Before this, considering the systemic administration route via intravenous injection, we co‐incubated the red blood cells with different nanomedicines at 37°C for 4 h. Results demonstrated that none of nanomedicines induced hemolysis (Figure S54, Supporting Information). Then, different concentrations of PEG@AuCZ@CC NPs were co‐incubated with red blood cells. It can be seen that the hemolytic effect of PEG@AuCZ@CC NPs is negligible even at 300 µg mL^−1^ (Figure S55A, Supporting Information). Microscopic examination revealed that RBCs maintained their typical biconcave discoid morphology, indicating excellent hemocompatibility of PEG@AuCZ@CC NPs (Figure S55B, Supporting Information). Subsequently, Hepa1‐6‐LUC cells were implanted subcutaneously in the right dorsal flank of mice to establish localized tumors for longitudinal monitoring. Prior to drug treatment, the in vivo pharmacokinetics and tumor‐targeting efficiency of PEG@AuCZ@CC NPs were systematically evaluated in murine models. We injected two sets of nanomedicines (Cy5.5‐labeled AuCZ@CC NPs and Cy5.5‐labeled PEG@AuCZ@CC NPs) into mice via tail vein, and imaged by in vivo imaging system (IVIS) at various time points (0, 0.5, 1, 2, 4, 8, 12, and 24 h). It could be observed that the fluorescence signal at the tumor gradually increased after 0.5 h of tail vein injection. Moreover, compared with the AuCZ@CC/Cy5.5 NPs (G5), the cumulative fluorescence intensity was significantly higher in PEG@AuCZ@CC/Cy5.5 NPs (G6) (**Figure** **5**A). This result is closely associated with the DSPE‐PEG2000‐GA, which improves the tumor targeting ability of the nanomedicine in mice. To further investigate its metabolism in vivo, we euthanized the mice, and vital organs (heart, liver, spleen, lung, kidney) and neoplastic tissues were collected for quantitative biodistribution assessment. The fluorescence signal was predominantly observed in the tumor tissue, while also concentrated in the liver and lung, indicating that the nanomedicines also accumulated and enriched in the liver and lung (Figure 5B). The pulmonary accumulation may be attributed to the abundant capillaries, which prolongs the residence time of nanomedicines. To rule out the possibility that the high accumulation of nanoparticles in the lungs was due to capillary trapping leading to target effects in the lungs, we extended the observation time to 48 h (with groups established at 24, 36, and 48 h). It can be observed that the fluorescence in the lungs had significantly weakened by the 36 h time point and completely disappeared by the 48 h time point, while the tumor site still exhibited high fluorescence intensity (Figure S56, Supporting Information). This indicates that the high accumulation of PEG@AuCZ@CC in the lungs may be related to the abundant capillaries in the lungs, which prolonged the retention time of the drug, rather than being an off‐target effect. Furthermore, considering the prolonged retention time of PEG@AuCZ@CC in the lungs, we selected mouse lung epithelial cells (MLE‐12) and mouse vascular smooth muscle cells (MOVAS) to evaluate the biosafety of PEG@AuCZ@CC (Figure S57, Supporting Information). It was observed that even after 48 h of treatment with 120 µg mL^−1^ of PEG@AuCZ@CC NPs, more than 75% of AML12 and THLE‐2 cells remained viable, indicating that PEG@AuCZ@CC has negligible targeting and toxicity toward alveolar epithelial cells and vascular smooth muscle cells. Meanwhile, hepatic accumulation indicates the nanomedicine is primarily metabolized by the liver, which is the typical metabolic pathway for drugs. Notably, PEG@AuCZ@CC NPs exhibited high accumulation in tumor site, which can meet the basic therapeutic requirements. Therefore, the mice were randomly divided into six groups: Control, CO, CO+CHCA, CZ@CC, AuCZ@CC, and PEG@AuCZ@CC. Then, systemic delivery of nanotherapeutics was achieved through triweekly intravenous administrations (days 1, 4, and 7) via the lateral tail vein (Figure 5C). By taking photographs and recording tumor volume and mouse weight at different time intervals, we were able to monitor tumor growth over time (Figure 5D). At the end of the treatment, the mice were euthanized, and tumors were excised from each mouse. As expected, via assessed tumor growth profiles and collected tumor weights, we found that the tumor size and weight in the PEG@AuCZ@CC NPs group were the lowest among all groups, with a tumor suppression rate of 85% (Figure 5E–G). Throughout the entire treatment period, tumor volumes in all treatment groups were smaller than those in the control group, indicating that all treatments exhibited varying degrees of tumor inhibitory effects. Among these, the PEG@AuCZ@CC NPs group demonstrated markedly slower tumor growth compared to other groups (Figure 5H; Figure S58, Supporting Information). Importantly, weight fluctuations of mice after different treatments were negligible (Figure 5I; Figure S59, Supporting Information). Simultaneously, PEG@AuCZ@CC NPs significantly prolonged survival duration and achieved the highest survival rate among all treatment groups (Figure S60, Supporting Information). These results indicate that PEG@AuCZ@CC NPs have good anti‐tumor effect and high biological safety.

The condition of tumor necrosis can be fully demonstrated at the cellular level. H&E staining revealed that cells in the control group maintained intact nuclear structures and cell membranes, indicating that the tumors had high activity and invasiveness. Among the treatment groups, PEG@AuCZ@CC NPs exhibited the most severe cellular damage, characterized by membrane rupture, nuclear pyknosis, karyorrhexis, and karyolysis. Consistent results were observed in TUNEL immunofluorescence staining, confirming that PEG@AuCZ@CC NPs induced the most significant destruction on tumors (Figure S61, Supporting Information). PEG@AuCZ@CC NPs group also showed the weakest Ki67 fluorescence signals, suggesting a marked suppression of cellular proliferation capacity. Furthermore, in order to verify the mechanism of tumor killing by PEG@AuCZ@CC NPs in vivo is consistent with the cellular level, we performed immunofluorescence analysis of ferroptosis markers (PTGS2, GPX4) and cuproptosis marker (DLAT). We can see that the fluorescence intensity of PTGS2 increased significantly, while the fluorescence intensity of GPX4 gradually decreased. Notably, the fluorescence intensity of DLAT not only increased but also showed clustering, indicating that the oligomerization of DLAT increased (Figure 5J). Meanwhile, it can be seen that the expression levels of GPX4, and non‐oligomerized DLAT were downregulated significantly by Western blot analysis (Figure S62, Supporting Information). Collectively, these results demonstrate that PEG@AuCZ@CC NPs efficiently kills tumor cells through both ferroptosis and cuproptosis. Therapeutic strategies based on ferroptosis and cuproptosis can induce immunogenic cell death (ICD). Thus, we can observe in the immunofluorescence images that CRT exposure gradually intensified, while the fluorescence intensity of HMGB1 progressively shifted from the nucleus to the cytoplasm and was eventually released extracellularly in the PEG@AuCZ@CC NPs group (Figure 5K; Figure S63, Supporting Information). Under the stimulation of these DAMPs, flow cytometry analysis revealed a significant maturation of dendritic cells (DCs) (Figure S64, Supporting Information).

### 2.7 In Vivo Antitumor Immunotherapy

**Figure 6:**
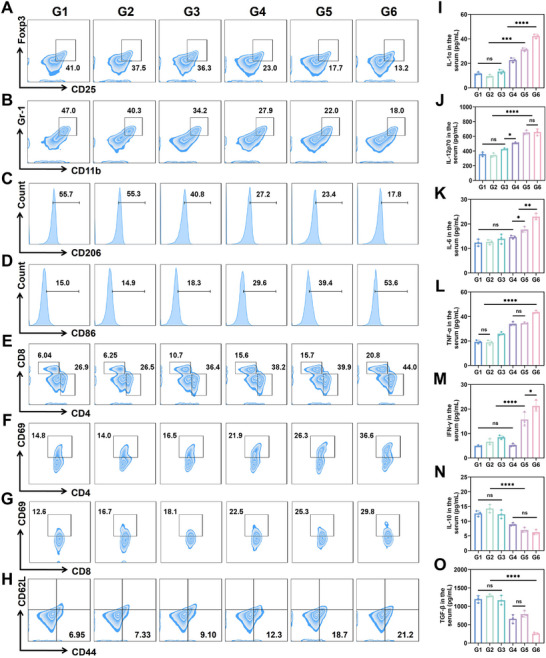
In vivo antitumor immunotherapy. A) Flow cytometry analysis of Treg cells in the tumor of mice receiving various treatments. B) Flow cytometry analysis of tumor MDSCs. Flow cytometry analysis of C) M2 TAMs and D) M1 TAMs in Hepa1‐6 tumors of mice after various treatments. E) Flow cytometry analysis of CD4^+^ T cells and CD8^+^ T cells in Hepa1‐6 tumor receiving various treatments. Flow cytometry analysis of F) CD4^+^CD69^+^ T cells and G) CD8^+^CD69^+^ T cells in Hepa1‐6 tumor receiving various treatments. H) Flow cytometry analysis of effector memory T cells in the spleen of mice receiving various treatments. I–O) The levels of different cytokines secretion in mouse serum after various treatments. Data are expressed as mean ± SD (*n* = 3). One‐way ANOVA or *t*‐test was used to analyze statistical differences between groups. ^*^ *p* <0.05, ^**^ *p* <0.01, ^***^ *p* <0.001, ^****^ *p* <0.0001. "ns" denotes no significant difference. Note, G1: Control, G2: CO, G3: CO&CHCA, G4: CZ@CC, G5: AuCZ@CC, G6: PEG@AuCZ@CC NPs.

In our in vitro studies, we have observed that metabolic reprogramming of tumor cells contributes to improve the environment of the cell culture supernatant. Therefore, we collected serum, plasma, tumor interstitial fluid (TIF), and tumor tissue samples for analysis to determine whether metabolic reprogramming occurred in vivo tumors and whether changes in the tumor microenvironment were induced by different treatments (Figure 5L). First, analysis of tumor tissue samples from different treatment groups revealed that PEG@AuCZ@CC enhanced the expression of oxidative phosphorylation‐related intermediate metabolites while reducing the expression of glycolysis‐related intermediate metabolites (Figure 5M). This indicates that the energy metabolism of tumor cells shifted toward oxidative phosphorylation. Next, we measured changes in glucose and lactate levels in serum, plasma, and tumor interstitial fluid. Compared to serum and plasma, the lactate levels in the tumor interstitial fluid decreased, while glucose levels increased, suggesting an improvement in the TME (Figure 5N; Figure S65, Supporting Information). These findings demonstrate that PEG@AuCZ@CC successfully improved the TME by inducing metabolic reprogramming in tumor cells, which is critical for restoring the function of immune cells. Therefore, we observed the proportion of immunosuppressive cells were significantly reduced, with Treg cells decreased from 41.0% to 13.2% and MDSCs decreased from 47.0% to 18.0% compared to control group (**Figure** **6**A,B; Figure S66, Supporting Information). At the same time, flow cytometry and immunohistochemistry (IHC) analyses demonstrated that PEG@AuCZ@CC NPs promoted the polarization of M2 TAMs into M1 ones, and increase the ratio of M1/M2 (Figure 6C,D; Figures S67 and S68, Supporting Information). M1 TAMs and mDCs (activated by ICD) are known to phagocytose tumor antigens and play crucial roles in recruiting and priming T cells. Therefore, significant infiltration of both CD4^+^ T cells and CD8^+^ T cells were observed in tumor tissues via immunofluorescence and flow cytometry (Figure 6E; Figures S69 and S70, Supporting Information). Meanwhile, a greater number of CD4^+^ T cells and CD8^+^ T cells were activated (Figure 6F,G; and Figure S71, Supporting Information). Notably, the proportion of effector memory T cells in the PEG@AuCZ@CC NPs group was the highest by measuring the number of memory T cells in the spleen after different treatments (Figure 6H; Figure S72, Supporting Information). Correspondingly, the cytokines secreted by these immune cells also changed. In detail, some anti‐tumorigenic cytokines, including IL‐1α, IL‐12p70, IL‐6, TNF‐α, and IFN‐γ increase, while the pro‐tumorigenic IL‐10 and TGF‐β are inhibited (Figure 6I–O).

Considering that IL‐6 and TNF‐α are inflammatory factors, it is essential to determine whether their presence stems from the tumor‐inhibitory effects or from excessive activation of systemic inflammation. To first rule out the possibility that PEG@AuCZ@CC NPs induces an acute inflammatory response, we established a systemic inflammatory mouse model (SRIS) via intraperitoneal injection of LPS (5 and 10 mg kg^−1^) as an acute inflammation control group. Changes in body weight and behavior were observed across groups, and mice were euthanized at 12 h to analyze serum levels of IL‐6, TNF‐α, and IL‐1β. The results showed that mice in the acute inflammation control group exhibited decreased body weight and behavioral scores, along with a sharp increase in serum IL‐6, TNF‐α, and IL‐1β. In contrast, neither the PBS control group nor the PEG@AuCZ@CC NPs treatment group showed such abnormalities (Figure S73, Supporting Information). Next, to exclude the possibility that PEG@AuCZ@CC NPs induces subacute/chronic inflammatory responses, we established subacute/chronic inflammation control groups by continuously administering 0.3 mg kg^−1^ LPS aqueous solution via intraperitoneal injection (named as the LPS‐Water group) and implanting sterile cotton balls into the groin region of mice (named as the CP group). Changes in body weight and behavior were monitored across all groups. At 2/4/8/12/14 day, the mice were euthanized, and serum levels of IL‐6, TNF‐α, and IL‐1β were analyzed. The results showed that mice in the PEG@AuCZ@CC NPs treatment group maintained stable body weight and behavioral scores. In contrast, mice in the subacute/chronic inflammation control groups exhibited decreased body weight and behavioral ratings. Although serum levels of IL‐6, TNF‐α, and IL‐1β increased transiently between days 4 and 12 in the PEG@AuCZ@CC NPs group, the magnitude of increase was significantly lower than that observed in the subacute/chronic inflammation control groups (Figure S74, Supporting Information). As is known, inflammation is often accompanied by changes in hematological parameters such as neutrophils, monocytes, and lymphocytes. Therefore, at three time points (days 4, 8, and 12), we adopted clinically relevant inflammatory evaluation metrics — NLR (neutrophils / lymphocytes), SII (platelets × neutrophils / lymphocytes), SIRI (neutrophils × monocytes / lymphocytes), and ALT/AST ratios — to assess systemic inflammation. The results showed no significant differences between the PEG@AuCZ@CC NPs treatment group and the PBS control group in these indices (Figure S75, Supporting Information). Therefore, PEG@AuCZ@CC NPs did not induce a systemic inflammatory response. The observed increase in IL‐6 and TNF‐α levels likely originated from immune cells suppressing tumor growth, which further demonstrates the excellent ability of PEG@AuCZ@CC NPs to activate anti‐tumor immunity.

These results demonstrate that PEG@AuCZ@CC NPs can enhance the intensity of ICD triggered by ferroptosis and cuproptosis, reshape the inhibitory immune microenvironment, and suppress the activity of immunosuppressive cells. On this basis, the activation and effector function of CD4^+^/CD8^+^ T cells were enhanced, thereby enhanced killing of tumors. Meanwhile, a increase in memory T cells was also observed. This clearly facilitates the formation of immune surveillance and immune memory, which is important for inhibiting tumor recurrence and metastasis. To investigate the long‐term immune effects after treatment, a tumor rechallenge model was established. Finally, the spleens of the mice were collected, and changes in effector memory T cells (Tem, CD8⁺CD44⁺CD62L^−^) were assessed using flow cytometry. Compared with the control group, PEG@AuCZ@CC NPs treatment increased the proportions of Tem by 2.9‐fold (Figure S76, Supporting Information). In conclusion, PEG@AuCZ@CC NPs plays a critical role in inducing immune memory and suppressing tumor rechallenge. Considering the ultimate goal of translating the research into clinical applications, we evaluated the effect of PEG@AuCZ@CC on the human‐derived hepatocellular carcinoma cell line HuH‐7. It was observed that the tumor size and weight in the PEG@AuCZ@CC group were the lowest among all groups, with a tumor inhibition rate of 75.78%. Throughout the treatment period, tumor growth in the PEG@AuCZ@CC group was significantly slower compared to the control and other treatment groups, and fluctuations in mouse body weight were negligible (Figure S77, Supporting Information).

During all experiments, mice in all groups maintained consistent fur condition, mental status, food intake, defecation, and activity levels. It is worth noting that before the experiment, we evaluated the biological safety of different treatments in vivo. Histological examination via H&E staining demonstrated no evident morphological changes in key organs (heart, liver, spleen, lung, and kidney) among the different groups (Figure S78, Supporting Information). Meanwhile, hematological parameters were evaluated (WBC, RBC, PLT, HGB, MCV, ALT, AST, TBIL, CREA, and UA), and the results showed that the functional indexes of mice in PEG@AuCZ@CC NPs group were within the safe range compared with those in the control group (Figure S79, Supporting Information). Considering the catalytic activity and redox modulation capability of the nanozyme system, we performed histological marker detection (4‐HNE and 8‐OHdG staining) and functional enzyme activity assays (SOD, CAT, GSH‐Px, and MDA) in the liver, kidney, and heart tissues. The results indicate that PEG@AuCZ@CC NPs did not cause oxidative damage to these healthy organs with high metabolic activity (Figure S80, Supporting Information). Next, we further evaluated the long‐term safety of PEG@AuCZ@CC. First, the observation period was extended to 30 days, and H&E staining of the heart, liver, spleen, lungs, and kidneys revealed no significant histological changes. Blood was also collected from the mice for analysis (WBC, RBC, PLT, NEUT, HGB, MCV, CK‐MB, LDH, ALT, AST, CREA, and UA). Additionally, no deposition of zinc or copper was observed in the heart, liver, spleen, lungs, or kidneys via the inductively coupled plasma mass spectrometry (ICP‐MS) technology analysis (Figure S81, Supporting Information). Collectively, the rationally designed therapeutic platform exhibits: i) potent tumor‐suppressive effects, ii) favorable biosafety profiles with no observable toxicity, and iii) optimal tissue compatibility, positioning it as a promising candidate for translational cancer immunotherapy.

## 3 Conclusion 

In conclusion, we successfully synthesized organometallic framework PEG@AuCZ@CC nanomaterials to induce ferroptosis and cuproptosis‐associated immunotherapy and TME reprogramming. Specifically, CHCA (a monocarboxylate transporter inhibitor) suppressed cancer glycolytic metabolism, and Au nanozymes implemented starvation therapy specifically in cancer cells. Based on this, PEG@AuCZ@CC NPs recreated a high‐glucose but low‐lactate TME, which benefited the TAMs epigenetics alteration and CD4^+^/CD8^+^ T cells effector function. At the same time, to further enhance tumor antigen presentation and recruit more CD4^+^/CD8^+^ T cells, PEG@AuCZ@CC NPs efficiently promoted ICD by inducing a multiple‐death strategy (ferroptosis and cuproptosis). The enhanced ATP, HMGB1, and CRT release, three typical DAMP signals, from cancer cells stimulated DCs maturation, facilitating more tumor infiltration by effective T cells. Last but not least, alterations in intracellular glucose and lactate metabolism exhibited remarkable capacity to amplify ferroptosis and cuproptosis, which could further amplify the influence of ICD. In vivo studies suggested that PEG@AuCZ@CC NPs could activate robust immune responses and recover immunological surveillance, eventually inhibit tumor progression. Such synergistic effects eventually induced 85.09% of tumor growth inhibition with high specificity and low systemic toxicity, provided a promising pathway for tumor growth inhibition and immunotherapy in hepatocellular carcinoma. In this study, we demonstrated that PEG@AuCZ@CC NPs impair DNA integrity, induce cell cycle arrest, and ultimately suppresses cellular proliferation and motility. However, since cell cycle arrest is also closely associated with senescence.^[^ ^58^, ^59^, ^60^ ^]^ Therefore, the relationship between PEG@AuCZ@CC NPs and cellular senescence has not been fully elucidated. Meanwhile, we observed that Hepa1‐6 cells exhibit heightened sensitivity to copper ions under low‐glucose conditions, suggesting that glucose starvation potentiates cuproptosis. Nevertheless, the underlying mechanisms remain to be fully elucidated and warrant further exploration. Additionally, although PEG@AuCZ@CC NPs significantly promoted the degeneration of tumor, but the regulations of glucose and lactate were dependent on the Warburg effect. In fact, not all tumor cells possess the Warburg effect.^[^ ^61^, ^62^, ^63^ ^]^ Thus, we hope to explore more universal and simple metabolic regulation methods in future studies, which will provide more effective and safe therapeutic strategy for the HCC and other tumors.

## Conflict of Interest

The authors declare no conflict of interest.

## Author Contributions

J.L. and K.H. contributed equally to this work. J.L., K.H., D.W., and D.W. performed conceptualization. J.L., K.H., and X.Y. performed data curation. J.L., K.H., P.L., Q.Z., and F.H. performed formal analysis. J.L., X.Y., and H.X. performed the investigation. J.L., K.H., X.Y., P.L., W.L., and Q.Z. performed methodology. J.L. and F.H. performed visualization. J.L. wrote the original draft. K.H., D.W., and L.W. performed writing—review and editing. X.Y. and H.X. validate software. X.Y. performed validation. W.L., D.W., and L.W. performed funding acquisition. F.H. performed visualization. D.W. and L.W. performed project administration. D.W. and L.W. performed supervision.

## Ethical Approval

All C57BL/6 mice (from Experimental Animal Center, Guilin Medical University) were utilized following ethical approval (GLMC202303112) from the Ethics Committee of the Animal Laboratory of Guilin Medical University. All animal experiments strictly adhered to China's national laboratory animal welfare standards (GB/T35892‐2018) and the Ministry of Science and Technology's ethical guidelines for humane treatment of research animals.

## Supporting information



Supporting Information

## Data Availability

The data that support the findings of this study are available from the corresponding author upon reasonable request.

## References

[1] Y. Ling , X. Liang , K. Yan , G. Zeng , X. Zhu , J. Jiang , S. Lu , X. Wang , Y. Zhou , Z. Li , W. Mai , D. Wang , J. Chen , Adv. Sci. 2025, 12, 2500670. 10.1002/advs.202500670 PMC12199322 40305756

[2] G. Zeng , X. Liang , Y. Ling , X. Zhu , Q. Wang , Z. Li , J. Liu , X. Wang , G. Qiu , K. Yan , D. Wang , J. Chen , Biomaterials 2025, 315, 122944. 39500110 10.1016/j.biomaterials.2024.122944

[3] G. S. Feng , K. L. Hanley , Y. Liang , X. Lin , Hepatology 2021, 73, 104. 10.1002/hep.31479 PMC7854886 32715491

[4] X. Liang , D. Wang , Y. Zhao , X. Wang , S. Yao , W. Huang , Y. Yang , X. Dong , L. Zhang , J. Yang , J. Nanobiotechnol. 2024, 22, 535. 10.1186/s12951-024-02809-6 PMC11373498 39227944

[5] K.‐Y. Shen , Y. Zhu , S.‐Z. Xie , L.‐X. Qin , J. Hematol. Oncol. 2024, 17, 25. 38679698 10.1186/s13045-024-01549-2 PMC11057182

[6] J. Wu , Y. T. Chan , Y. Lu , N. Wang , Y. Feng , Med. Res. Rev. 2023, 43, 1946. 37102365 10.1002/med.21967

[7] S. Gao , X. Yang , J. Xu , N. Qiu , G. Zhai , ACS Nano 2021, 15, 12567. 34339170 10.1021/acsnano.1c02103

[8] Q. Wang , X. Zhu , B. Yin , K. Yan , G. Qiu , X. Liang , R. Jia , J. Chen , X. Wang , Y. Wu , J. Liu , J. Zhong , K. Zhang , D. Wang , Adv. Funct. Mater. 2024, 34, 2408141.

[9] Y. Yin , X. Jiang , L. Sun , H. Li , C. Su , Y. Zhang , G. Xu , X. Li , C. Zhao , Y. u. Chen , H. Xu , K. Zhang , Nano Today 2021, 36, 101009.

[10] Q. Chen , C. Wang , X. Zhang , G. Chen , Q. Hu , H. Li , J. Wang , D. i. Wen , Y. Zhang , Y. Lu , G. Yang , C. Jiang , J. Wang , G. Dotti , Z. Gu , Nat. Nanotechnol. 2019, 14, 89. 30531990 10.1038/s41565-018-0319-4

[11] P.‐C. Ho , J. D. Bihuniak , A. N. Macintyre , M. Staron , X. Liu , R. Amezquita , Y.‐C. Tsui , G. Cui , G. Micevic , J. C. Perales , S. H. Kleinstein , E. D. Abel , K. L. Insogna , S. Feske , J. W. Locasale , M. W. Bosenberg , J. C. Rathmell , S. M. Kaech , Cell 2015, 162, 1217. 26321681 10.1016/j.cell.2015.08.012 PMC4567953

[12] C.‐H. Chang , J. Qiu , D. O'Sullivan , M. D. Buck , T. Noguchi , J. D. Curtis , Q. Chen , M. Gindin , M. M. Gubin , G. J. W. van der Windt , E. Tonc , R. D. Schreiber , E. J. Pearce , E. L. Pearce , Cell 2015, 162, 1229. 26321679 10.1016/j.cell.2015.08.016 PMC4864363

[13] X. Zhu , T. Li , Q. Wang , K. Yan , S. Ma , Y. Lin , G. Zeng , J. Liu , J. Cao , D. Wang , ACS Nano 2024, 18, 32818. 39528907 10.1021/acsnano.4c11257

[14] A. Brand , K. Singer , G. E. Koehl , M. Kolitzus , G. Schoenhammer , A. Thiel , C. Matos , C. Bruss , S. Klobuch , K. Peter , M. Kastenberger , C. Bogdan , U. Schleicher , A. Mackensen , E. Ullrich , S. Fichtner‐Feigl , R. Kesselring , M. Mack , U. Ritter , M. Schmid , C. Blank , K. Dettmer , P. J. Oefner , P. Hoffmann , S. Walenta , E. K. Geissler , J. Pouyssegur , A. Villunger , A. Steven , B. Seliger , et al., Cell Metab. 2016, 24, 657. 27641098 10.1016/j.cmet.2016.08.011

[15] M. Villa , D. O'Sullivan , E. L. Pearce , Cancer Cell 2021, 39, 460. 33848476 10.1016/j.ccell.2021.03.001

[16] A. Angelin , L. Gil‐de‐Gómez , S. Dahiya , J. Jiao , L. Guo , M. H. Levine , Z. Wang , W. J. Quinn , P. K. Kopinski , L. Wang , T. Akimova , Y. Liu , T. R. Bhatti , R. Han , B. L. Laskin , J. A. Baur , I. A. Blair , D. C. Wallace , W. W. Hancock , U. H. Beier , Cell Metab. 2017, 25, 1282. 28416194 10.1016/j.cmet.2016.12.018 PMC5462872

[17] D. Wang , X. Deng , J. Wang , S. Che , X. Ma , S. Zhang , Q. Dong , C. Huang , J. Chen , C. Shi , M.‐R. Zhang , K. Hu , L. Luo , Z. Xiao , J Control Release 2024, 372, 403. 38914207 10.1016/j.jconrel.2024.06.052

[18] C. Huang , B. Lin , C. Chen , H. Wang , X. Lin , J. Liu , Q. Ren , J. Tao , P. Zhao , Y. Xu , Adv. Mater. 2022, 34, 2207593. 10.1002/adma.202207593 36245299

[19] Y. Guo , Y. u. Fan , Z. Wang , G. Li , M. Zhan , J. Gong , J.‐P. Majoral , X. Shi , M. Shen , Adv. Mater. 2022, 34, 2206861. 10.1002/adma.202206861 36125843

[20] Y. u. Liu , Z. Zhou , J. Hou , W. Xiong , H. Kim , J. Chen , C. Zheng , X. Jiang , J. Yoon , J. Shen , Adv. Mater. 2022, 34, 2206121. 10.1002/adma.202206121 36017886

[21] X. Yi , H. Xie , K. Huang , J. Luo , W. Li , Q. Zeng , F. He , W. Shi , D. Wang , L. Wang , Mater. Today Bio. 2025, 32, 101745. 10.1016/j.mtbio.2025.101745 PMC12019074 40275951

[22] Z. Fan , S. Wu , H. Deng , G. Li , L. Huang , H. Liu , ACS Nano 2024, 18, 12261. 38683132 10.1021/acsnano.4c00844

[23] T. Yang , S. Zhang , H. Yuan , Y. Wang , L. Cai , H. Chen , X. Wang , D. Song , X. Wang , Z. Guo , X. Wang , Angew. Chem., Int. Ed. 2023, 62, 202213337. 10.1002/anie.202213337 36259513

[24] G. Qiu , D. Wang , P. Xie , Z. Li , N. Zhou , X. Zhang , X. Wang , J. Tang , J. Cao , J. Liu , D. Su , Chem. Eng. J. 2024, 495, 153368.

[25] J. Li , S. Ma , Q. Lin , Q. Wang , W. Zhong , C. Wei , J. Liu , J. Chen , D. Wang , W. Tang , T. Luo , Mater Today Bio 2024, 29, 101326. 10.1016/j.mtbio.2024.101326 PMC11600019 39606425

[26] Z. Xiu , Y. Zhu , J. Han , Y. Li , X. Yang , G. Yang , G. Song , S. Li , Y. Li , C. Cheng , Y. Li , J. Fang , X. Li , N. Jin , Front. Pharmacol. 2022, 13, 930958. 35899120 10.3389/fphar.2022.930958 PMC9313605

[27] K. Chen , A. Zhou , X. Zhou , Y. Liu , Y. Xu , X. Ning , Nano Lett. 2023, 23, 3038. 36951267 10.1021/acs.nanolett.3c00434

[28] B. Li , Z. Li , Y. Qian , N. Xiao , C. Fan , Y. Huang , A. Zhou , X. Ning , Nano Lett. 2024, 24, 8107. 38888223 10.1021/acs.nanolett.4c01864

[29] K. Chen , A. Zhou , X. Zhou , J. He , Y. Xu , X. Ning , Sci. Adv. 2024, 10, adk3201. 10.1126/sciadv.adk3201 PMC11006215 38598629

[30] R. Zhang , B. Jiang , K. Fan , L. Gao , X. Yan , Nat. Rev. Bioeng. 2024, 2, 849.

[31] S. Ma , X. Zhang , X. Zhu , K. Yan , Q. Wang , L. Lei , J. Li , J. Guo , W. Tang , J. Liu , J. Cao , D. Wang , T. Luo , J. Nanobiotechnol. 2025, 23, 248. 10.1186/s12951-025-03336-8 PMC11934746 40128784

[32] J. Cravillon , S. Münzer , S.‐J. Lohmeier , A. Feldhoff , K. Huber , M. Wiebcke , Chem. Mater. 2009, 21, 1410.

[33] Y. Sun , K. Ying , J. Sun , L. Qiu , Y. Wang , M. Ji , L. Zhou , J. Chen , Discov Nano 2024, 19, 200. 39661226 10.1186/s11671-024-04168-5 PMC11635067

[34] A. Pal , S. Suresh , A. Khan , L. i. H. Kuo , L. i. T. Chi , A. Ganguly , C.‐Y. Kao , M. K. Sharma , T.‐S. A. Wang , D.‐Y. Kang , Z.‐H. Lin , Sci. Adv. 2025, 11, ads4711. 10.1126/sciadv.ads4711 PMC11708883 39772687

[35] Y. Luo , S. Fan , W. Yu , Z. Wu , D. A. Cullen , C. Liang , J. Shi , C. Su , Adv. Mater. 2018, 30, 1704576, 10.1002/adma.201704576. 29271500

[36] A. Zhou , T. Fang , K. Chen , Y. Xu , Z. Chen , X. Ning , Small 2022, 18, 2106568. 10.1002/smll.202106568 35092152

[37] Y. Zhou , S. Fan , L. Feng , X. Huang , X. Chen , Adv. Mater. 2021, 33, 2104223. 10.1002/adma.202104223 34580933

[38] B. R. Stockwell , Cell 2022, 185, 2401. 35803244 10.1016/j.cell.2022.06.003 PMC9273022

[39] P. Zhao , H. Li , W. Bu , Angew. Chem., Int. Ed. 2023, 62, 202210415. 10.1002/anie.202210415 36650984

[40] X. Fang , H. Ardehali , J. Min , F. Wang , Nat. Rev. Cardiol. 2023, 20, 7. 35788564 10.1038/s41569-022-00735-4 PMC9252571

[41] Y. Li , J. Ma , R. Wang , Y. Luo , S. Zheng , X. Wang , Cell Metab. 2024, 36, 2118. 39111308 10.1016/j.cmet.2024.07.009

[42] P. Koppula , L. Zhuang , B. Gan , Protein Cell 2021, 12, 599. 33000412 10.1007/s13238-020-00789-5 PMC8310547

[43] H.‐F. a. Yan , T. Zou , Q.‐Z. Tuo , S. Xu , H. Li , A. A. Belaidi , P. Lei , Signal. Transduct. Target Ther. 2021, 6, 49. 33536413 10.1038/s41392-020-00428-9 PMC7858612

[44] A. R. Brown , T. Hirschhorn , B. R. Stockwell , Science 2024, 386, 848. 39571009 10.1126/science.adn7030

[45] J. Xie , Y. Yang , Y. Gao , J. He , Molecular Cancer 2023, 22, 46. 36882769 10.1186/s12943-023-01732-y PMC9990368

[46] Z. Guo , D. Chen , L. Yao , Y. Sun , D. Li , J. Le , Y. Dian , F. Zeng , X. Chen , G. Deng , Signal. Transduct. Target Ther. 2025, 10, 149. 40341098 10.1038/s41392-025-02192-0 PMC12062509

[47] C. Mao , M. Wang , L. Zhuang , B. Gan , Protein Cell 2024, 15, 642. 38428031 10.1093/procel/pwae003 PMC11365558

[48] P. Tsvetkov , S. Coy , B. Petrova , M. Dreishpoon , A. Verma , M. Abdusamad , J. Rossen , L. Joesch‐Cohen , R. Humeidi , R. D. Spangler , J. K. Eaton , E. Frenkel , M. Kocak , S. M. Corsello , S. Lutsenko , N. Kanarek , S. Santagata , T. R. Golub , Science 2022, 375, 1254. 35298263 10.1126/science.abf0529 PMC9273333

[49] C. H. Ly , G. S. Lynch , J. G. Ryall , Cell Metab. 2020, 31, 1052. 32433923 10.1016/j.cmet.2020.04.022

[50] M. Li , R. F. Thorne , R. Shi , X. D. Zhang , J. Li , J. Li , Q. Zhang , M. Wu , L. Liu , Adv. Sci. 2021, 8, 2003732, 10.1002/advs.202003732. PMC8188220 34105294

[51] Y. Xu , S. ‐Y. Liu , L. Zeng , H. Ma , Y. Zhang , H. Yang , Y. Liu , S. Fang , J. Zhao , Y. Xu , C. R. A. Jr , Y. He , Z. Dai , Y. Pan , Adv. Mater. 2023, 35, 2300773, 10.1002/adma.202300773. 36987684

[52] Y. Xu , Y. Wu , X. Zheng , D. Wang , H. Ni , W. Chen , K. Wang , Adv. Sci. 2025, 12, 2411378. 10.1002/advs.202411378 PMC11775525 39632613

[53] L. Liu , W. Michowski , A. Kolodziejczyk , P. Sicinski , Nat. Cell Biol. 2019, 21, 1060. 31481793 10.1038/s41556-019-0384-4 PMC7065707

[54] T. Otto , P. Sicinski , Nat. Rev. Cancer 2017, 17, 93. 28127048 10.1038/nrc.2016.138 PMC5345933

[55] M. J. Watson , P. D. A. Vignali , S. J. Mullett , A. E. Overacre‐Delgoffe , R. M. Peralta , S. Grebinoski , A. V. Menk , N. L. Rittenhouse , K. DePeaux , R. D. Whetstone , D. A. A. Vignali , T. W. Hand , A. C. Poholek , B. M. Morrison , J. D. Rothstein , S. G. Wendell , G. M. Delgoffe , Nature 2021, 591, 645. 33589820 10.1038/s41586-020-03045-2 PMC7990682

[56] R. Zappasodi , I. Serganova , I. J. Cohen , M. Maeda , M. Shindo , Y. Senbabaoglu , M. J. Watson , A. Leftin , R. Maniyar , S. Verma , M. Lubin , M. Ko , M. M. Mane , H. Zhong , C. Liu , A. Ghosh , M. Abu‐Akeel , E. Ackerstaff , J. A. Koutcher , P.‐C. Ho , G. M. Delgoffe , R. Blasberg , J. D. Wolchok , T. Merghoub , Nature 2021, 591, 652. 33588426 10.1038/s41586-021-03326-4 PMC8057670

[57] Z. Zhang , C. Zhou , X. Li , S. D. Barnes , S. u. Deng , E. Hoover , C.‐C. Chen , Y. S. Lee , Y. Zhang , C. Wang , L. A. Metang , C. Wu , C. R. Tirado , N. A. Johnson , J. Wongvipat , K. Navrazhina , Z. Cao , D. Choi , C.‐H. Huang , E. Linton , X. Chen , Y. Liang , C. E. Mason , E. de Stanchina , W. Abida , A. Lujambio , S. Li , S. W. Lowe , J. T. Mendell , V. S. Malladi , et al., Cancer Cell 2020, 37, 584. 32220301 10.1016/j.ccell.2020.03.001 PMC7292228

[58] H. Hu , Q. Wang , D. Yu , X. Tao , M. Guo , S. Tian , Q. Zhang , M. Xu , X. Geng , H. Zhang , H. Xu , L. Li , S. Xie , K. Chen , W. Zhu , X. u.‐W. Li , H. Xu , B. o. Li , W. Zhang , S. Liu , Adv. Sci. 2025, 12, 2413122.

[59] V. Lucas , C. Cavadas , C. A. Aveleira , Pharmacol. Rev. 2023, 75, 675. 36732079 10.1124/pharmrev.122.000622

[60] R. Di Micco , V. Krizhanovsky , D. Baker , d'Adda , F. di Fagagna , Nat. Rev. Mol. Cell Biol. 2021, 22, 75. 33328614 10.1038/s41580-020-00314-w PMC8344376

[61] M. Liao , D. Yao , L. Wu , C. Luo , Z. Wang , J. Zhang , B. o. Liu , Acta Pharm. Sin. B 2024, 14, 953. 38487001 10.1016/j.apsb.2023.12.003 PMC10935242

[62] X. Zhong , X. He , Y. Wang , Z. Hu , H. Huang , S. Zhao , P. Wei , D. Li , J. Hematol. Oncol. 2022, 15, 160. 36319992 10.1186/s13045-022-01358-5 PMC9628128

[63] W. Zhang , M. Xia , J. Li , G. Liu , Y. Sun , X. Chen , J. Zhong , Mol. Med. 2025, 31, 146. 40264038 10.1186/s10020-025-01205-6 PMC12016192

