# Supplementary material for: Bimetallic Nanozyme Amplifier for Synergistic Ferroptosis‐Cuproptosis and Metabolic Reprogramming to Reshape Immunosuppressive Tumor Microenvironment
Source: Adv Sci (Weinh). 2025 Oct 15;13(1):e12764. doi: 10.1002/advs.202512764 (PMC12766995; doi:10.1002/advs.202512764)
Supplement: Supplementary file 1 — Supporting Information [file ADVS-13-e12764-s001.docx]

**Supporting Information**

**Bimetallic Nanozyme Amplifier for Synergistic Ferroptosis-Cuproptosis and Metabolic Reprogramming to Reshape Immunosuppressive Tumor Microenvironment**

**Jianzhang Luo ^a, 1^,** **Kunzhao Huang ^a, 1^, Xiaoyuan Yi ^a^, Pei Lu ^a^, Huaying Xie ^a^, Wen Li ^a^, Qingyu Zeng ^a^, Feifei He ^a^,** **Duo Wang ^b,^** ***, Liyan Wang ^a,^ ****

a Digestive Department, The Affiliated Hospital of Guilin Medical University, Lequn road No.15, Xiufeng district, Guilin, 541001, China.

b Center of Interventional Radiology & Vascular Surgery, Department of Radiology, Zhongda Hospital, Medical School, Southeast University, Nanjing, 210009, China.

* Corresponding author: Center of Interventional Radiology & Vascular Surgery, Department of Radiology, Zhongda Hospital, Medical School, Southeast University, Nanjing 210009, China.

** Corresponding author: Digestive Department, The Affiliated Hospital of Guilin Medical University, Lequn road No.15, Xiufeng district, Guilin, 541001, China.

E-mail addresses: wangduo2022@126.com (D Wang), wangliyan@glmc.edu.cn (L. Wang).

^1^ These authors contributed equally to this work^.^

**Materials and methods parts**

**Materials**

DSPE-PEG2000-GA was procured from Xian Qiyue Pharmaceutical Technology Co., Ltd. Cu(NO_3_)_2_·3H_2_O, HAuC1_4_·3H_2_O, 5,5′-dithiobis (2-nitrobenzoic acid, DTNB) were commercially obtained from Macklin Biochemical Co., Ltd (Shanghai, China). [α-cyano-4-hydroxycinnamic acid (CHCA)](https://www.selleck.cn/products/alpha-cyano-4-hydroxycinnamic-acid-alpha-chca.html" \l ":~:text=4-Chloro-%CE%B1-cyanocinnamic acid (%CE%B1-CHCA)%E6%98%AF%E4%B8%80%E7%A7%8D%E7%BB%8F%E5%85%B8%E7%9A%84 monocarboxylate,transporters (MCT) %E6%8A%91%E5%88%B6%E5%89%82%E3%80%82 %E6%AF%94%E8%B5%B7%E5%AF%B9%E5%85%B6%E4%BB%96MCTs%E7%9A%84%E6%95%88%E6%9E%9C%EF%BC%8C%CE%B1-cyano-4-hydroxycinnamate (CHC)%E5%AF%B9MCT1%E5%85%B7%E6%9C%89%E6%9B%B4%E9%AB%98%E7%9A%84%E9%80%89%E6%8B%A9%E6%80%A7%EF%BC%88%E6%98%AF%E5%AF%B9%E5%85%B6%E4%BB%96MCTs%E7%9A%8410%E5%80%8D%EF%BC%89%E3%80%82" \t "https://cn.bing.com/_blank), β-caryophyllene oxide (CO), Zn(NO_3_)_2_∙6H_2_O, CuCl_2_∙2H_2_O, ethylene glycol, 2-Methylimidazole (Hmlm), Polyvinylpyrrolidone (PVP), Glutathione (GSH), rhodamine B (RhB), Anhydrous cobalt chloride (CoCl_2_), PVDF membranes, and Cy5.5 were commercially sourced from Aladdin Biochemical Technology Co. (Shanghai, China). Calcein-AM/PI Cell Viability/Cytotoxicity Assay Kit, Enhanced mitochondrial membrane potential assay kit with JC-1, Glucose content detection kit, GSH/GSSG assay kit, H_2_O_2_ content detection kit, ATP detection kit, MDA detection kit, One Step TUNEL Apoptosis Assay Kit, DNA Damage Comet Assay Kit, 2,7-dichlorodihydrofluorescein (DCFH-DA) probes, PH fluorescent probe (BCECF AM), Annexin V-FITC/PI kit, Cell Cycle and Apoptosis Analysis Kit, Alexa Fluor 488-labeled, Cy3-labeled secondary antibodies, dialysis bag (MW 3.5 kDa cut-off), RIPA lysis buffer, phosphatase inhibitor cocktail, Western blot (WB) transfer buffer, and QuickBlock™ blocking buffer were purchased from Beyotime Biotechnology (Shanghai, China). 3,3′,5,5′-tetramethyl-benzidine (TMB), Cell Copper Content Assay Kit, Hoechst 33342, 2-(4-amidinophenyl)−6-indolecarbamidine dihydrochloride (DAPI), and Red Blood Cell Lysis Buffer were obtained via Solarbio (Beijing, China). Tricolor Prestained Protein Ladder, PAGE gel rapid preparation kit (6%, 10%, 12.5%, and 15%) were commercially procured from Epizyme Biomedical Technology (Shanghai) Co., Ltd. Transwell® with 8.0 µm Pore Polycarbonate Membrane Insert and Corning® Matrigel® Basement Membrane Matrix were obtained from Corning Incorporated Company provided. Zinquin was commercially sourced from Maokang Biotech (Shanghai, China). Cell culture reagents, including FBS, DMEM (standard, high-glucose, and low-glucose variants), RPMI-1640, trypsin-EDTA (0.25%), and penicillin-streptomycin solution, were commercially obtained from Gibco BRL (Gaithersburg, MD, USA). Elesclomol (ES), α-Vitamin E, Oligomycin, 2-deoxy-D-glucose (2-DG), Ferrostatin-1 (Fer-1) and Tetrathiomolybdate (TTM) were procured from MedChemExpress (Monmouth Junction, NJ, USA). FerroOrange was purchased from Dojindo. Cell counting kit-8 (CCK-8) was provided by Zoman Biotechnology (Beijing, China). The cellular iron colorimetric assay kit was obtained from Elabscience Biotechnology Co. ·OH and C11-BODIPY were obtained via ThermoFisher, cat. Triton X-100, Collagenase IV, Hyaluronidase and 4% paraformaldehyde were sourced from Biosharp Biotechnology Co., Ltd (Hefei, China). The following antibodies from BioLegend were used: PerCP/Cyanine5.5-conjugated anti-mouse CD3, APC-labeled anti-mouse CD8, FITC-tagged anti-mouse CD86, PE-conjugated anti-mouse CD80, and APC-coupled anti-mouse CD11c. IL-6 ELISA Kit, TNF-α ELISA Kit, IL-1β ELISA kit, TGF-β ELISA kit, IL-12p70 ELISA kit and IFN-γ ELISA kit were purchased from NeoBioscience Biotechnology Co (Shenzhen, China).

**Synthesis of PEG@AuCZ@CC NPs**

2-Methylimidazole, β-caryophyllene oxide, and [α-cyano-4-hydroxycinnamic acid](https://www.selleck.cn/products/alpha-cyano-4-hydroxycinnamic-acid-alpha-chca.html" \l ":~:text=4-Chloro-%CE%B1-cyanocinnamic acid (%CE%B1-CHCA)%E6%98%AF%E4%B8%80%E7%A7%8D%E7%BB%8F%E5%85%B8%E7%9A%84 monocarboxylate,transporters (MCT) %E6%8A%91%E5%88%B6%E5%89%82%E3%80%82 %E6%AF%94%E8%B5%B7%E5%AF%B9%E5%85%B6%E4%BB%96MCTs%E7%9A%84%E6%95%88%E6%9E%9C%EF%BC%8C%CE%B1-cyano-4-hydroxycinnamate (CHC)%E5%AF%B9MCT1%E5%85%B7%E6%9C%89%E6%9B%B4%E9%AB%98%E7%9A%84%E9%80%89%E6%8B%A9%E6%80%A7%EF%BC%88%E6%98%AF%E5%AF%B9%E5%85%B6%E4%BB%96MCTs%E7%9A%8410%E5%80%8D%EF%BC%89%E3%80%82" \t "https://cn.bing.com/_blank) were dissolved in methanol, then add the aqueous solution of Zn(NO_3_)_2_∙6H_2_O and Cu(NO_3_)_2_·3H_2_O under stirring. After stirring at 25^°^C for 10 minutes, centrifuge the mixture (12,000 rpm, 5 min) to separate the product and methanol washing several times. Subsequently, add solution of HAuC1_4_·3H_2_O in the dark, stir for one hour under ice bath. Then add Sodium borohydride solution, followed by stirring for 3 hours. After completion, centrifuge the reaction mixture (12,000 rpm, 5 min) and wash the precipitate with methanol three times to remove unreacted impurities. Finally, lyophilize the product under vacuum, weigh, and store it.

**Characterizations**

The morphologies of ZIF-8, AuCZ@CC, and PEG@AuCZ@CC nanomaterials were observed using TESCAN CLARA scanning electron microscopy (SEM) and FEI Tecnai G2 F30 transmission electron microscopy (TEM). The elemental composition of the nanomaterials was further evaluated using energy dispersive spectrum (EDS) and elemental mapping (Mapping). Molecular functional group analysis was conducted using FTIR spectroscopy. The crystal phases of ZIF-8, AuCZ@CC, and PEG@AuCZ@CC were characterized by X-ray powder diffraction (XRD, Bruker D8 Advance) with a Cu target, operated at 40 kV and 40 mA, and scanned over a 2θ range of 5-50°. Then, the Malvern Zetasizer Nano-ZS90 system was employed for DLS measurements to determine particle size and zeta potential of ZIF-8, AuCZ@CC and PEG@AuCZ@CC NPs diluted with ultrapure water. To evaluate the colloidal stability of PEG@AuCZ@CC nanoparticles, various solution systems were employed to mimic physiological environments, including ultrapure water and DMEM supplemented with 10% FBS. PEG@AuCZ@CC NPs were dispersed in these solutions, and its size distribution and stability were monitored over time using DLS.

**Detection of CHCA and CO**

CHCA and CO exhibit characteristic absorption peak 324 nm and 208 nm. Then, the UV-vis absorption spectra of CO, CHCA, ZIF-8, AuCZ@CC, and PEG@AuCZ@CC were recorded using a UV-vis spectrophotometer (UV-1800).

**Drug load calculation and release behaviors of PEG@AuCZ@CC NPs**

First, different concentrations of CO and CHCA solutions were prepared, and based on the optical density measurements across varying concentrations, standard curves were plotted. The supernatants obtained during nanoparticle preparation (via centrifugation) were collected, and the UV-vis spectrophotometric analysis was performed at wavelengths of 208 nm and 324 nm, with drug quantification achieved through standard curve calibration. Then, 1 mL PEG@AuCZ@CC NPs (1 mg/mL) were added to dialysis membrane (MW cutoff: 3500 Da). The dialysis membrane was submerged in phosphate buffer saline (PBS, 10 mM) at varying pH levels (7.4, 6.4, and 5.4) with shaking at 100 rpm under room temperature. At preset times (0, 1, 2, 4, 6, 12, 24 and 48 hours), the PBS was removed and replenished with new PBS. Absorbance measurements of the PBS eluate were obtained using UV-vis spectroscopy, with subsequent quantification of CO and CHCA concentrations via standard curve regression analysis.

**The ability of the PEG@AuCZ@CC NPs to consume GSH**

The GSH consumption ability of PEG@AuCZ@CC NPs was evaluated by colorimetric method using DTNB as a chromogenic substrate. First, 600 μg of ZIF-8, AuCZ@CC, and PEG@AuCZ@CC NPs were dispersed in PH 6.4, 0.1M PBS, 30 μL of 10 mM glutathione (GSH) solution was added, with a total volume of 3mL. Samples were gently shaken (100 rpm) at physiological temperature (37°C) for 60 minutes. Subsequently, we take 30 μL, 2 mg/mL of DTNB added to each sample and continue to shake for 5 minutes. Spectrophotometric analysis was conducted immediately following sample preparation. Spectral scanning was performed across 300-600 nm, with specific absorbance measurements recorded at 412 nm. At the same time, different concentrations of PEG@AuCZ@CC NPs (0-250 μg/mL in 50 μg/mL increments) were subjected to identical treatment conditions, and the 412 nm absorbance were measured. Finally, GSH concentrations across various samples and concentration gradients were quantified using a commercial GSH/GSSG assay kit.

**GOx-like activity**

Initially, an exact quantity of anhydrous D-glucose was solubilized in phosphate-buffered saline. Then, equal amounts of ZIF-8 and PEG@AuCZ@CC NPs were introduced into the glucose solution. To prevent atmospheric interference, the solution surface was overlaid with a layer of corn oil, and gently shake at 37^°^C for one hour. The experimental evaluations included: Glucose Consumption Analysis: monitoring variations in the characteristic UV absorption peak at 630 nm and using a glucose assay kit to quantitative assessment of glucose concentration changes. Dissolved Oxygen Monitoring: continuous measurement of oxygen concentration for 1 hour using the dissolved oxygen meter. The pH Value Determination: pH measurement using a pH meter with comparative analysis across groups. Hydrogen Peroxide Quantification: Spectrophotometric detection at 415 nm through the formation of yellow peroxin-titanium complexes via reaction with titanium sulfate, and precise concentration determination using the hydrogen peroxide assay kit.

**Cell lines and cell culture**

The Hepa1-6 and Hepa1-6-Luc1 murine hepatoma cell lines were commercially acquired from Zhejiang Meisen Biotechnology. Normal hepatocyte lines (AML-12, THLE-2) and macrophage cells (RAW264.7) were sourced from American Type Culture Collection (ATCC). All cells were authenticated by short tandem repeat (STR) profiling. The primary dendritic cells (DCs) isolated from the femurs of C57BL/6 mice. All hepatocyte-derived cell lines (Hepa1-6, Hepa1-6-Luc1, AML-12, THLE-2) were maintained in DMEM (Gibco) containing 10% fetal bovine serum (FBS, Gibco) and 1% penicillin-streptomycin (penicillin and streptomycin). Immune cells (DCs and RAW264.7) were propagated in RPMI-1640 medium with identical serum and antibiotic supplementation. In specified assays, hypoxia was simulated in Hepa1-6 cells through CoCl₂ treatment (300 μM final concentration). All cellular experiments were performed using incubator set to maintain 37°C with 5% carbon dioxide and saturated humidity.

**Cellular uptake assessment**

To comparative analysis of cellular uptake pathways between AuCZ@CC and PEG@AuCZ@CC NPs, these two nanostructures were modified with rhodamine B (RhB). Hepa1-6 cells were seeded on coverslips in 24-well plates at a density of 3×10⁴ cells/well and maintained at 37°C for 24 hours. Cells were subsequently cultured with RhB-labeled AuCZ@CC and PEG@AuCZ@CC at intervals (0, 30, 60, 120, 240 min), respectively. After incubation, the medium was removed, and one part of the cells were washed three times with PBS before being incubated with fresh medium. Following 15-minute Hoechst 33342 nuclear staining, cell samples were visualized via confocal microscopy (CLSM). In addition, another part of the cells was co-incubated with RhB-labeled AuCZ@CC and PEG@AuCZ@CC. Subsequently, in each group, cells were trypsinized, and ultimately assessed using a Beckman Coulter (USA) flow cytometer.

**Determination of Cu concentration in cells**

To measure intracellular Cu levels, Hepa1-6 cells (1×10⁶ cells/well) were plated in 6-well plates and cultured overnight at 37°C. Subsequently, the cells were subjected to the following procedures for a period of 6 hours: (1) control group, (2) CO, (3) CO+CHCA, (4) CZ@CC, (5) AuCZ@CC, and (6) PEG@AuCZ@CC. After treatment, intracellular copper concentrations were quantified with a commercial Copper Assay Kit.

**Determination of intracellular Fe^2+^ content**

For quantification of intracellular Fe^2+^ concentrations, FerroOrange probe was used. Hepa1-6 cells (2×10⁵ cells/dish) were placed in 2.0 cm confocal culture dishes for incubation, and the cells were incubated for 24 hours. Cells were divided into six groups (control, CO, CO+CHCA, CZ@CC, AuCZ@CC and PEG@AuCZ@CC) and exposed to varying treatments for 24 h. Cells were stained with DAPI for nuclear and stained with FerroOrange fluorescent probe for intracellular Fe^2+^. Finally, cells were imaged by fluorescence microscopy. For quantitative analysis, Hepa1-6 cells (1×10^5^ cells/well) were seeded in 6-well plate, and incubated for one day. After consistent treatment protocols, cellular samples were collected and analyzed with the Ferrous Iron Colorimetric Assay Kit.

**Cytotoxicity assay**

AML-12, THLE-2, and Hepa1-6 cells were plated in 96-well culture plates at a density of 5×10³ cells/well and maintained at 37°C. Then, different concentrations (0-120 µg/mL) of CHCA, CO, CO+CHCA, CZ@CC, AuCZ@CC and PEG@AuCZ@CC were incubated for 24 hours. Following incubation, 10 μL of CCK-8 reagent was introduced into every well and left to incubate for a period of 1.5 hours. Cell viability was quantified by measuring absorbance at 450 nm with a Tecan microplate reader (Switzerland).

**Intracellular/Extracellular lactate content assay**

For adherent cells after treatment, lysis buffer was added at a ratio of 100-200 µL per 1×10^6^ cells. To achieve thorough cellular disruption, samples were maintained at 0-4°C for 5-10 minutes. Centrifuge (12,000 rpm, 5 min, 4^°^C), and the upper layers were collected for lactate quantification using L-lactate dehydrogenase assay kit.

**Intracellular pH determination**

In this study, BCECF-AM (Beyotime) was used as a fluorescent indicator to measure intracellular pH. Hepa1-6 cell suspensions were plated in 24-well culture dishes at a density of 3×10⁵ cells per well. After cell attachment, the cells were treated with different groups (Control, CO, CO+CHCA, CZ@CC, AuCZ@CC, and PEG@AuCZ@CC) for one day. Later, cells were incubated with 5 µM BCECF-AM fluorescent dye for 30 minutes at 37°C under light-protected conditions, followed by nuclear staining with 500 µL of 1×Hoechst 33342 for 10 min. Finally, fluorescent signals were captured utilizing an inverted epifluorescence microscopy platform.

**Assessment of hydrogen peroxide generation capacity**

For the experiment, 6-well plates were inoculated with Hepa1-6 cells (1×10⁵ cells/well) and maintained in culture for 24 hours. Six groups were established (Control, CO, CO+CHCA, CZ@CC, AuCZ@CC, and PEG@AuCZ@CC), and the cells were treated for 24 hours, respectively. The cells were harvested and transferred to centrifuge tubes, and lysis buffer was added at 100-200 mL/million cells. Samples were centrifuged at 12k rpm (4°C) for 3-5 min. The supernatant was collected, and use the H_2_O_2_ detection kit to detect the content of H_2_O_2_ in each group.

**Intracellular ROS detection**

The Fenton reaction generates abundant ROS. The fluorescent probe DCFH-DA was employed to track reactive oxygen species generation. Hepa1-6 cells were plated in 24-well culture dishes at 4×10⁴ cells per well and maintained for 24 h prior to an 8-hour treatment period. Subsequently, the culture medium was replaced with serum-free medium. The DCFH-DA probe was applied to treated cells for 20 min, followed by triple PBS washes and FCM quantification. Intracellular ROS evaluation was performed by fluorescence microscopy following a 10-minute staining with Hoechst 33,342.

**Intracellular ·OH determination**

Hepa1-6 cells were seeded in 12-well plates (1×10^5^ cells/well) and incubated for one day. Then, the cells were exposed to different groups (Control, CO, CO+CHCA, CZ@CC, AuCZ@CC, and PEG@AuCZ@CC) for 24 hours. Following medium replacement with serum-free solution, cells were labeled with ·OH detection probe for half an hour. Subsequent to PBS washes, nuclear staining was performed using 500 μL of 1× Hoechst 33342 (15 min) for microscopic visualization.

**GSH and GSSG content**

To quantify intracellular glutathione, Hepa1-6 cells were plated in 24-well plates (1×10⁵ cells/mL) overnight. Following treatment groups (Control, CO, CO+CHCA, CZ@CC, AuCZ@CC, PEG@AuCZ@CC), cells underwent triple PBS washing and serum-free medium replacement. ThiolTracker™ Violet staining (30 min, dark) preceded fluorescence microscopy analysis. Parallel experiments in 6-well plates (2×10⁵ cells/well) employed a commercial GSH assay kit after 10 h treatments and PBS washing.

**Lipid oxidation determination**

Hepa1-6 cells were plated in 12-well culture dishes at a density of 1×10⁵ cells/well to ensure proper adhesion. After 24 hours of incubation with drugs (control, CO, CO+CHCA, CZ@CC, AuCZ@CC, and PEG@AuCZ@CC). Following triple washing with phosphate buffer saline, samples were incubated with the lipid peroxidation probe C11-BODIPY for half an hour under the protection of light. The final step was to image the cells using a fluorescence microscope.

**Assessment of oxidative stress through MDA content analysis**

For experimental setup, 6-well plates were inoculated with Hepa1-6 cells at 1×10⁵ cells/mL concentration followed by overnight incubation. Six groups (control, CO, CO+CHCA, CZ@CC, AuCZ@CC, and PEG@AuCZ@CC) were set up. After 24 hours treatments, cells were washed multiple times with phosphate buffer saline. Then, cells were collected from each of the groups, and lipid oxidation (MDA) content of each group was detected by MDA assay kit.

**Measurement of intracellular** **glucose content**

Six experimental groups of Hepa1-6 cells were cultured in 6-well plates at 2×10⁵ cells/well density (Cu/ZIF-8, Cu/ZIF-8@CHCA, Au/Cu/ZIF-8, Au/Cu/ZIF-8@CHCA, PEG-Au/Cu/ZIF-8, and PEG-Au/Cu/ZIF-8@CHCA). After treatments (24 hours), PBS rinsing was performed three times to ensure complete removal of residual media. To each well, lysis buffer (200 μL) was added. Following cell lysis, centrifugation was performed to separate the supernatant, which was then analyzed for glucose content using a commercial assay kit.

**JC-1-based evaluation of mitochondrial depolarization**

Mitochondrial membrane potential assessment was performed by plating Hepa1-6 cells in 24-well plates (4×10⁴ cells/well) overnight. Following 24-hour treatment (Control, CO, CO+CHCA, CZ@CC, AuCZ@CC, PEG@AuCZ@CC), cells underwent PBS washing before incubation with 1 mL JC-1 solution (30 min). After removing excess dye through two washes, 1 mL serum-free medium was added. Finally, an inverted epifluorescence microscope was employed for dynamic observation of mitochondria in both J-monomers and J-aggregates.

**Western blot assay**

For experimental preparations, 6-well plates were inoculated with Hepa1-6 cells (2×10⁵ cells/well) and cultured overnight to allow adherence. Six groups (control, CO, CO CHCA, CZ@CC, AuCZ@CC and PEG@AuCZ@CC) were set up and co-incubated with Hepa1-6 cells for 24 hours. Following two PBS washes, cellular proteins were extracted and lysate protein concentrations were quantified and normalized using a NanoDrop™ One spectrophotometer (Thermo Scientific). For tissue samples, 1 mL of red blood cell lysis buffer was added to remove excess red blood cells, followed by RIPA lysis buffer containing PMSF. After homogenization and centrifugation, the supernatant was obtained and the concentration of the protein was measured and weighed. Both cell samples and tissue samples were isolated by SDS-PAGE (6%, 10%, 12.5%, and 15%) and transferred to PVDF membranes. Following blocking, PVDF membranes were probed with specific primary antibodies followed by DyLight™ 680-conjugated secondary antibodies (Cell Signaling Technology). Finally, the expression of the target proteins was visualized using an Odyssey DLx near-infrared imaging system (LI-COR Biosciences) for dual-color detection, and semi-quantitatively analyzed using Image-J software (version 2.0.0).

**Live/Dead cell staining assay**

Cytotoxicity was assessed using a live/dead viability assay. Hepa1-6 cells (1×10⁵ cells/well) were plated in 6-well plates and cultured for one day to ensure adhesion. Following two PBS washes, cells were divided into six experimental groups for nanoparticles treatment: Control, CO, CO+CHCA, CZ@CC, AuCZ@CC, and PEG@AuCZ@CC. After 24 hours, removed the medium and washed cells. Then, these cells were stained by sequential addition of Calcein-AM and propidium iodide (1 µL each), followed by 30 min incubation. An inverted fluorescence microscope and Image-J software (version 2.0.0) were used to visualize and quantify live and dead cells.

**Detection of cell apoptosis**

Hepa1-6 cell suspensions were plated in 6-well culture dishes at a density of 1×10⁵ cells per well and maintained for one day under standard conditions. These cells were cultured with six groups (control, CO, CO+CHCA, CZ@CC, AuCZ@CC and PEG@AuCZ@CC) for an additional 24 hours. Then, we collected the medium for each group and harvest the cells with trypsin (without EDTA). In the dark, cell samples were sequentially stained with FITC (10 min) and PI (5 min) for apoptosis analysis. Cell apoptosis rates were determined by flow cytometric analysis.

**EdU cell proliferation experiment**

Prior to treatment, Hepa1-6 cells were seeded at 2×10⁴ cells/mL concentration in 48-well plates. These cells with different treatments (Control, CO, CO+CHCA, CZ@CC, AuCZ@CC, and PEG@AuCZ@CC) for 12 hours after cell recovery to normal status. Then, cell proliferation was assessed using an EdU kit according to the manufacturer's instructions.

**Colony formation assay**

First, Hepa1-6 cells were plated in 6-well culture dishes (1×10³ cells/well) and allowed to adhere. Then, they were subjected to different types of treatment and cultured (medium changes every 3 days) until colony formation occurred. When colony formation, they were first fixed in 4% PFA, then subjected to crystal violet staining (0.5%). Subsequent washing, drying, and imaging procedures preceded quantitative analysis using Image-J software.

**Migration assay**

Following 12-hour serum starvation in DMEM, Hepa1-6 cell migration was assessed using 8 μm pore Transwell inserts. Cells (2×10⁴/well) were suspended in serum-free medium in upper chambers, while complete medium filled lower chambers. After adhesion, six treatment groups (Control, CO, CO+CHCA, CZ@CC, AuCZ@CC, and PEG@AuCZ@CC) were applied to upper chambers for 24 h. Fixed cells (4% PFA) were stained with 0.5% crystal violet (30 min), imaged by brightfield microscopy, and quantified using Image-J software.

**Invasion assay**

Based on the migration assay, the Basement Membrane Matrix was pre-coated in the upper chamber of the transwell, the rest of the operations were consistent with the migration assay.

**Wound-healing assay**

Wound healing capacity was assessed by generating uniform scratches in confluent Hepa1-6 monolayers (6-well plates) using sterile pipette tips (10 μL). Following PBS washes to clear debris, serum-free DMEM containing experimental compounds was applied for 24-hour incubation (Control, CO, CO+CHCA, CZ@CC, AuCZ@CC, and PEG@AuCZ@CC). Microscope bright field images were captured at 0h, 12h, and 24h. The gap area was measured and analyzed using Image-J software.

**Comet assay**

For this experiment, the frosted glass slides, slides, coverslips, etc., to be used were soaked in 75% ethanol and wiped clean for backup. (1) Gel preparation First layer of gel preparation: Preheat the frosted slides, drop 200 ml of molten 1.5% NMA, cover with a coverslip, and place the slides in a 4^°^C refrigerator for 10 minutes; Second layer of gel preparation: Take out the first layer of gel, remove the coverslip, mix the cell suspension with 1% LMA at a ratio of 1:8, drop 100 ml of the mixture onto the first layer of gel, cover with a coverslip, and place the slides in a 4^°^C refrigerator for 10 minutes. (2) Lysis Prepare the lysis solution, place the slides horizontally into the cell lysis solution, and lyse for 1 hour. (3) Unwinding Prepare the electrophoresis buffer, take the slides out of the lysis solution, place them in a horizontal electrophoresis tank, add the electrophoresis buffer, ensuring the liquid level is about 0.25 cm above the gel surface of the slides, and unwind for 20 minutes. (4) Electrophoresis Adjust the electrophoresis apparatus to stabilize the voltage at 20 V and the current intensity at 200 mA, and perform electrophoresis for 30 minutes. (5) Rinsing After electrophoresis stops, rinse the slides with a 0.4 mol/L Tris-HCl neutral buffer for about 10 minutes each time. (6) Staining Prepare the staining solution with 20 μg/ml ethidium bromide (EB), drop 50 ml onto each slide, cover with a coverslip, and observe the results under a fluorescence microscope. Use Image-J software and the OpenComet plugin for result extraction and analysis (the main focus indicators are Tail DNA %).

**Sphere** **formation assay**

To better simulate the effect of drugs on tumors in vivo, Hepa1-6 cells were plated in 96-well U-bottom ultra-low attachment plates and maintained until spheroids achieved a mean diameter of 100 μm. Uniformly sized tumor spheres were then transferred to a 6-well plate and exposed to various experimental conditions (Control, CO, CO+CHCA, CZ@CC, AuCZ@CC, and PEG@AuCZ@CC). The diameter and number of tumor spheroids were continuously recorded over 4 days.

**Immunofluorescence staining**

For the co-culture experiment, Hepa1-6 and RAW264.7 cells were plated in confocal dishes (3×10⁵ cells/dish) and grown for one day. Following adhesion, cells received various treatments (Control, CO, CO+CHCA, CZ@CC, AuCZ@CC, PEG@AuCZ@CC) for 12 h. After PBS washing (two times) and 4% PFA fixation (15 min), permeabilization was performed using 0.5% Triton X-100/PBS (20 min, RT). Cells were blocked with QuickBlock™ (Beyotime, 15 min), then incubated with primary antibodies (1:300, 12 h, 4°C) followed by Alexa Fluor 488 or Cy3-conjugated secondaries, and nuclear staining used Hoechst 33342 before CLSM imaging and Image-J software quantification.

**ATP level determination**

Hepa1-6 cells plated in 6-well culture dishes were exposed to experimental treatments for 24 h, and the ATP quantification using an ATP assay kit following standard protocols. Meanwhile, sample protein content was quantified. Finally, the ATP content per milligram of protein in each cell sample and medium sample was calculated.

**Co-incubation and maturation of DC cells**

A Transwell co-culture system was established with bone marrow-derived dendritic cells (DCs) in the lower chamber and Hepa1-6 cells in the upper chamber, followed by 16-hour incubation. After 8 hours of treatments (Control, CO, CO+CHCA, CZ@CC, AuCZ@CC, PEG@AuCZ@CC) of Hepa1-6 cells, DCs were then harvested and labeled with anti-CD11c, anti-CD80, and anti-CD86 antibodies for flow cytometric analysis of CD11c+CD80+CD86+ populations.

**Co-incubation and differentiation of RAW264.7 cells**

RAW264.7 cells were seeded in Transwell culture plate, with Hepa1-6 cells in the upper chamber and RAW264.7 cells in the lower chamber, followed by overnight incubation. Then, Hepa1-6 cells were treated with the following groups for 8 hours: Control, CO, CO+CHCA, CZ@CC, AuCZ@CC, and PEG@AuCZ@CC. After treatment, Hepa1-6 cells were co-incubated with RAW264.7 cells for 24 hours. Finally, RAW264.7 cells were collected and stained with anti-CD45, anti-F480, anti-CD206, and anti-CD86 antibodies. The percentages of CD206+CD86- and CD206-CD86+ cells were analyzed by FCM.

**Hemolytic test**

The blood compatibility and biosafety of PEG@AuCZ@CC NPs were assessed by examining its compatibility with red blood cells (RBC) at different concentrations. Three experimental sets were prepared: negative controls (800 μL PBS + 200 μL RBC), positive controls (800 μL ultrapure water + 200 μL RBC), and experimental groups (800 μL different concentrations of PEG@AuCZ@CC NPs or different drugs + 200 μL RBC). When hemolysis occurred in positive control group, the supernatant of experimental groups was collected by centrifugation (2000 rpm, 5 min). Finally, absorbance measurements at 540 nm wavelength were obtained and used to calculate erythrocyte lysis percentages. In addition, morphological changes in RBC were observed under a 20X microscope.

**Construction of tumor models in vivo**

Four-week-old C57BL/6 mice (Experimental Animal Center, Guilin Medical University) were utilized following ethical approval (GLMC202303112) from the Ethics Committee of the Animal Laboratory of Guilin Medical University. All animal procedures strictly adhered to China's national laboratory animal welfare standards (GB/T35892-2018) and the Ministry of Science and Technology's ethical guidelines for humane treatment of research animals. These healthy mice were fed in controlled environmental conditions (23°C, 50% relative humidity). All animal experiments complied with China's State Council regulations on laboratory animal management. Hepa1-6(Luc1) cells were suspended at 8×10⁶ cells in 100 μL PBS and inoculated subcutaneously into mice. Treatment commenced when tumor volumes reached 0.8 cm in maximal diameter.

**Bioluminescence imaging**

The Cy5.5 (1 mg) was completely dissolved in PBS (1 mL), add AuCZ@CC NPs (1 mg) and stir overnight at light blocking. The precipitate was collected via centrifugation (10000 rpm, 10 min) to obtain Cy5.5-labeled AuCZ@CC NPs. Then, PEG-GA was coated onto the Cy5.5-labeled AuCZ@CC NPs to prepare Cy5.5-labeled PEG@AuCZ@CC NPs. After the successful establishment of the subcutaneous tumor model, tumor-bearing mice were intravenously injected via the tail vein with AuCZ@CC/Cy5.5 and PEG@AuCZ@CC/Cy5.5 (1 mg/mL, 100 μL). At different time points (0, 0.5, 1, 2, 4, 8, 12, and 24 hours), the mice were anesthetized and imaged using a small animal in vivo imaging system (PerkinElmer IVIS instrument, Waltham, MA, USA). For in vitro biodistribution assessment, sacrificed animals underwent tumor and organ collection (heart, liver, spleen, lungs, kidneys) for subsequent imaging.

**Biological safety evaluation**

Biosafety assessment was conducted using healthy C57BL/6 mice (4-week-old) to evaluate treatment toxicity profiles in vivo. Mice were injected intravenously with different drugs three times in total (once dose of 5 mg/kg). After 2 weeks, mice were sacrificed. Then, histopathological examination was conducted on vital organs (heart, liver, spleen, lungs, kidneys) from all experimental groups using hematoxylin and eosin staining. Concurrently, collected blood specimens were analyzed for biochemical indices (WBC, RBC, PLT, HGB, MCV, ALT, AST, TBIL, CREA, and UA).

**In vivo treatment against tumor**

After confirming the biosafety of different treatments. Four-week-old C57BL/6 mice (n=24) were randomly allocated to six experimental groups: G1: PBS, G2: CO, G3: CO+CHCA, G4: CZ@CC, G5: AuCZ@CC, and G6: PEG@AuCZ@CC. Mice were injected via tail vein three times (days 1, 4 and 7) to suppress tumors, with the experimental period was 16 days. During treatment, each mouse was continuously monitored for tumor size and body weight. At the end of treatment, tumor tissues and vital organs were harvested for subsequent analysis. Briefly, tumors were stained with immunohistochemistry and immunofluorescence, including H&E staining, TUNEL, Ki67, PTGS2, GPX4, DLAT, CRT, HMGB1, and CD8^+^/CD4^+^, respectively, and then visualized under an optical or fluorescence inverted microscope.

**Cytokine detection**

After different treatments, orbital venous blood samples were obtained from mice and processed to isolate serum. Cytokine concentrations (IL-1α, IL-12p70, IL-6, IL-10, TNF-α, TGF-β, IFN-γ) were quantified using ELISA kits following the manufacturer's protocols.

**Flow cytometric analysis**

Following treatment completion, inguinal lymph nodes were aseptically harvested into RPMI-1640 medium (1 mL). Tissues were mechanically dissociated, filtered through 40 μm meshes, and centrifuged (800 rpm, 5 min). Cell suspensions were then treated with 5% BSA (15 min) to prevent nonspecific antibody binding. Subsequently, the cells were stained in the dark for 30 min with antibodies such as CD11c, CD80, and CD86. The activation status of dendritic cells (DCs) was evaluated by flow cytometry analysis. Similarly, tumors collected from the mice were treated with Collagenase IV and Hyaluronidase to generate single-cell suspensions. The collected cells were further stained with corresponding fluorophore-labeled antibodies, including CD45, CD11b, Gr-1, F4/80, CD206, CD86, FITC, CD3, CD4, CD80, and Foxp3. Cell populations were characterized by flow cytometric analysis following standardized protocols, with subsequent data processing conducted in FlowJo software.

**Statistical analysis.**

All values are expressed as mean ± standard deviation (SD), statistical analyses were conducted using GraphPad Prism software (version 10.0). The significant difference of the experimental data was analyzed via One-way analysis of variance (ANOVA) or t-tests. Difference was considered to be significant if P < 0.05 (*p < 0.05, **p < 0.01, ***p < 0.001, ****p < 0.0001, “ns” indicates no significant).

**Figures parts**


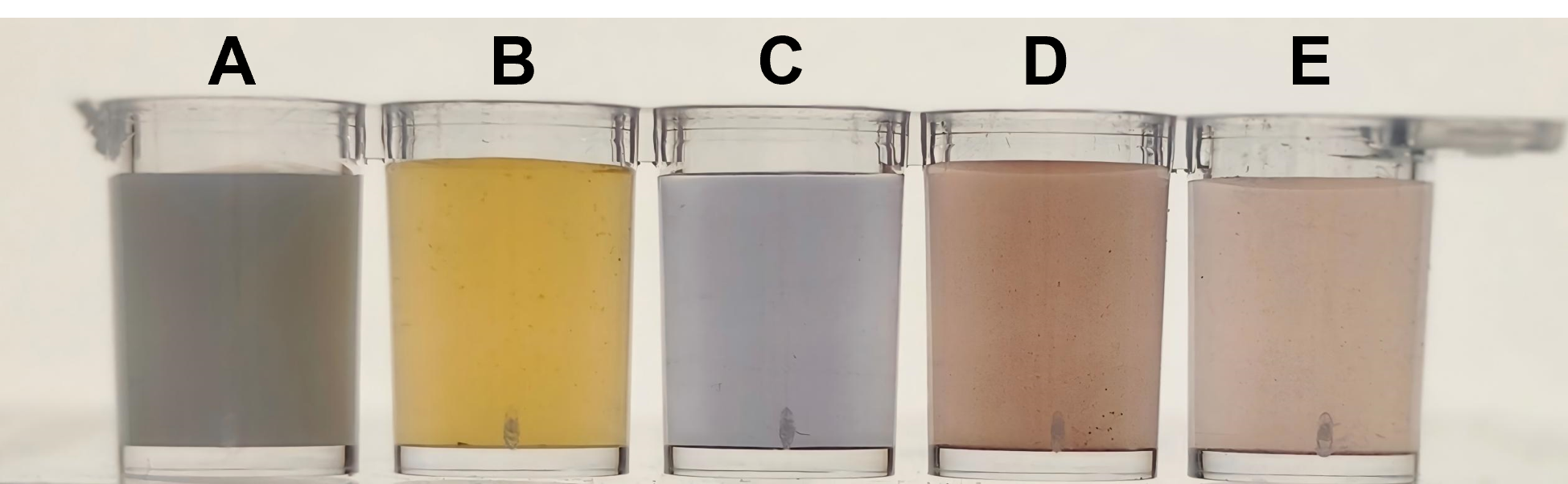


**Figure S1.** Aqueous solutions of samples at each synthesis stage. (A) ZIF-8, (B) Cu/ZIF-8, (C) CZ@CC, (D) AuCZ@CC, and (E) PEG@AuCZ@CC.


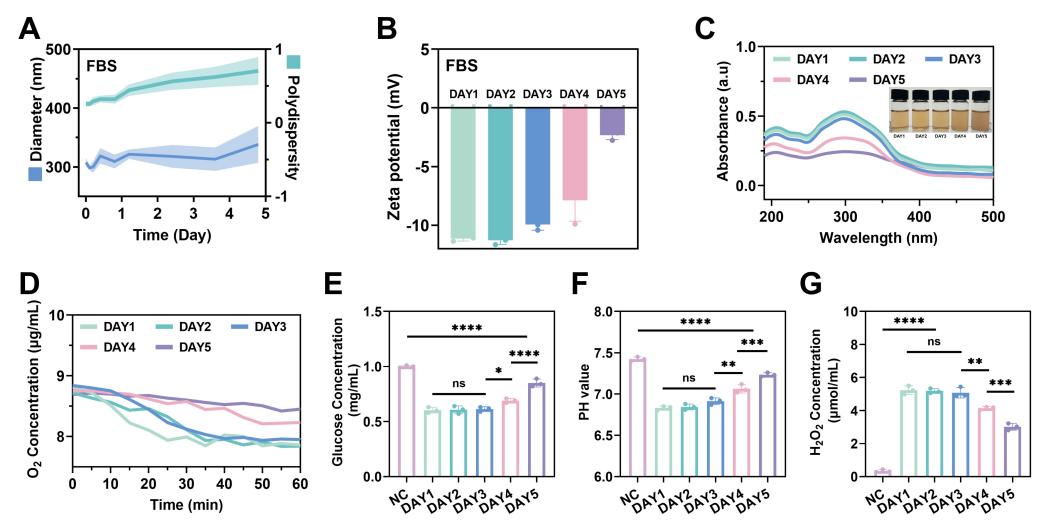


**Figure S2.** The **(A)** DLS (size and PDI), **(B)** zeta potential, **(C)** changes in UV absorption and color of the solution, and **(D-G)** changes in catalytic activity of PEG@AuCZ@CC in serum for 5 days. Data are expressed as mean ± standard deviation (SD, n=3). One-way Anova or t-test was used to analyze statistical differences between groups. *P < 0.05, **P < 0.01, ***P < 0.001, ****p < 0.0001. "ns" denotes no significant difference.


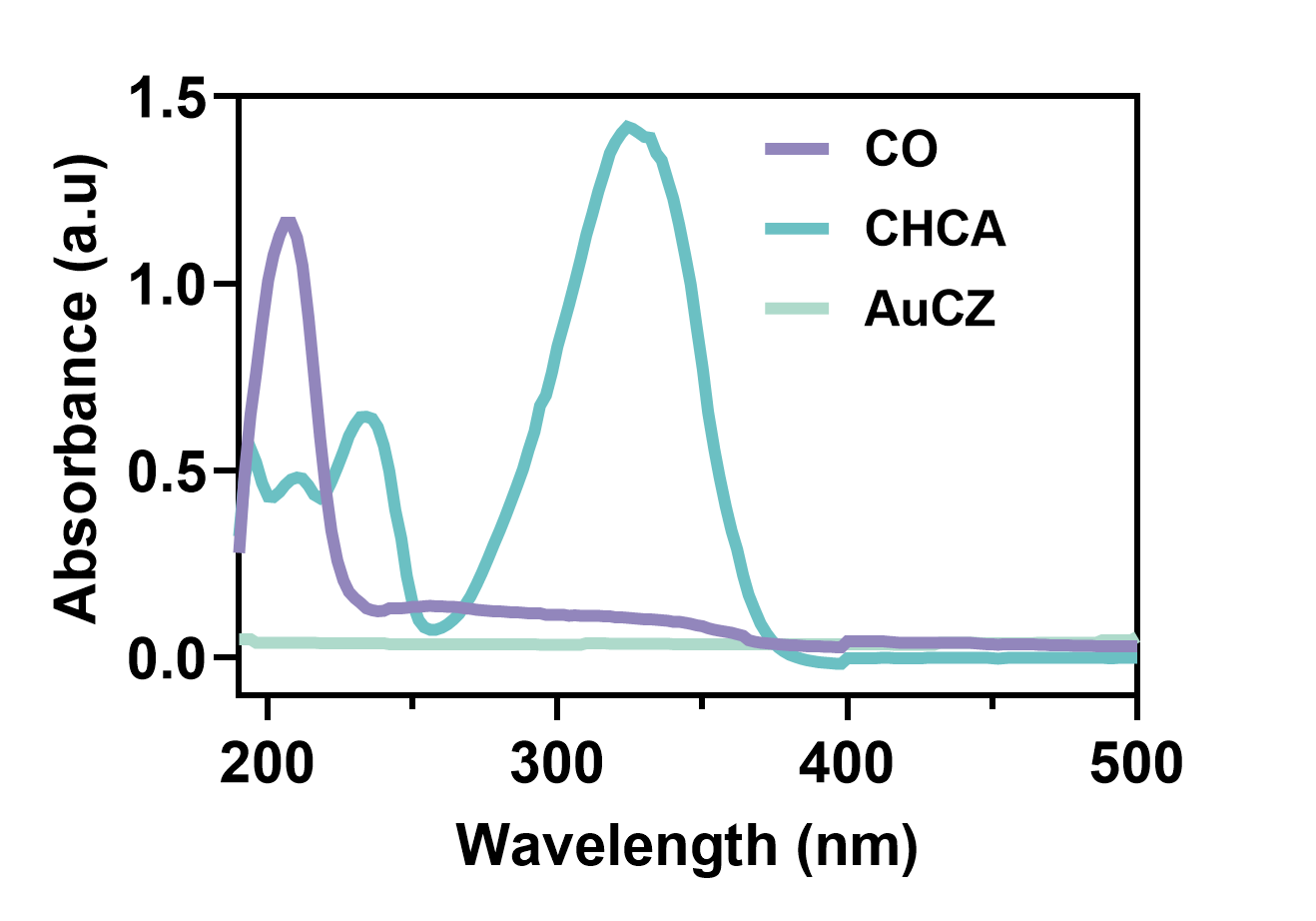


**Figure S3.** UV-vis absorption spectra of CO, CHCA, and AuCZ.


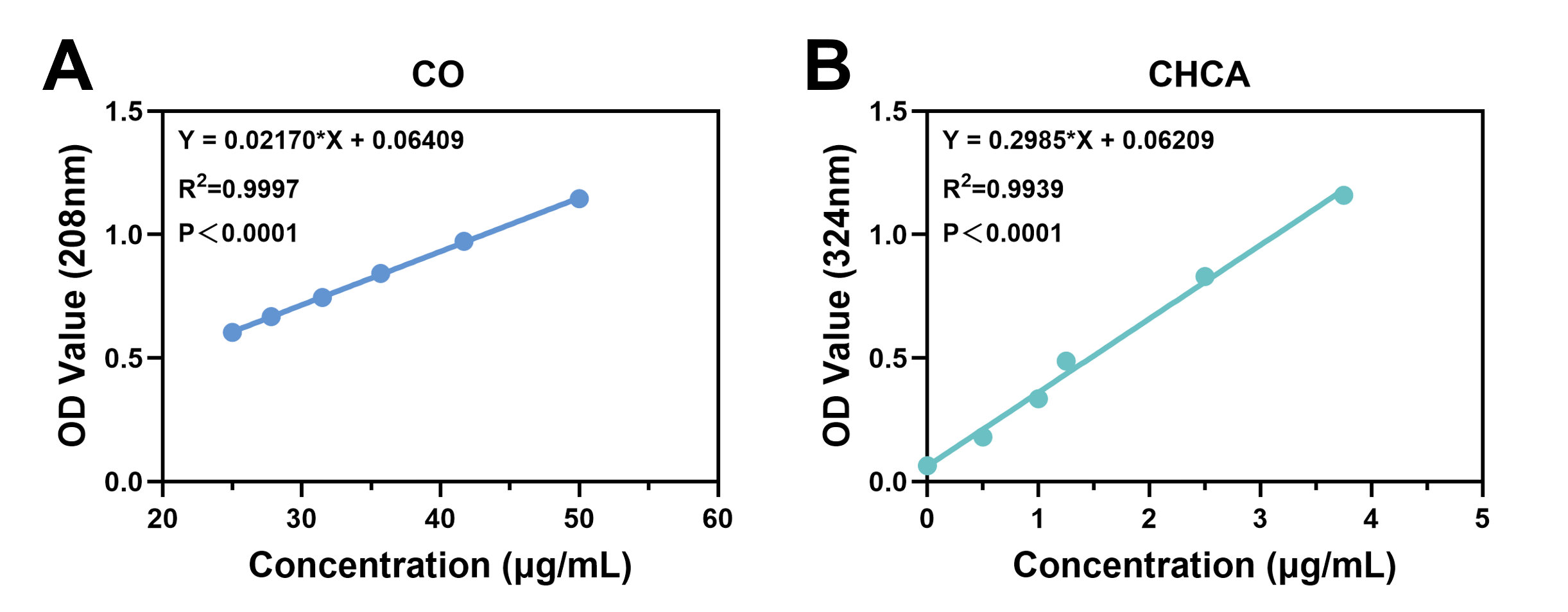


**Figure S4.** Fitting curve of absorbance of (A) CO and (B) CHCA versus doses.


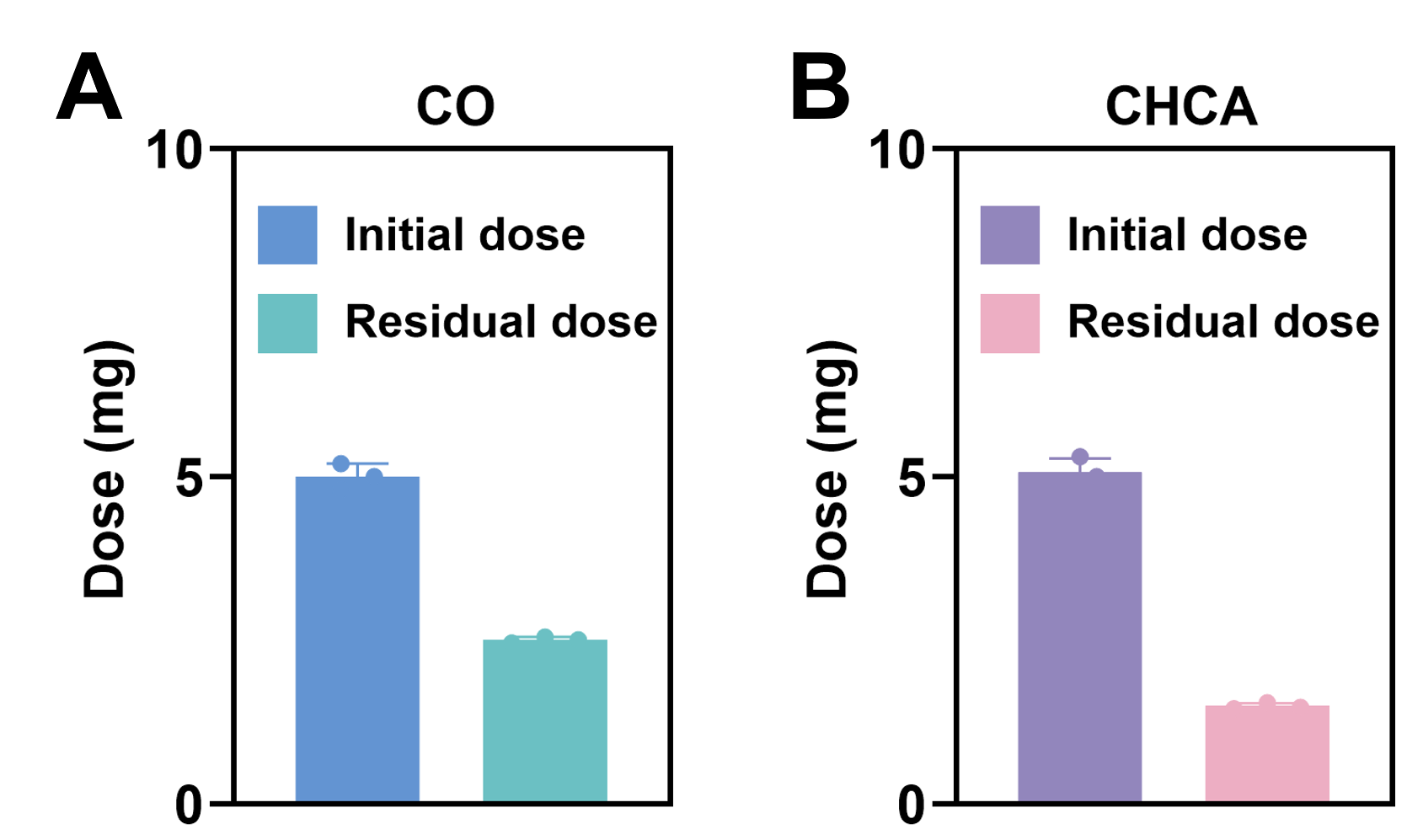


**Figure S5.** Comparison between the initial dose and residual dose of (A) CO and (B) CHCA. Data are expressed as mean ± standard deviation (SD, n=3).


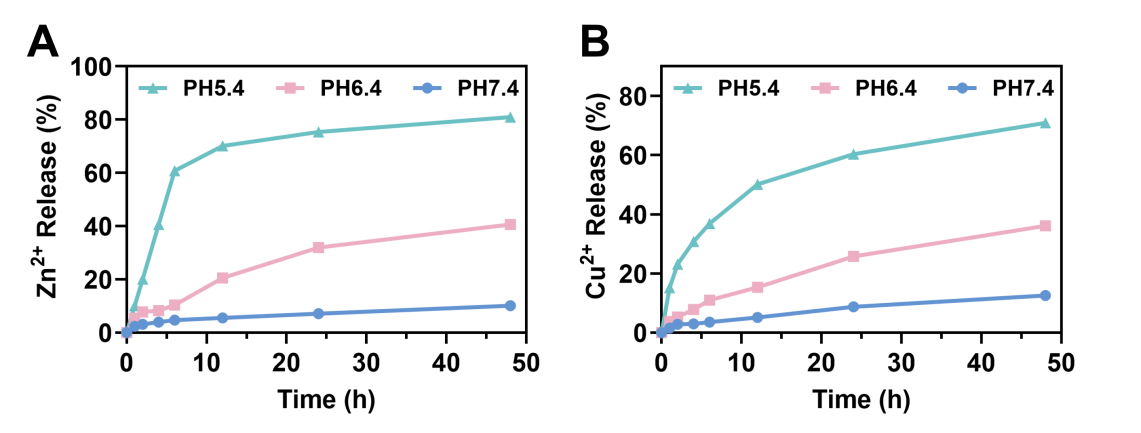


**Figure S6.** Accumulative release profiles of Zn^2+^, and Cu^2+^ from PEG@AuCZ@CC under different times and different pH.


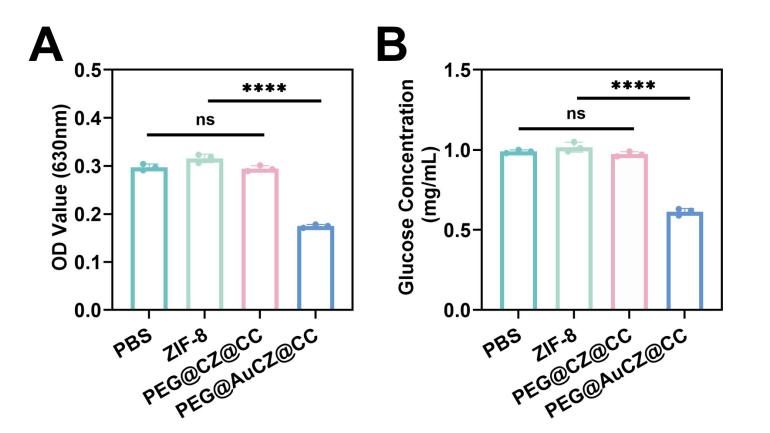


**Figure S7.** Test of glucose consumption by PEG@AuCZ@CC NPs in glucose solutions. Data are expressed as mean ± standard deviation (SD, n=3). One-way Anova or t-test was used to analyze statistical differences between groups. *P < 0.05, **P < 0.01, ***P < 0.001, ****p < 0.0001. "ns" denotes no significant difference.


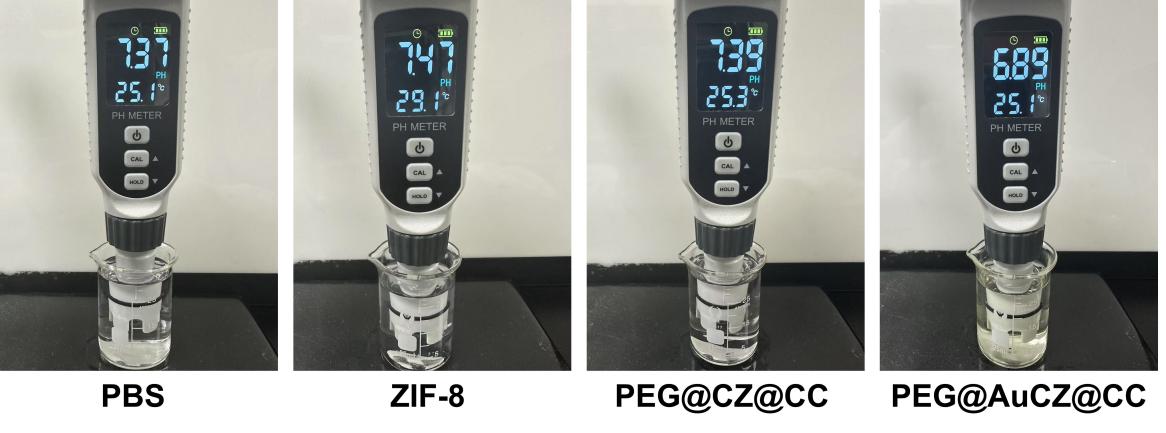


**Figure S8.** The pH variations across different solution systems.


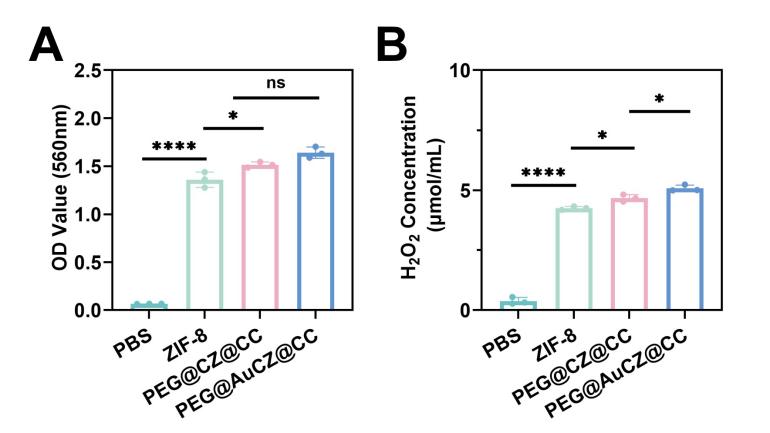


**Figure S9.** H_2_O_2_-generating ability under various treatments. Data are expressed as mean ± standard deviation (SD, n=3). One-way Anova or t-test was used to analyze statistical differences between groups. *P < 0.05, **P < 0.01, ***P < 0.001, ****p < 0.0001. "ns" denotes no significant difference.


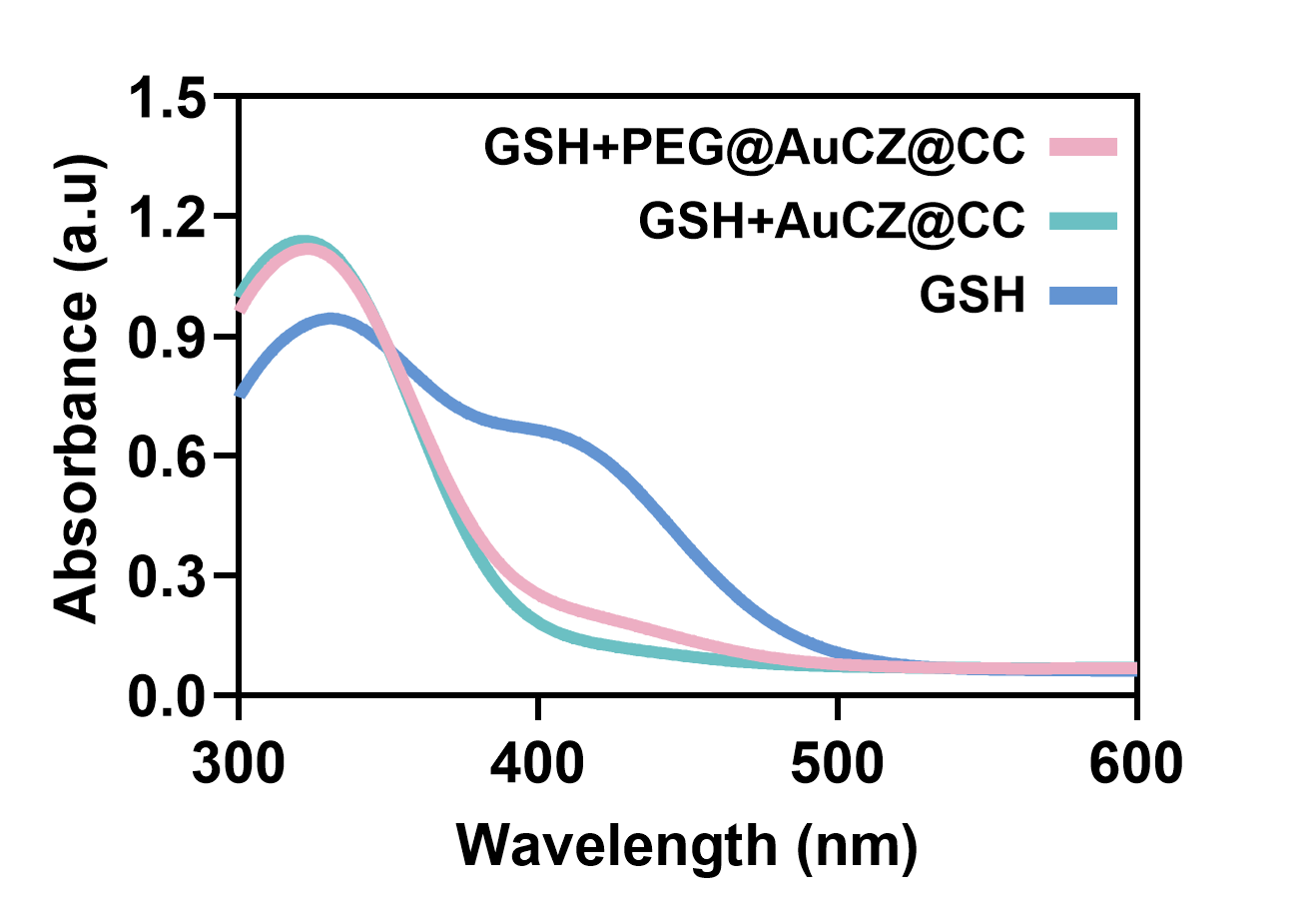


**Figure S10.** UV-vis spectra of DTNB in the presence of GSH and AuCZ@CC NPs, PEG@AuCZ@CC NPs.


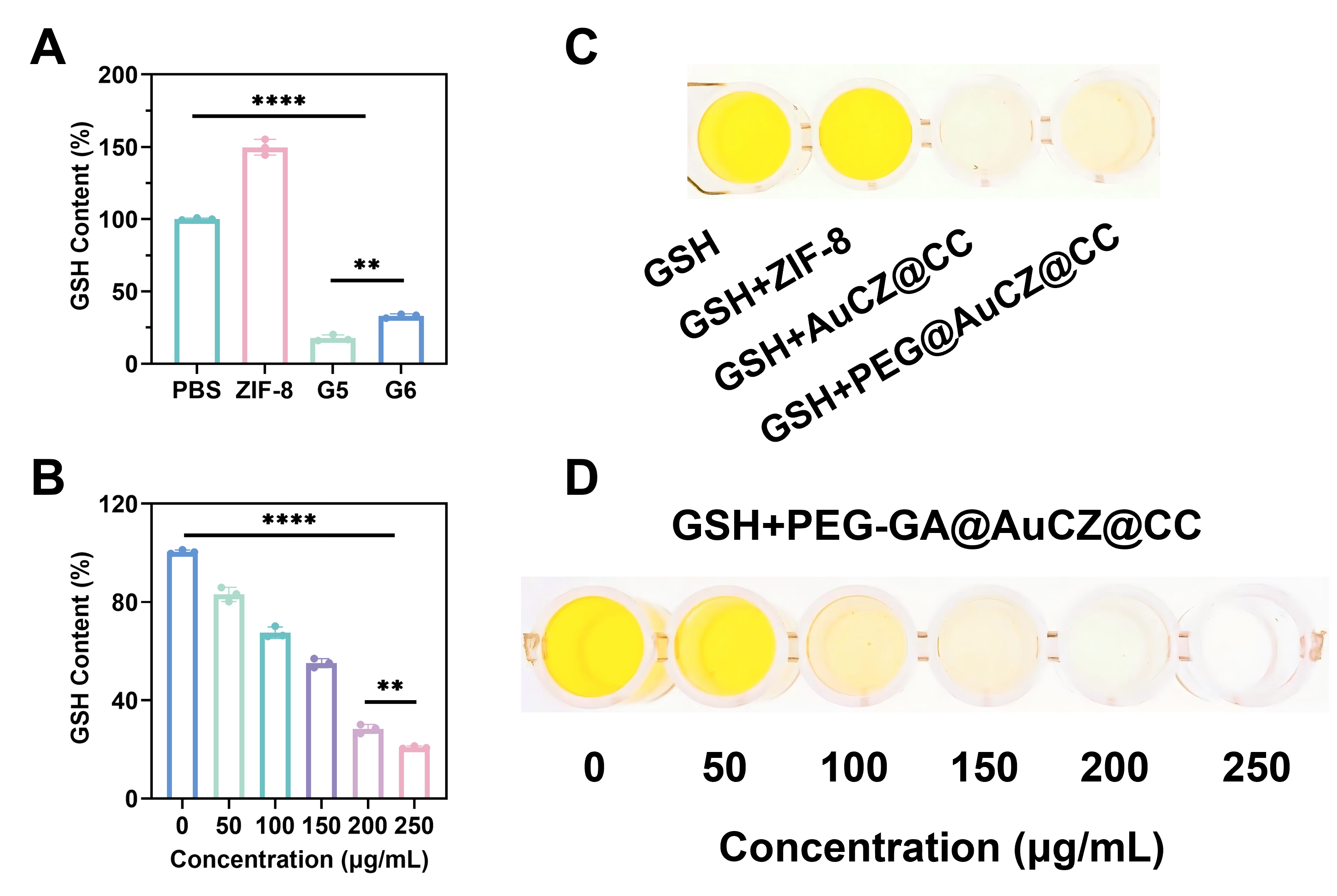


**Figure S11.** The concentration of GSH under (A) different Nanomedicines and (B)different concentrations of PEG@AuCZ@CC NPs. (C, D) The colorimetric evolution of the reaction system. G5: AuCZ@CC, G6: PEG@AuCZ@CC. Data are expressed as mean ± standard deviation (SD, n=3). One-way Anova or t-test was used to analyze statistical differences between groups. *P < 0.05, **P < 0.01, ***P < 0.001, ****p < 0.0001. "ns" denotes no significant difference.


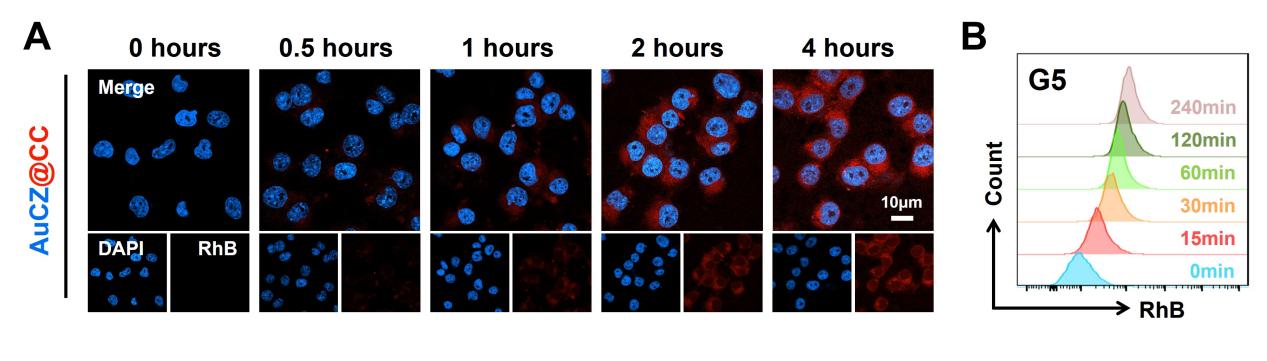


**Figure S12. (A)** CLSM images of Hepa1-6 cells following incubation with Rhodamine B-labeled AuCZ@CC NPs for different durations, with cell nuclei stained using DAPI (Scale bar: 10 µm). **(B)** Flow cytometry analysis of cellular uptake efficiency of Rhodamine-B labeled AuCZ@CC NPs.


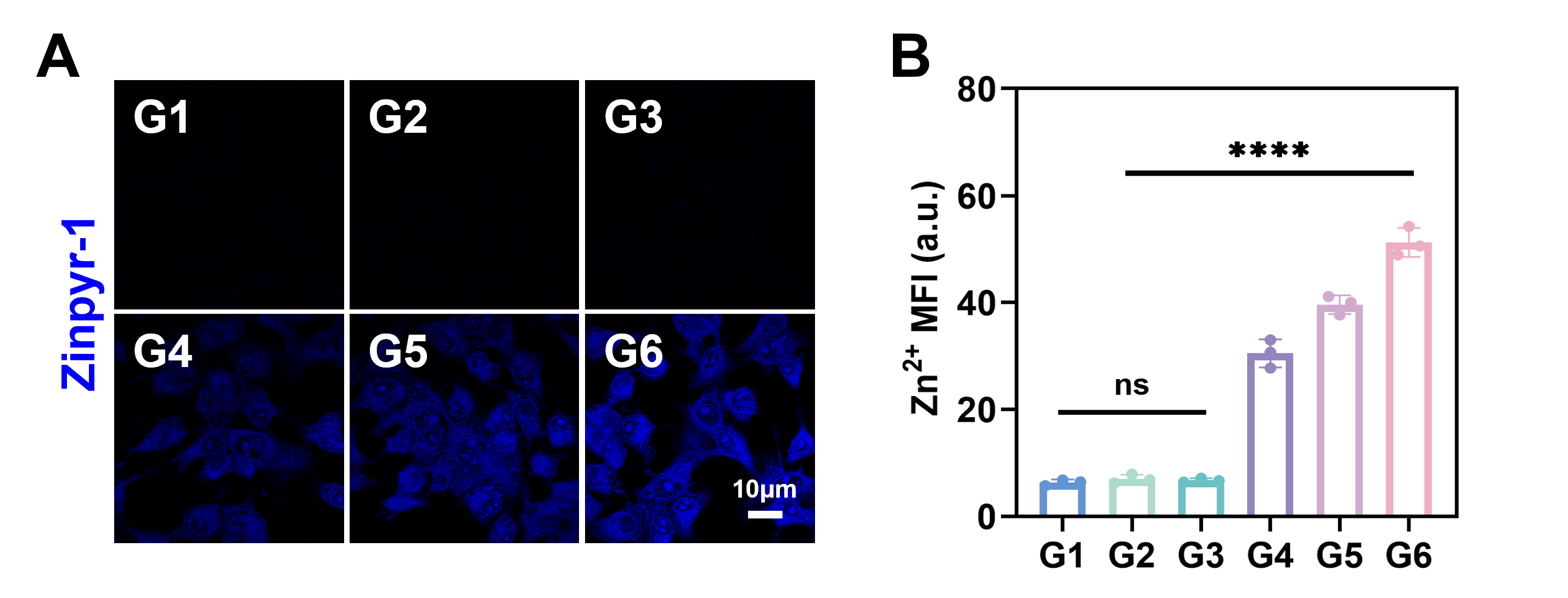


**Figure S13.** (A) CLSM images and (B) Semi-quantitative analysis of Hepa1-6 cells stained with Zinpyr-1 after different treatments (Scale bar: 10 µm). Data are expressed as mean ± SD (n = 3). One-way Anova or t-test was used to analyze statistical differences between groups. *P < 0.05, **P < 0.01, ***P < 0.001, ****p < 0.0001. "ns" denotes no significant difference. Note, G1: Control, G2: CO, G3: CO&CHCA, G4: CZ@CC, G5: AuCZ@CC, G6: PEG@AuCZ@CC NPs.


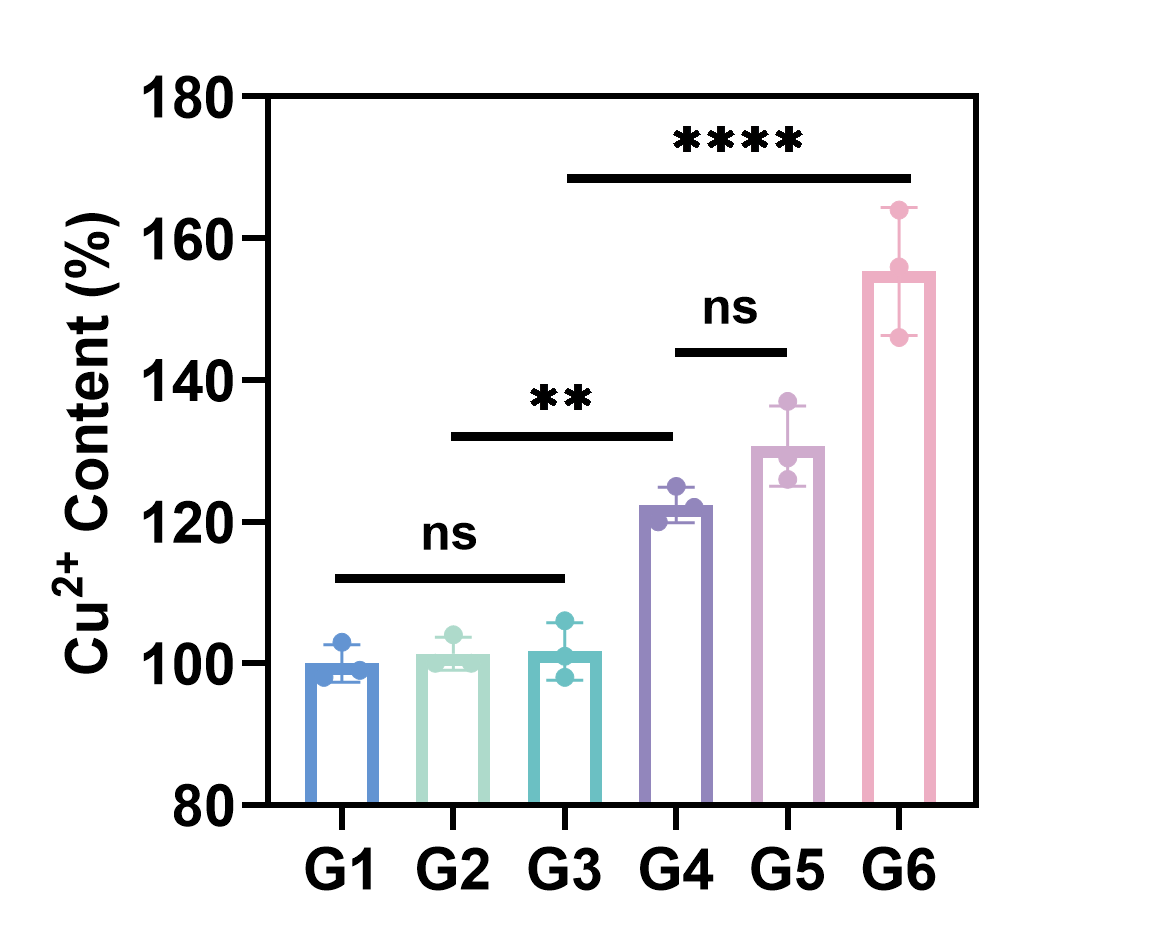


**Figure S14.** The relative Cu^2+^ content in Hepa1-6 cells with various treatments. Data are expressed as mean ± SD (n = 3). One-way Anova or t-test was used to analyze statistical differences between groups. *P < 0.05, **P < 0.01, ***P < 0.001, ****p < 0.0001. "ns" denotes no significant difference. Note, G1: Control, G2: CO, G3: CO&CHCA, G4: CZ@CC, G5: AuCZ@CC, G6: PEG@AuCZ@CC NPs.


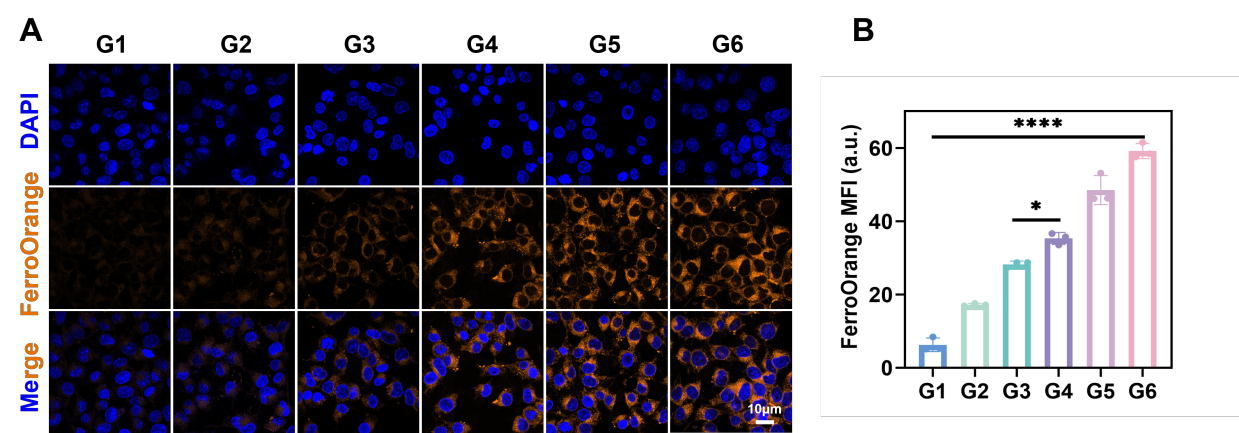


**Figure S15.** (A) CLSM images and (B) semi-quantitative analysis of Hepa1-6 cells stained with FerroOrange fluorescence after different treatments, wherein cell nuclei are stained with DAPI. (Scale bar: 10 µm). Data are expressed as mean ± SD (n = 3). One-way Anova or t-test was used to analyze statistical differences between groups. *P < 0.05, **P < 0.01, ***P < 0.001, ****p < 0.0001. "ns" denotes no significant difference. Note, G1: Control, G2: CO, G3: CO&CHCA, G4: CZ@CC, G5: AuCZ@CC, G6: PEG@AuCZ@CC NPs.


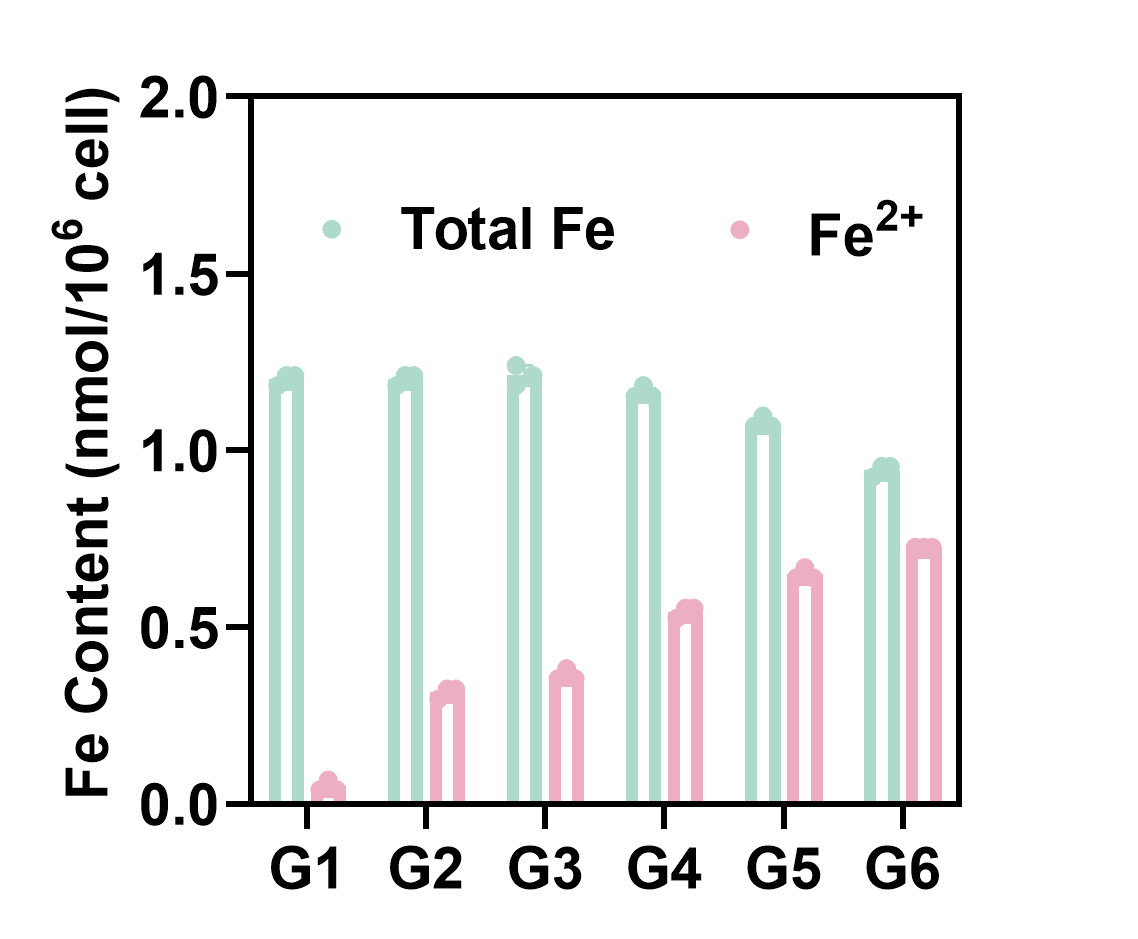


**Figure S16.** The relative Total iron and Fe^2+^ content in Hepa1-6 cells with various treatments. Data are expressed as mean ± SD (n = 3). One-way Anova or t-test was used to analyze statistical differences between groups. *P < 0.05, **P < 0.01, ***P < 0.001, ****p < 0.0001. "ns" denotes no significant difference. Note, G1: Control, G2: CO, G3: CO&CHCA, G4: CZ@CC, G5: AuCZ@CC, G6: PEG@AuCZ@CC NPs.


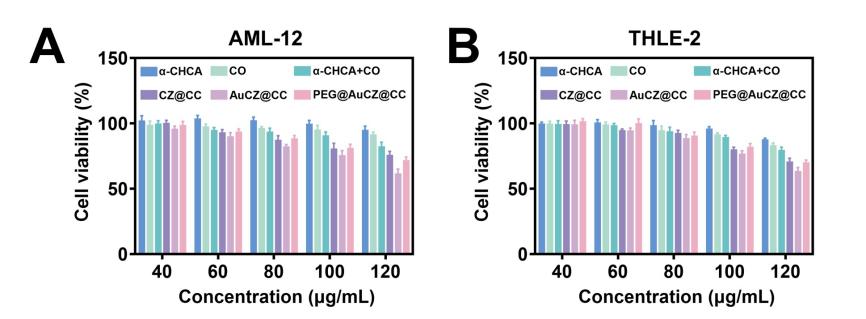


**Figure S17.** Cytotoxicity assay of **(A)** AML-12 cells and **(B)** THLE-2 cells after different treatments for 24 h, n = 6.


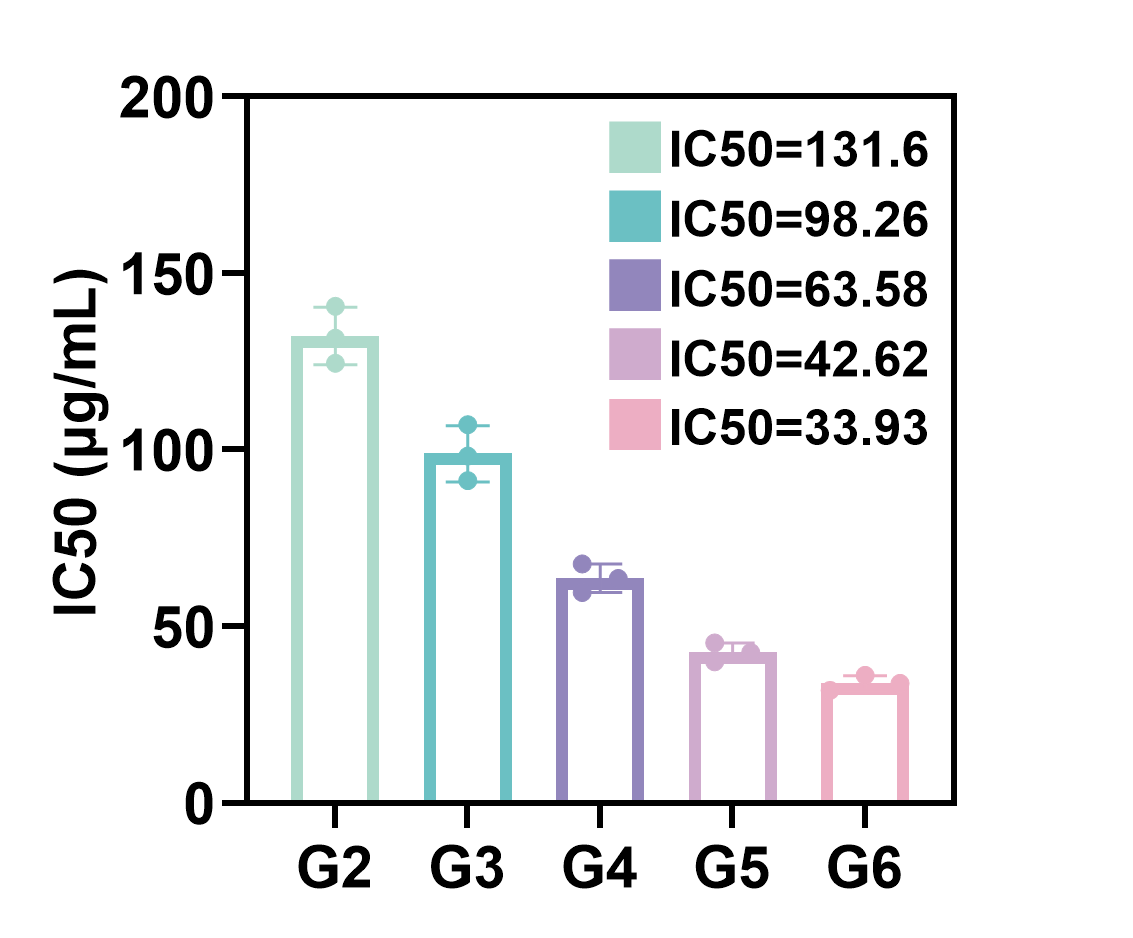


**Figure S18.** The IC50 of various Nanomedicines. Data are expressed as mean ± SD (n = 6). Note, G2: CO, G3: CO&CHCA, G4: CZ@CC, G5: AuCZ@CC, G6: PEG@AuCZ@CC NPs.


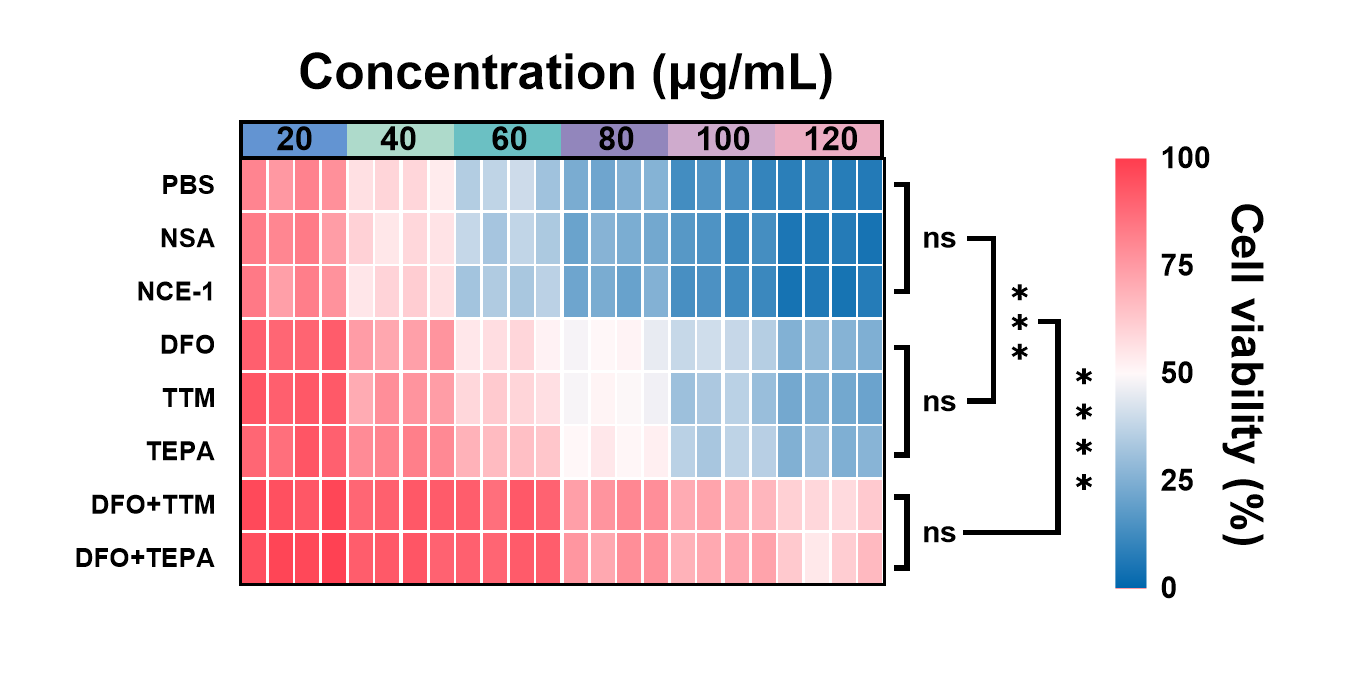


**Figure S19.** Cell viability of Hepa1-6 cells after varied treatments for 24 h, n = 4. One-way Anova or t-test was used to analyze statistical differences between groups. *P < 0.05, **P < 0.01, ***P < 0.001, ****p < 0.0001. "ns" denotes no significant difference.


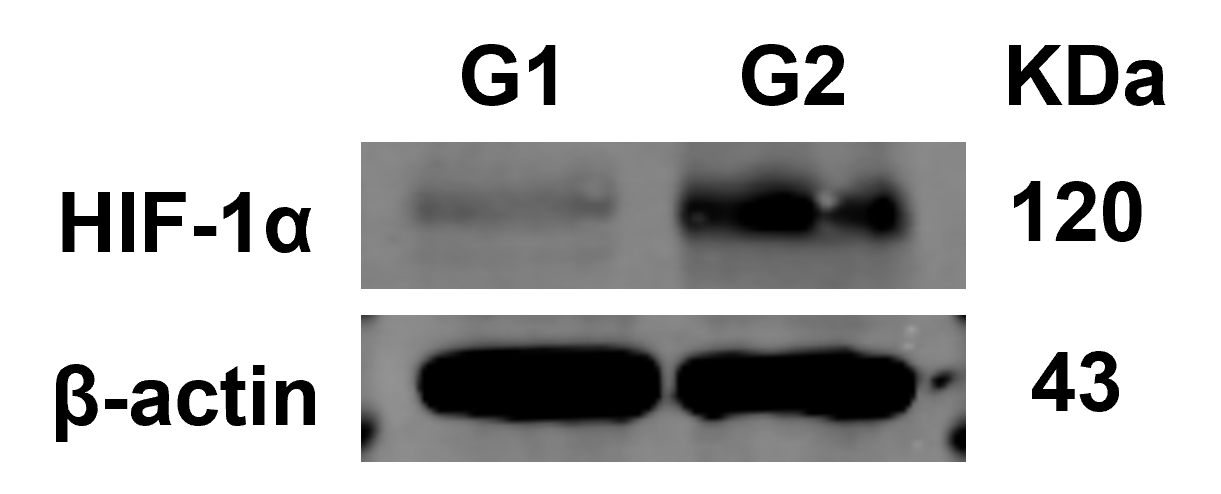


**Figure S20.** Western blot bands of HIF-1α in Hepa1-6 cells cultured in standard medium or CoCl_2_-supplemented medium for 48 hours. G1: standard medium, G2: CoCl_2_-supplemented medium.


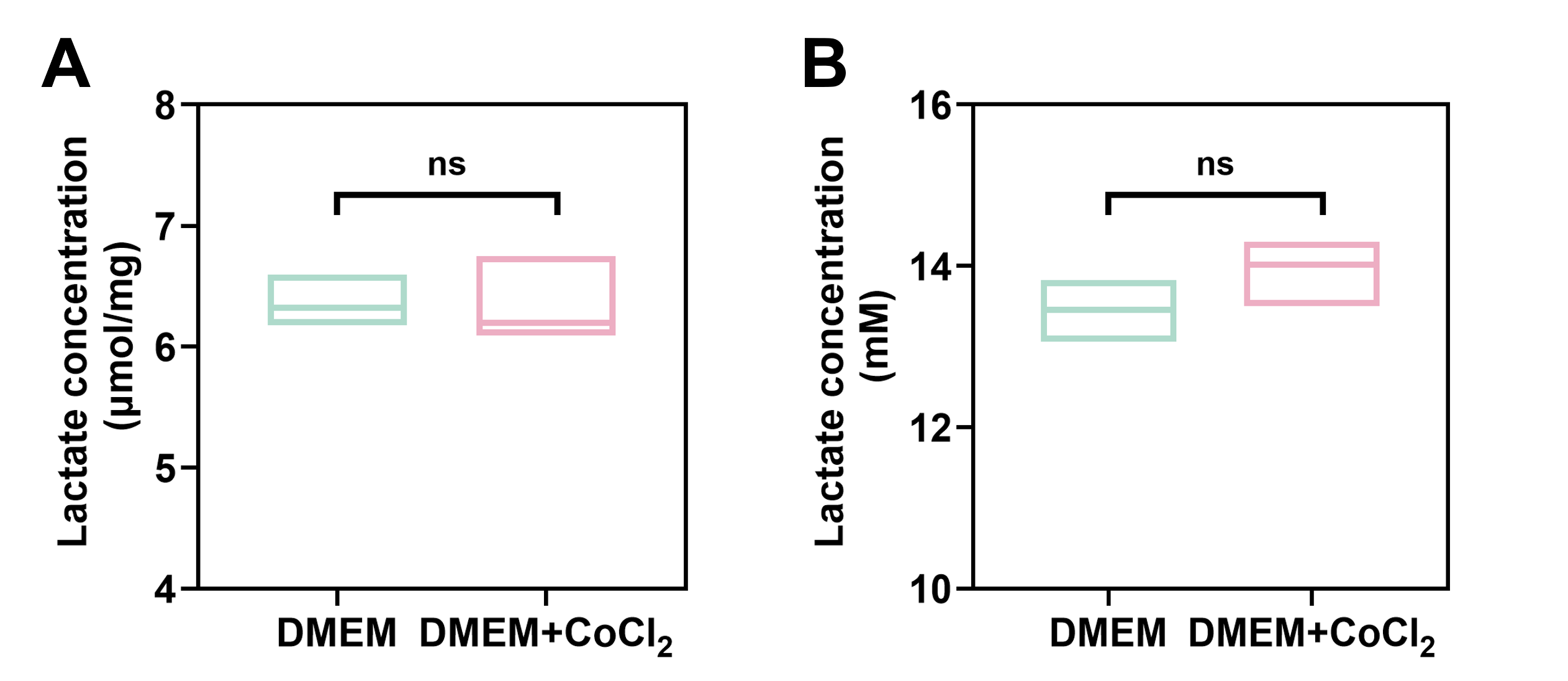


**Figure S21.** The levels of lactate measured in the (A) cellular lysates and (B) culture mediums after various treatments. Data are expressed as mean ± SD (n = 3). One-way Anova or t-test was used to analyze statistical differences between groups. *P < 0.05, **P < 0.01, ***P < 0.001, ****p < 0.0001. "ns" denotes no significant difference.


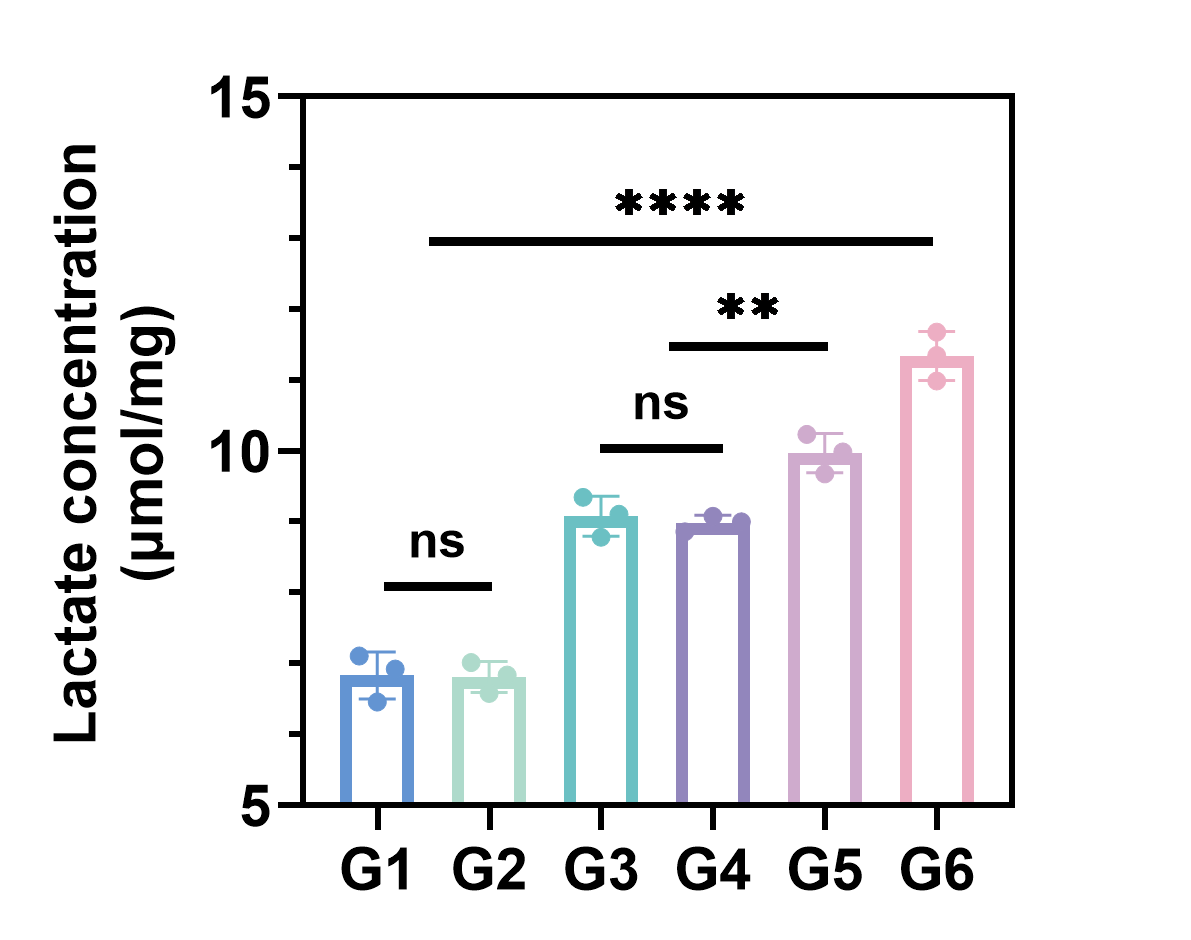


**Figure S22.** The levels of lactate measured in the cellular lysates after various treatments. Data are expressed as mean ± SD (n = 3). One-way Anova or t-test was used to analyze statistical differences between groups. *P < 0.05, **P < 0.01, ***P < 0.001, ****p < 0.0001. "ns" denotes no significant difference. Note, G1: Control, G2: CO, G3: CO&CHCA, G4: CZ@CC, G5: AuCZ@CC, G6: PEG@AuCZ@CC NPs.


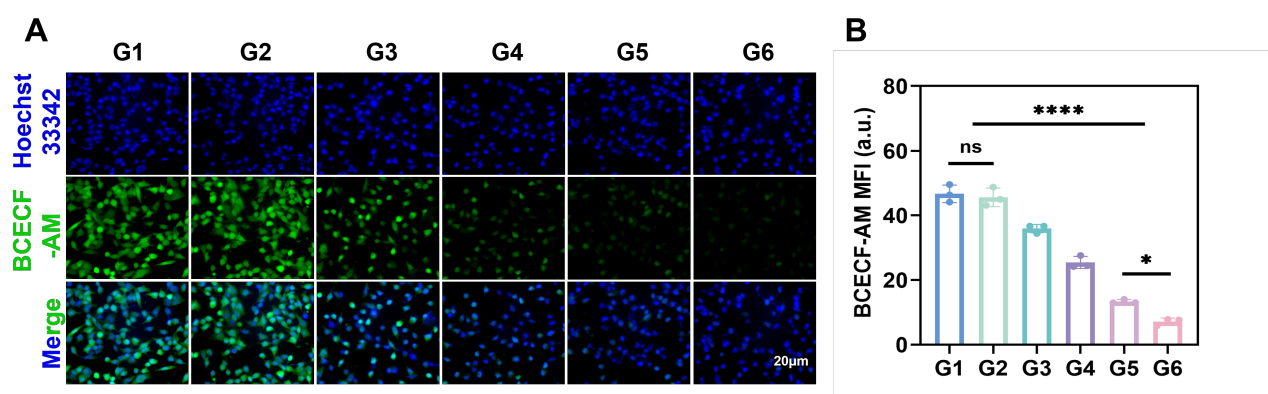


**Figure S23. (A)** Fluorescence inverted microscopy images and **(B)** semi-quantitative analysis of Hepa1-6 cells stained with BCECF-AM after different treatments, wherein cell nuclei are stained with Hoechst 33342. (Scale bar: 20 µm). Data are expressed as mean ± SD (n = 3). One-way Anova or t-test was used to analyze statistical differences between groups. *P < 0.05, **P < 0.01, ***P < 0.001, ****p < 0.0001. "ns" denotes no significant difference. Note, G1: Control, G2: CO, G3: CO&CHCA, G4: CZ@CC, G5: AuCZ@CC, G6: PEG@AuCZ@CC NPs.


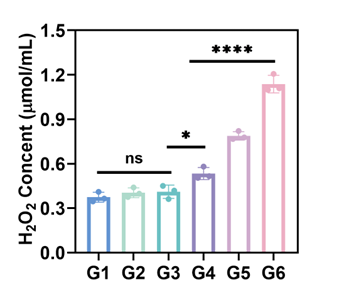


**Figure S24.** The H_2_O_2_ content in Hepa1-6 cells with various treatments. Data are expressed as mean ± SD (n = 3). One-way Anova or t-test was used to analyze statistical differences between groups. *P < 0.05, **P < 0.01, ***P < 0.001, ****p < 0.0001. "ns" denotes no significant difference. Note, G1: Control, G2: CO, G3: CO&CHCA, G4: CZ@CC, G5: AuCZ@CC, G6: PEG@AuCZ@CC NPs.


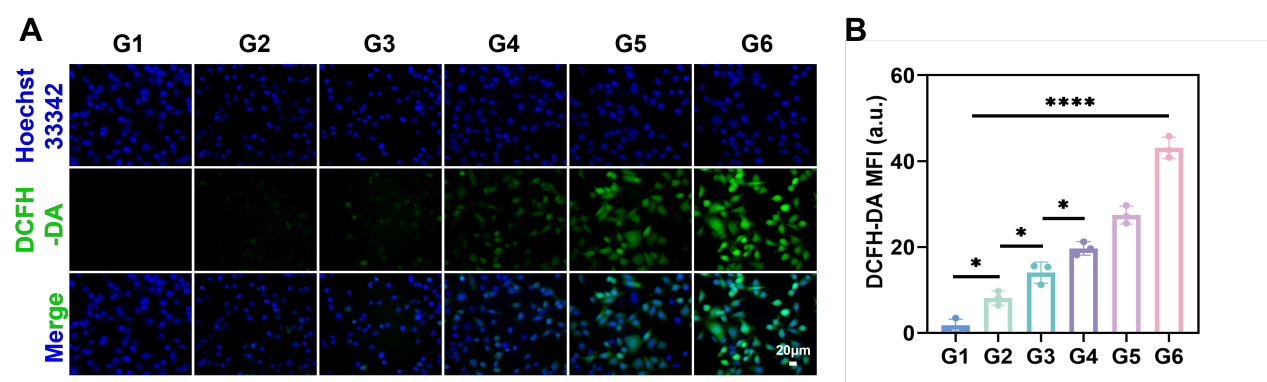


**Figure S25. (A)** Fluorescence inverted microscopy images and **(B)** semi-quantitative analysis of Hepa1-6 cells stained with DCFH-DA after different treatments, wherein cell nuclei are stained with Hoechst 33342. (Scale bar: 20 µm). Data are expressed as mean ± SD (n = 3). One-way Anova or t-test was used to analyze statistical differences between groups. *P < 0.05, **P < 0.01, ***P < 0.001, ****p < 0.0001. "ns" denotes no significant difference. Note, G1: Control, G2: CO, G3: CO&CHCA, G4: CZ@CC, G5: AuCZ@CC, G6: PEG@AuCZ@CC NPs.


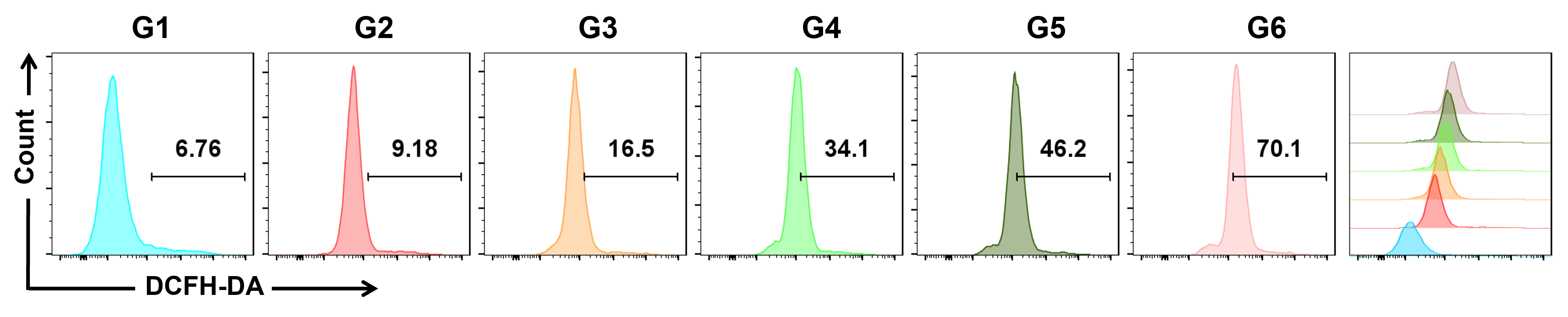


**Figure S26.** Analysis of intracellular ROS levels by flow cytometry. Note, G1: Control, G2: CO, G3: CO&CHCA, G4: CZ@CC, G5: AuCZ@CC, G6: PEG@AuCZ@CC NPs.


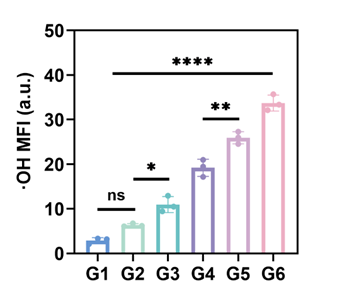


**Figure S27.** Semi-quantitative analysis of Hepa1-6 cells stained with hydroxyl radicals after different treatments. Data are expressed as mean ± SD (n = 3). One-way Anova or t-test was used to analyze statistical differences between groups. *P < 0.05, **P < 0.01, ***P < 0.001, ****p < 0.0001. "ns" denotes no significant difference. Note, G1: Control, G2: CO, G3: CO&CHCA, G4: CZ@CC, G5: AuCZ@CC, G6: PEG@AuCZ@CC NPs.


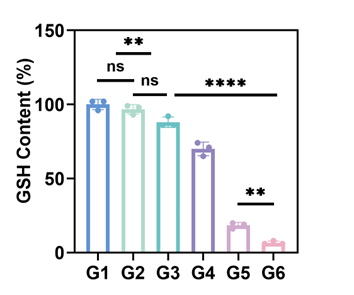


**Figure S28.** Relative intracellular GSH content of Hepa1-6 cells after different treatments. Data are expressed as mean ± SD (n = 3). One-way Anova or t-test was used to analyze statistical differences between groups. *P < 0.05, **P < 0.01, ***P < 0.001, ****p < 0.0001. "ns" denotes no significant difference. Note, G1: Control, G2: CO, G3: CO&CHCA, G4: CZ@CC, G5: AuCZ@CC, G6: PEG@AuCZ@CC NPs.


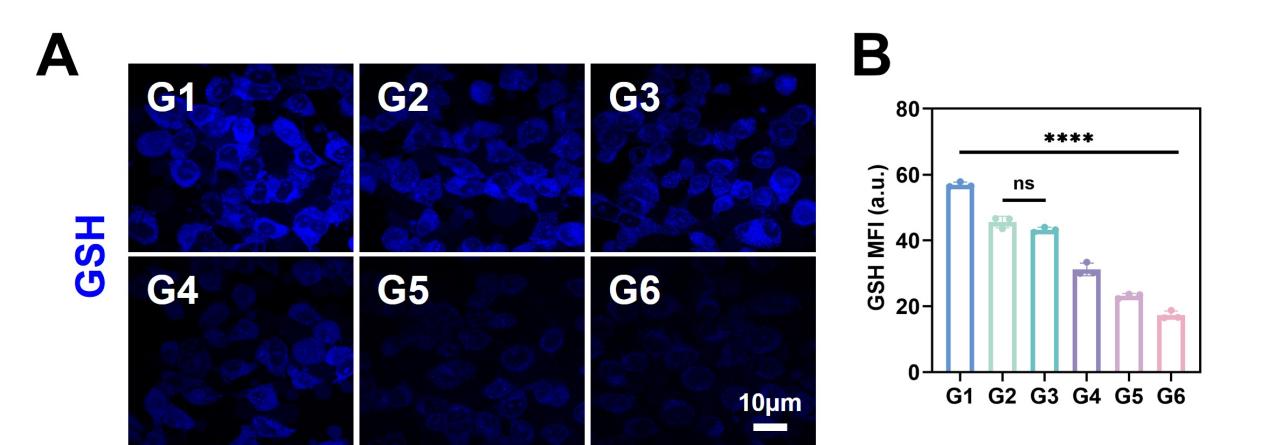


**Figure S29. (A)** CLSM images and **(B)** Semi-quantitative analysis of Hepa1-6 cells stained with GSH probe after different treatments. (Scale bar: 10 µm). Data are expressed as mean ± SD (n = 3). One-way Anova or t-test was used to analyze statistical differences between groups. *P < 0.05, **P < 0.01, ***P < 0.001, ****p < 0.0001. "ns" denotes no significant difference. Note, G1: Control, G2: CO, G3: CO&CHCA, G4: CZ@CC, G5: AuCZ@CC, G6: PEG@AuCZ@CC NPs.


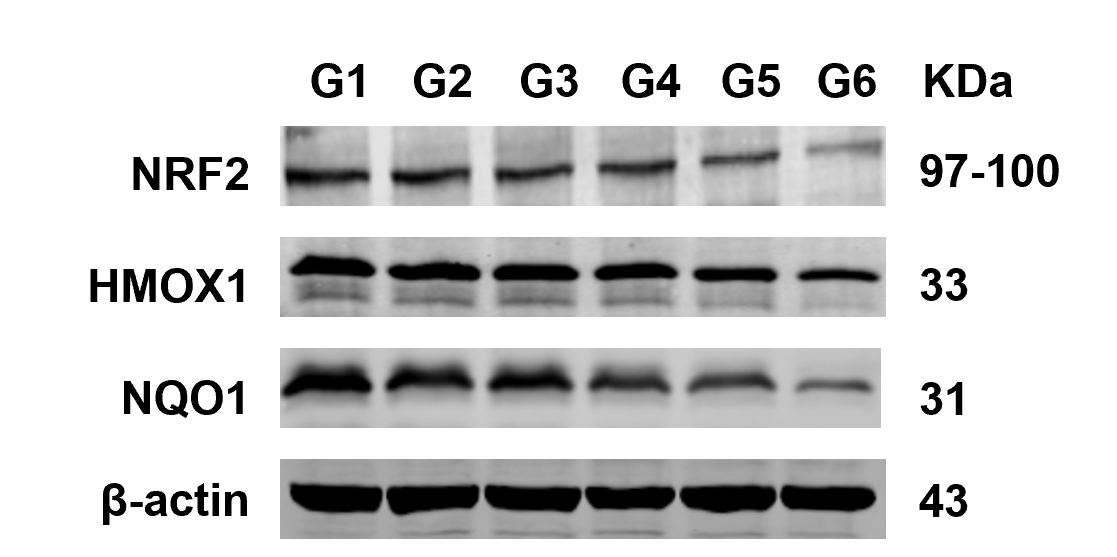


**Figure S30.** Western blotting analysis of Nrf2, HMOX1, and NQO1 in Hepa1-6 cells after different treatments. G1: Control, G2: CO, G3: CO&CHCA, G4: CZ@CC, G5: AuCZ@CC, G6: PEG@AuCZ@CC NPs.


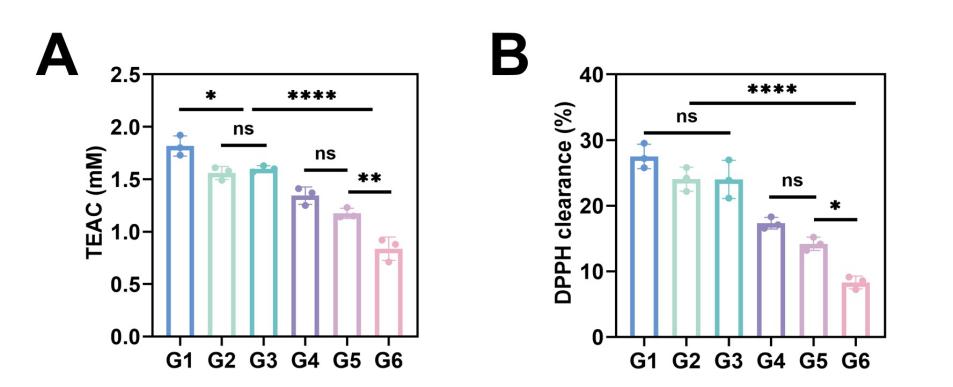


**Figure S31. (A)** The T-AOC assay was used to measure the antioxidant capacity of Hepa1-6 cells. **(B)** The DPPH free radical scavenging rate in Hepa1-6 cells. Data are expressed as mean ± SD (n = 3). One-way Anova or t-test was used to analyze statistical differences between groups. *P < 0.05, **P < 0.01, ***P < 0.001, ****p < 0.0001. "ns" denotes no significant difference. Note, G1: Control, G2: CO, G3: CO&CHCA, G4: CZ@CC, G5: AuCZ@CC, G6: PEG@AuCZ@CC NPs.


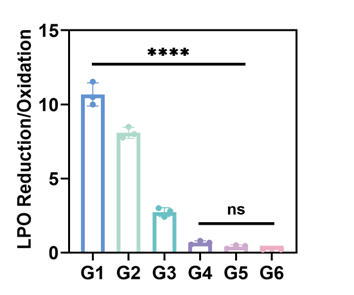


**Figure S32.** Semi-quantitative analysis of Hepa1-6 cells stained with C11-BODIPY fluorescent probe after different treatments. Data are expressed as mean ± SD (n = 3). One-way Anova or t-test was used to analyze statistical differences between groups. *P < 0.05, **P < 0.01, ***P < 0.001, ****p < 0.0001. "ns" denotes no significant difference. Note, G1: Control, G2: CO, G3: CO&CHCA, G4: CZ@CC, G5: AuCZ@CC, G6: PEG@AuCZ@CC NPs.


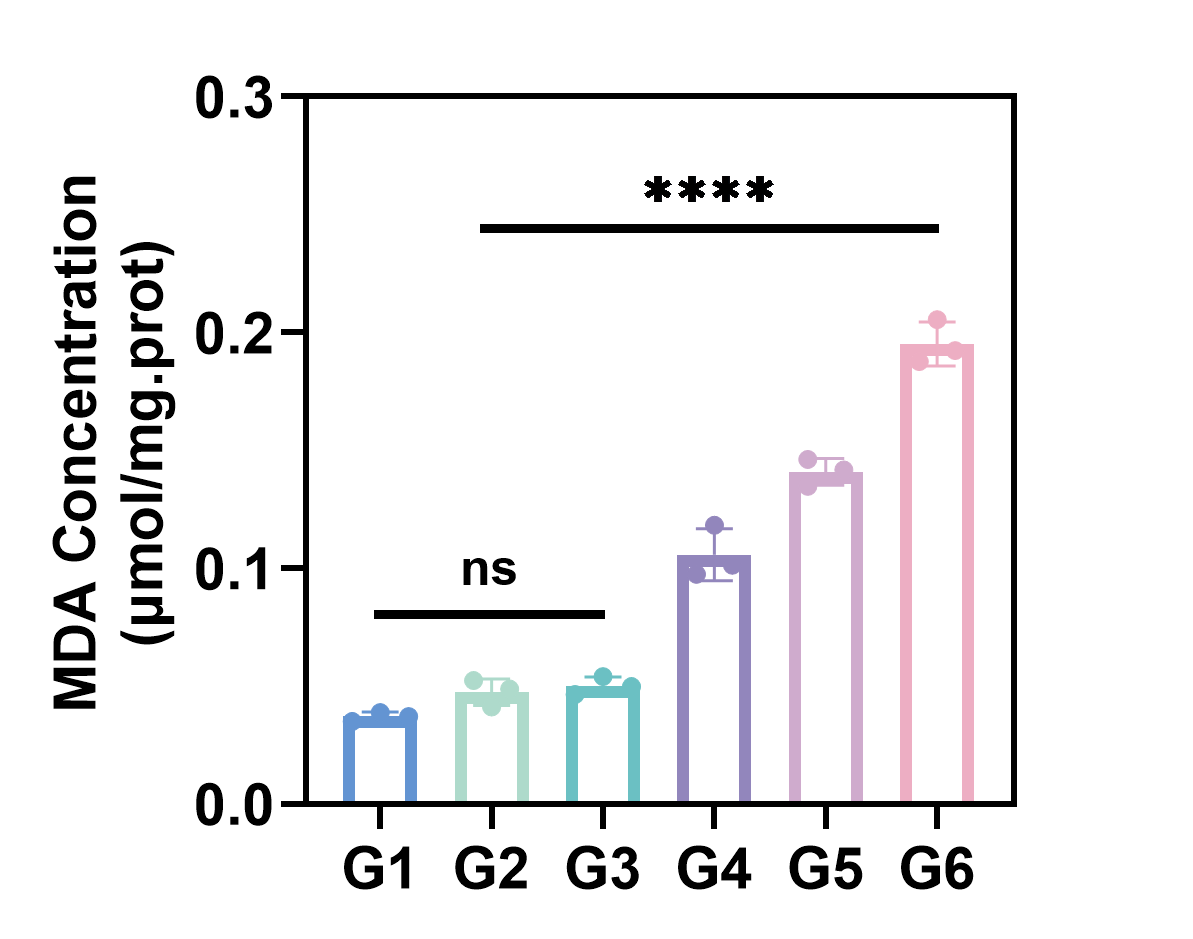


**Figure S33.** The MDA content in Hepa1-6 cells with various treatments. Data are expressed as mean ± SD (n = 3). One-way Anova or t-test was used to analyze statistical differences between groups. *P < 0.05, **P < 0.01, ***P < 0.001, ****p < 0.0001. "ns" denotes no significant difference. Note, G1: Control, G2: CO, G3: CO&CHCA, G4: CZ@CC, G5: AuCZ@CC, G6: PEG@AuCZ@CC NPs.


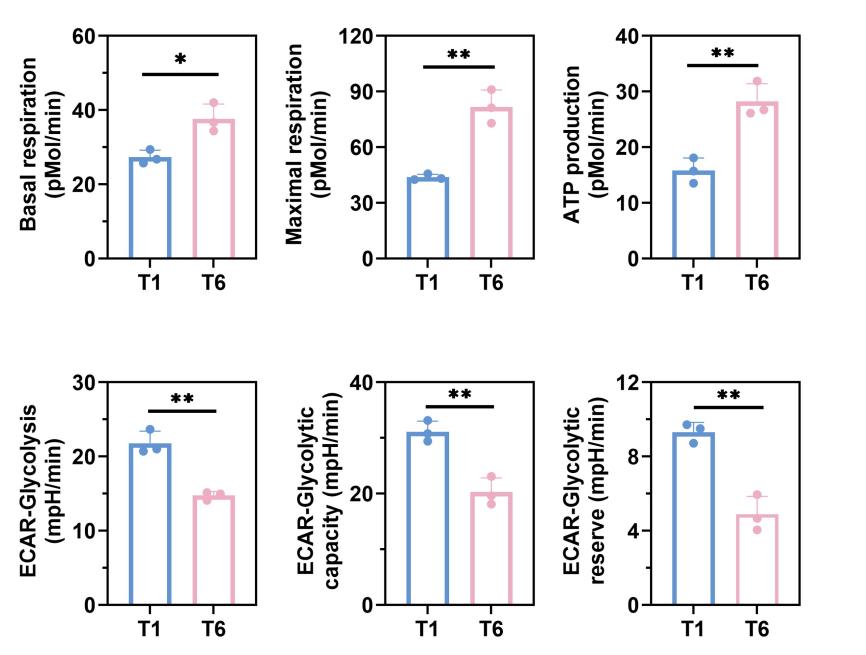


**Figure S34.** Seahorse assay measuring the OCR and ECAR of Hepa1-6 cells repeat treated with PEG@Au/Cu/ZIF-8@CHCA. Data are expressed as mean ± SD (n = 4). One-way Anova or t-test was used to analyze statistical differences between groups. *P < 0.05, **P < 0.01, ***P < 0.001, ****p < 0.0001. "ns" denotes no significant difference.


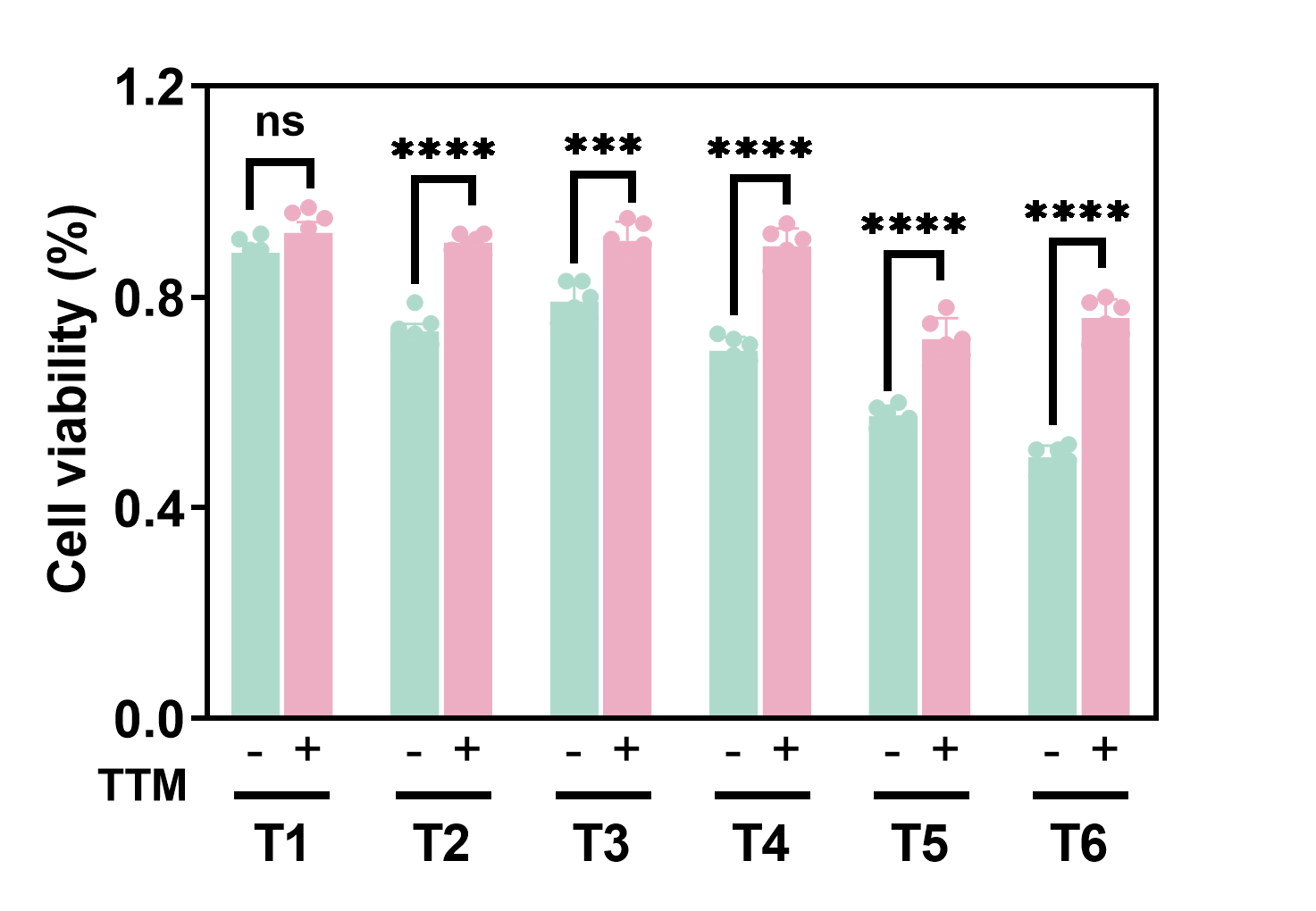


**Figure S35.** Cytotoxicity assay of Hepa1-6 cells after different treatments for 24 h, n = 6. Data are expressed as mean ± SD (n = 3). One-way Anova or t-test was used to analyze statistical differences between groups. *P < 0.05, **P < 0.01, ***P < 0.001, ****p < 0.0001. "ns" denotes no significant difference. Note, T1: Cu/ZIF-8, T2: Cu/ZIF-8@CHCA, T3: Au/Cu/ZIF-8, T4: Au/Cu/ZIF-8@CHCA, T5: PEG@Au/Cu/ZIF-8, T6: PEG@Au/Cu/ZIF-8@CHCA.


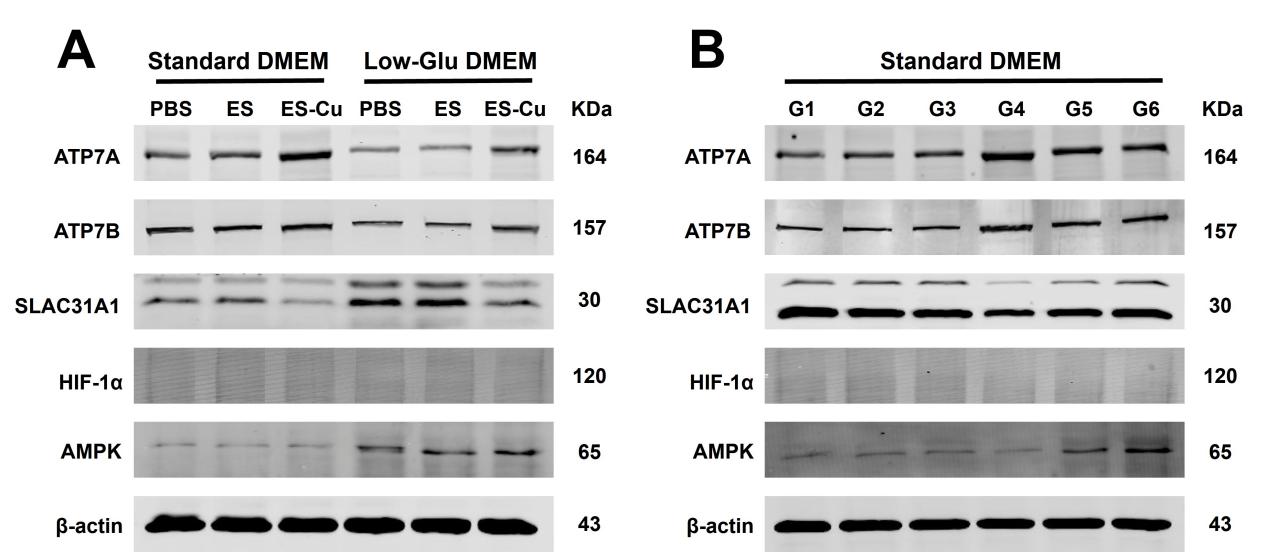


**Figure S36.** Western blotting analysis of ATP7A, ATP7B, and SLC31A1 in Hepa1-6 cells after different treatments.


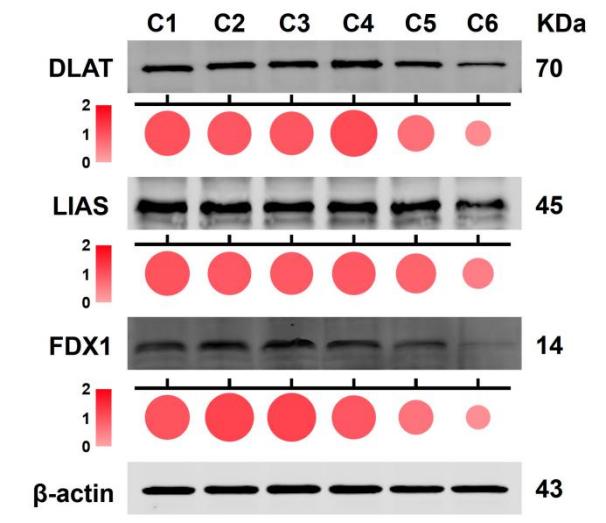


**Figure S37.** Western blotting analysis of DLAT, LIAS, and FDX1 in Hepa1-6 cells after different treatments.


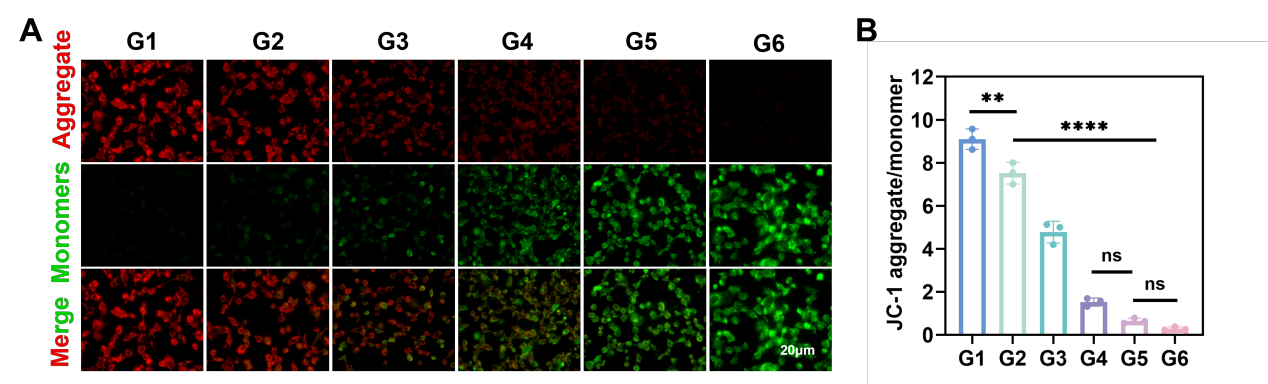


**Figure S38. (A)** Fluorescence inverted microscopy images and **(B)** semi-quantitative analysis illustrating changes in mitochondrial membrane potential in Hepa1-6 cells. (Scale bar: 20 µm). Data are expressed as mean ± SD (n = 3). One-way Anova or t-test was used to analyze statistical differences between groups. *P < 0.05, **P < 0.01, ***P < 0.001, ****p < 0.0001. "ns" denotes no significant difference. Note, G1: Control, G2: CO, G3: CO&CHCA, G4: CZ@CC, G5: AuCZ@CC, G6: PEG@AuCZ@CC NPs.


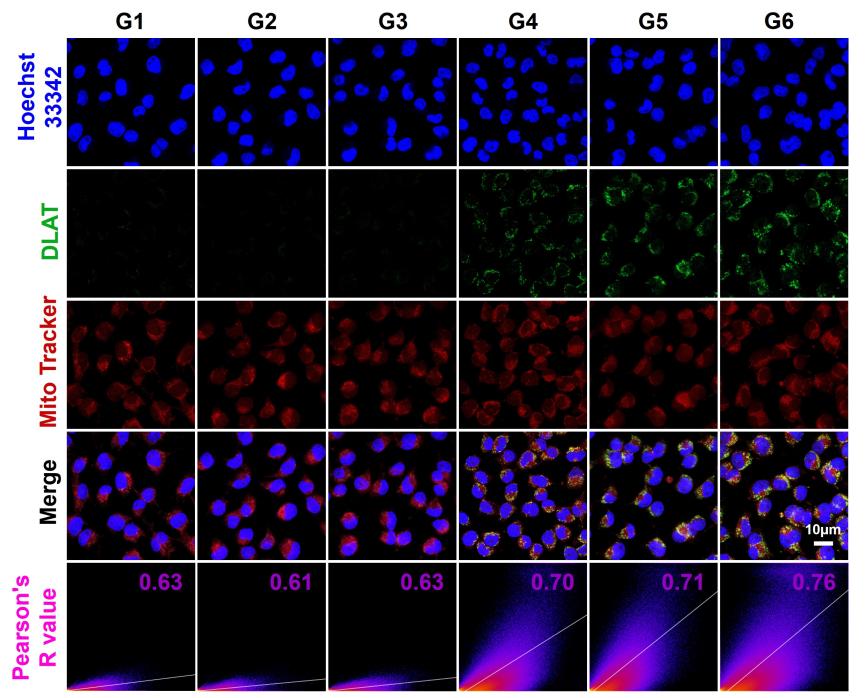


**Figure S39.** Representative CLSM images and Pearson analysis of DLAT (green) and MitoTraker (red) in Hepa1-6 cells with various treatments. (Scale bar: 10 µm).


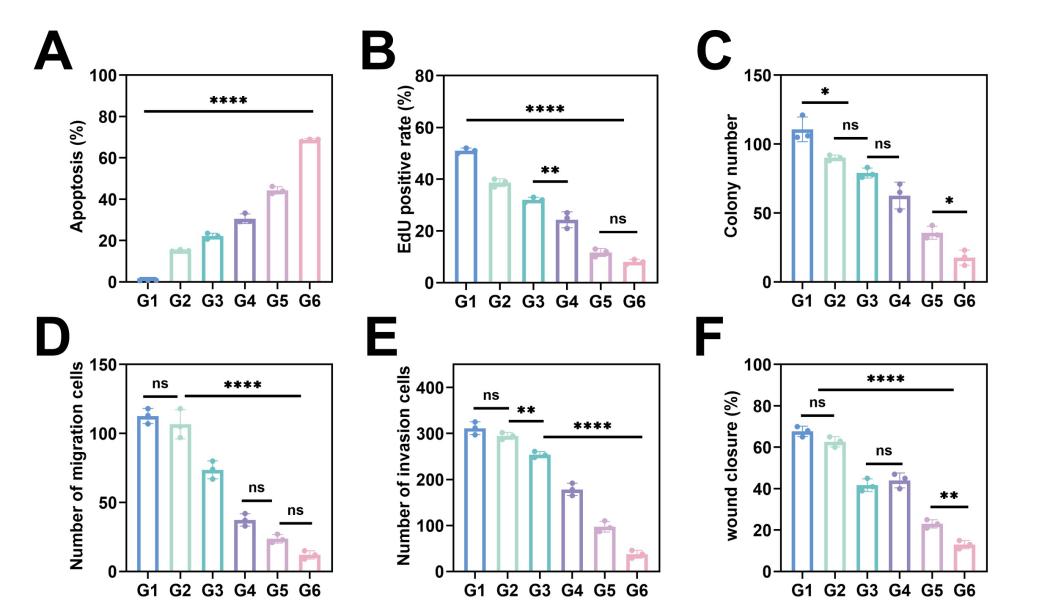


**Figure S40. (A)** The quantitative data analysis of flow cytometry patterns, Hepa1-6 cells stained with propidium iodide (PI) & annexin V-FITC assay kit after different treatments. **(B)** The semi-quantitative analysis of fluorescence inverted microscopy images, Hepa1-6 cells stained with EDU after different treatments. **(C)** The quantitative data of colony test for evaluating Hepa1-6 cells proliferation after different treatments. The quantitative data of **(D)** migration and **(E)** invasion for assessing the metastatic capability of Hepa1-6 cells following different treatments. **(F)** The quantitative data of wound healing assays at 0-, 12- and 24 h post-treatment. Data are expressed as mean ± SD (n = 3). One-way Anova or t-test was used to analyze statistical differences between groups. *P < 0.05, **P < 0.01, ***P < 0.001, ****p < 0.0001. "ns" denotes no significant difference. Note, G1: Control, G2: CO, G3: CO&CHCA, G4: CZ@CC, G5: AuCZ@CC, G6: PEG@AuCZ@CC NPs.


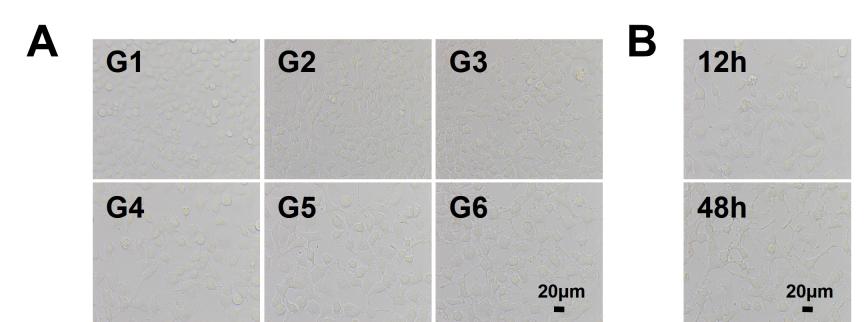


**Figure S41. (A)** The morphology of Hepa1-6 cells after different treatments for 24 h. (Scale bar: 20 µm). **(B)** The morphology of Hepa1-6 cells after treatment for 12 h and 48 h. (Scale bar: 20 µm).


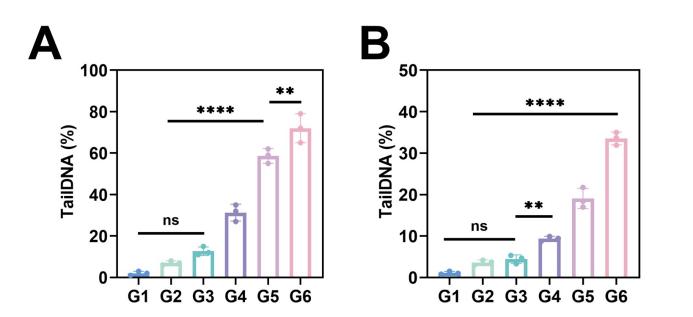


**Figure S42.** The semi-quantitative analysis of comet assay for evaluating the DNA damage of Hepa1-6 cells after different treatments. Data are expressed as mean ± SD (n = 3). One-way Anova or t-test was used to analyze statistical differences between groups. *P < 0.05, **P < 0.01, ***P < 0.001, ****p < 0.0001. "ns" denotes no significant difference. Note, G1: Control, G2: CO, G3: CO&CHCA, G4: CZ@CC, G5: AuCZ@CC, G6: PEG@AuCZ@CC NPs.


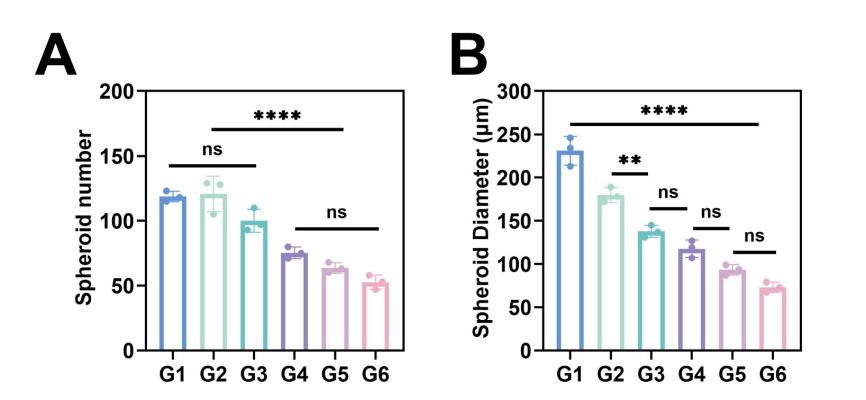


**Figure S43.** Tumor spheroid assays of Hepa1-6 cells after different treatments. Data are expressed as mean ± SD (n = 3). One-way Anova or t-test was used to analyze statistical differences between groups. *P < 0.05, **P < 0.01, ***P < 0.001, ****p < 0.0001. "ns" denotes no significant difference. Note, G1: Control, G2: CO, G3: CO&CHCA, G4: CZ@CC, G5: AuCZ@CC, G6: PEG@AuCZ@CC NPs.


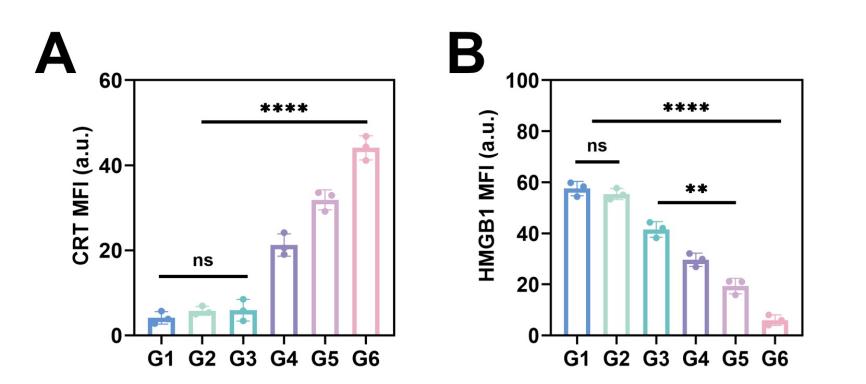


**Figure S44. (A)** Semi-quantitative analysis of CRT localization on the cell membranes of Hepa1-6 cells after different treatments. **(B)** Semi-quantitative analysis of HMGB1 localization in the nucleus of Hepa1-6 cells after different treatments. Data are expressed as mean ± SD (n = 3). One-way Anova or t-test was used to analyze statistical differences between groups. *P < 0.05, **P < 0.01, ***P < 0.001, ****p < 0.0001. "ns" denotes no significant difference. Note, G1: Control, G2: CO, G3: CO&CHCA, G4: CZ@CC, G5: AuCZ@CC, G6: PEG@AuCZ@CC NPs.


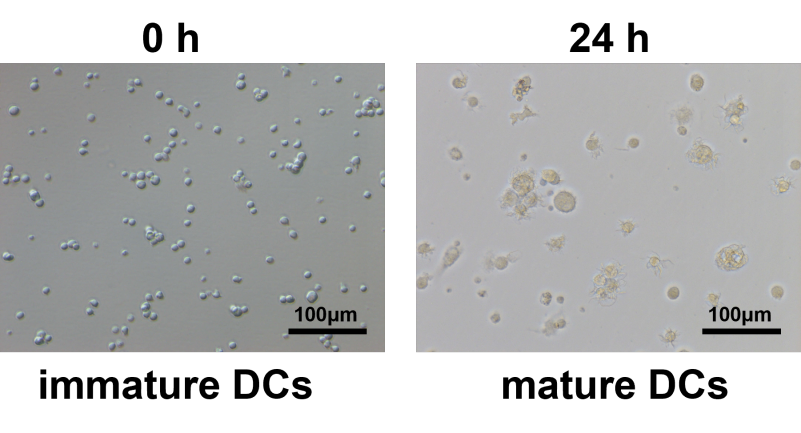


**Figure S45.** The morphological transformation of immature DCs to mature DCs after co-incubation with Hepa1-6 cells. (Scale bar: 100 µm).


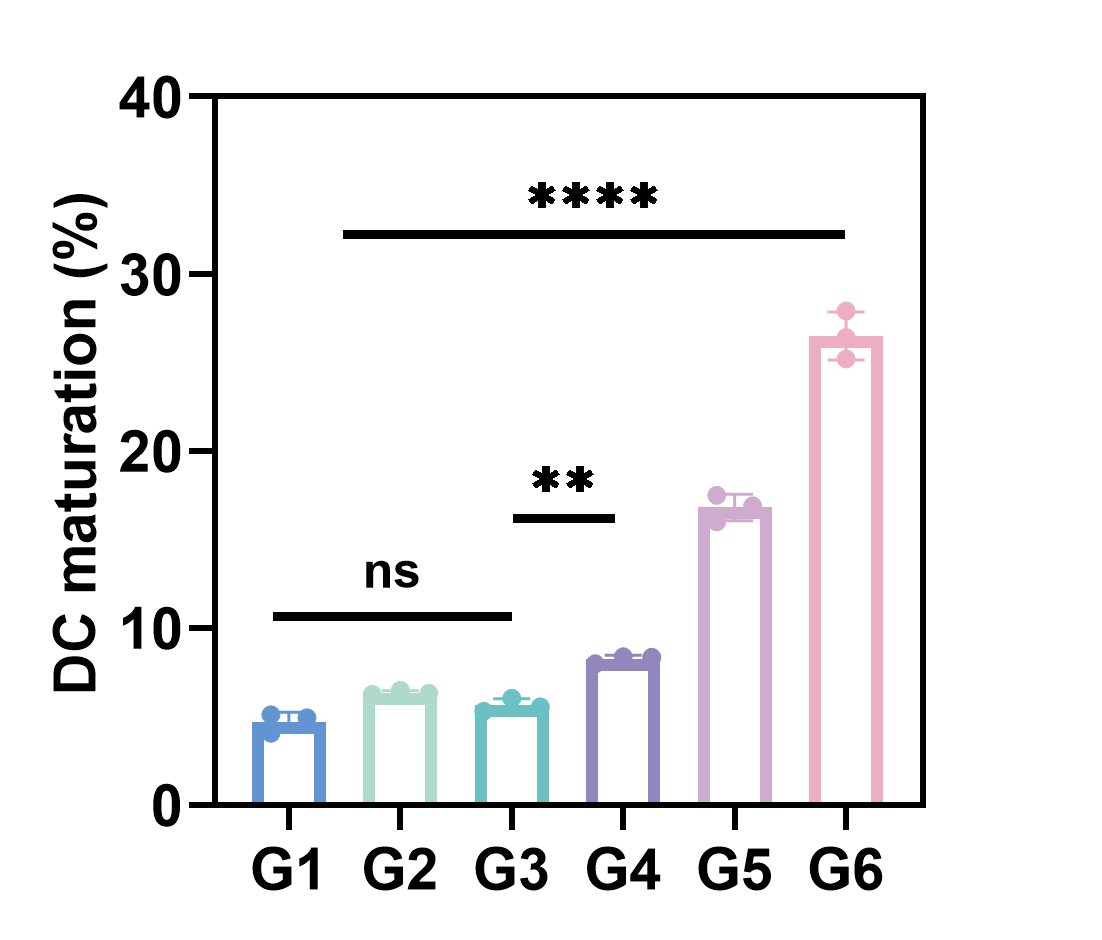


**Figure S46.** Statistical analysis of DC maturation in different treatment groups. Data are expressed as mean ± SD (n = 3). One-way Anova or t-test was used to analyze statistical differences between groups. *P < 0.05, **P < 0.01, ***P < 0.001, ****p < 0.0001. "ns" denotes no significant difference. Note, G1: Control, G2: CO, G3: CO&CHCA, G4: CZ@CC, G5: AuCZ@CC, G6: PEG@AuCZ@CC NPs.


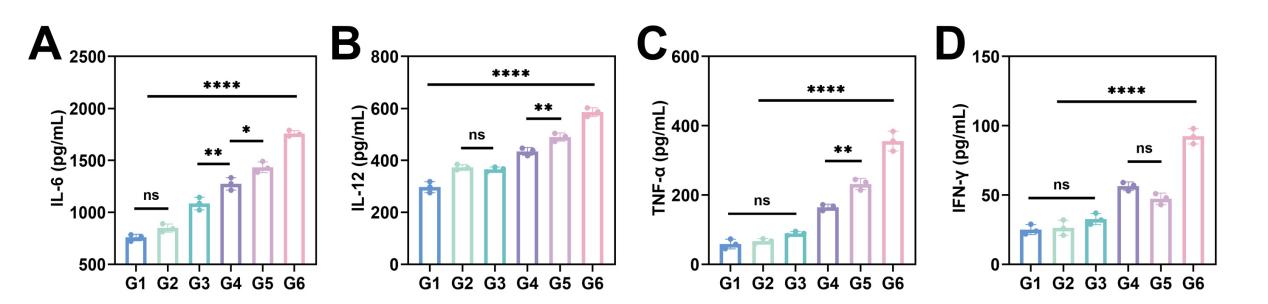


**Figure S47.** The secreted levels of cytokines, including **(A)** IL-6, **(B)** IL-12, **(C)** TNF-α, and **(D)** IFN-γ in different treatment groups. Data are expressed as mean ± SD (n = 3). One-way Anova or t-test was used to analyze statistical differences between groups. *P < 0.05, **P < 0.01, ***P < 0.001, ****p < 0.0001. "ns" denotes no significant difference. Note, G1: Control, G2: CO, G3: CO&CHCA, G4: CZ@CC, G5: AuCZ@CC, G6: PEG@AuCZ@CC NPs.


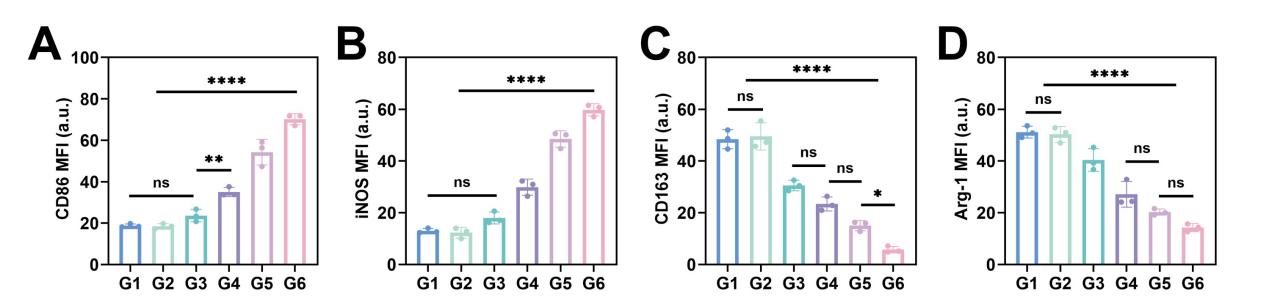


**Figure S48.** Quantitative data of Hepa1-6 cells stained with **(A)** anti-CD86, **(B)** anti-iNOS, **(C)** anti-CD163, and **(D)** anti-Arg-1 after different treatments. Data are expressed as mean ± SD (n = 3). One-way Anova or t-test was used to analyze statistical differences between groups. *P < 0.05, **P < 0.01, ***P < 0.001, ****p < 0.0001. "ns" denotes no significant difference. Note, G1: Control, G2: CO, G3: CO&CHCA, G4: CZ@CC, G5: AuCZ@CC, G6: PEG@AuCZ@CC NPs.


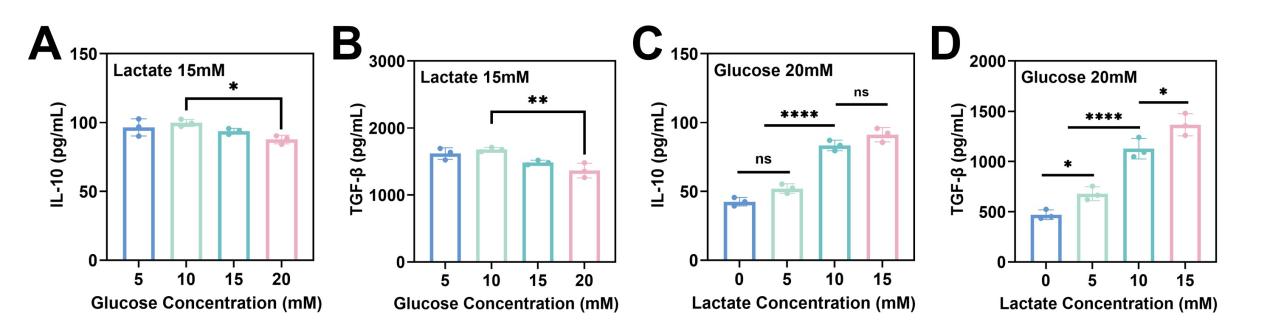


**Figure S49.** The secreted levels of IL-10 and TGF-β of Treg cells in different treatment groups. Data are expressed as mean ± SD (n = 3). One-way Anova or t-test was used to analyze statistical differences between groups. *P < 0.05, **P < 0.01, ***P < 0.001, ****p < 0.0001. "ns" denotes no significant difference. Note, G1: Control, G2: CO, G3: CO&CHCA, G4: CZ@CC, G5: AuCZ@CC, G6: PEG@AuCZ@CC NPs.


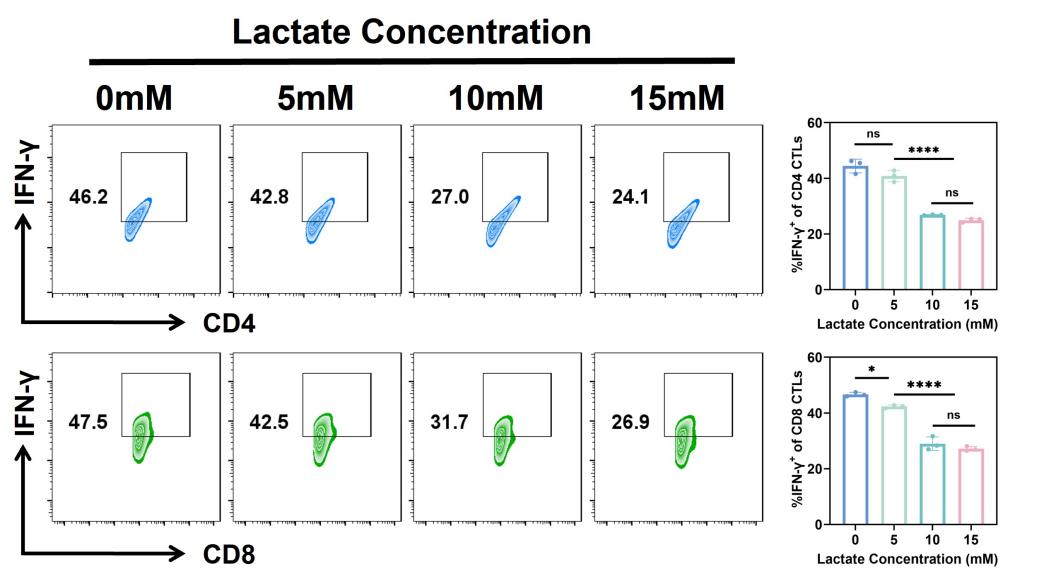


**Figure S50.** Flow cytometry analysis of CTLL-2 cells after various treatments. Data are expressed as mean ± SD (n = 3). One-way Anova or t-test was used to analyze statistical differences between groups. *P < 0.05, **P < 0.01, ***P < 0.001, ****p < 0.0001. "ns" denotes no significant difference. Note, G1: Control, G2: CO, G3: CO&CHCA, G4: CZ@CC, G5: AuCZ@CC, G6: PEG@AuCZ@CC NPs.


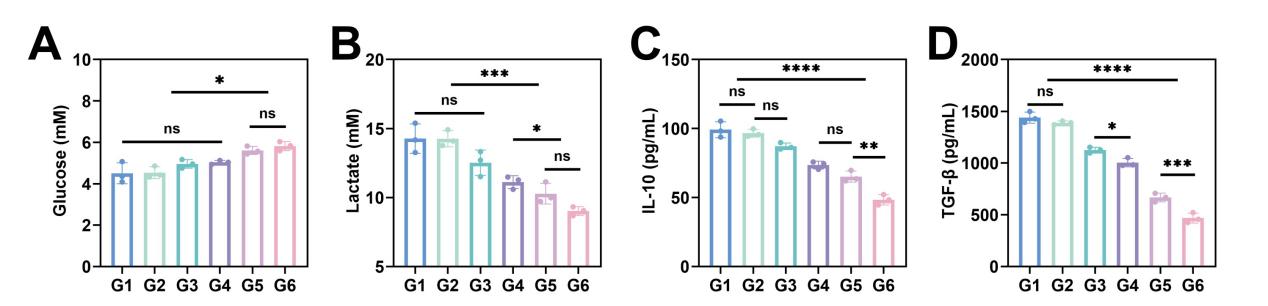


**Figure S51.** Analysis **(A)** glucose and **(B)** lactate concentrations of the collected supernatant. The secreted levels of **(C)** IL-10 and **(D)** TGF-β of Treg cells in different treatment groups. Data are expressed as mean ± SD (n = 3). One-way Anova or t-test was used to analyze statistical differences between groups. *P < 0.05, **P < 0.01, ***P < 0.001, ****p < 0.0001. "ns" denotes no significant difference. Note, G1: Control, G2: CO, G3: CO&CHCA, G4: CZ@CC, G5: AuCZ@CC, G6: PEG@AuCZ@CC NPs.


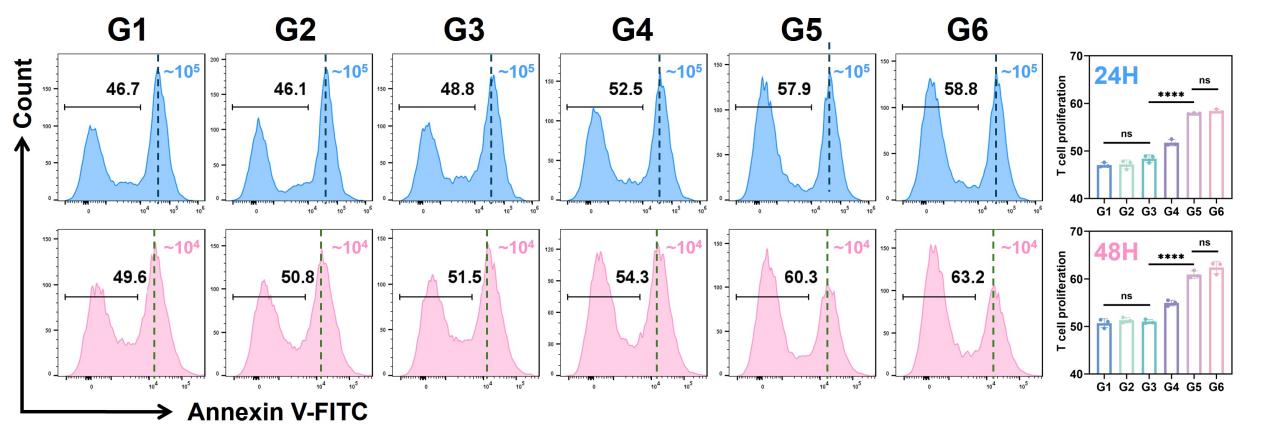


**Figure S52.** CFSE-based flow cytometry analysis of CTLL-2 cells after various treatments and different time (24 and 48 hours). Data are expressed as mean ± SD (n = 3). One-way Anova or t-test was used to analyze statistical differences between groups. *P < 0.05, **P < 0.01, ***P < 0.001, ****p < 0.0001. "ns" denotes no significant difference. Note, G1: Control, G2: CO, G3: CO&CHCA, G4: CZ@CC, G5: AuCZ@CC, G6: PEG@AuCZ@CC NPs.


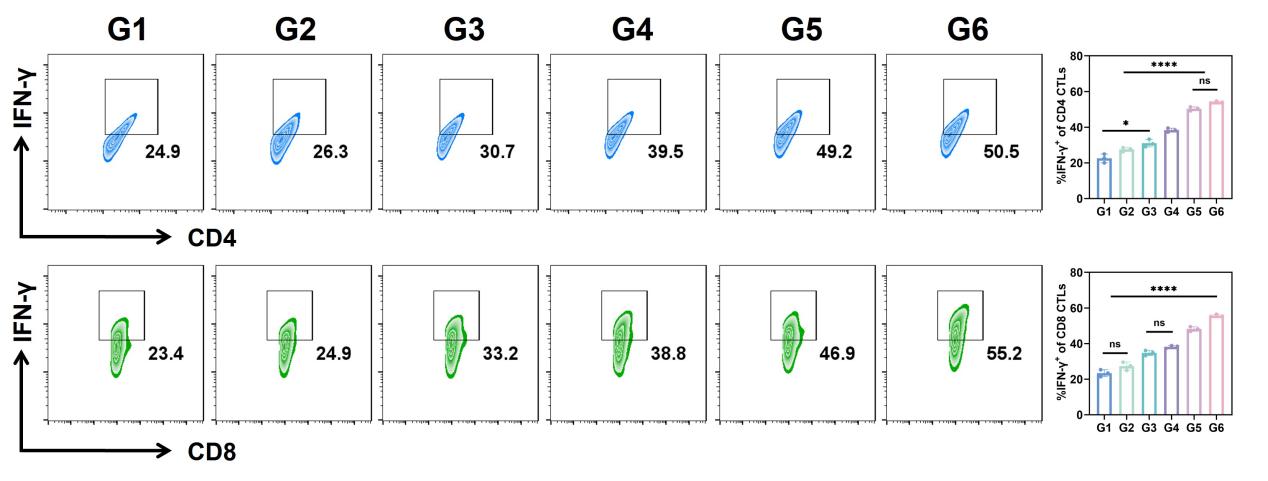


**Figure S53.** Flow cytometry analysis of CTLL-2 cells after various treatments. Data are expressed as mean ± SD (n = 3). One-way Anova or t-test was used to analyze statistical differences between groups. *P < 0.05, **P < 0.01, ***P < 0.001, ****p < 0.0001. "ns" denotes no significant difference. Note, G1: Control, G2: CO, G3: CO&CHCA, G4: CZ@CC, G5: AuCZ@CC, G6: PEG@AuCZ@CC NPs.


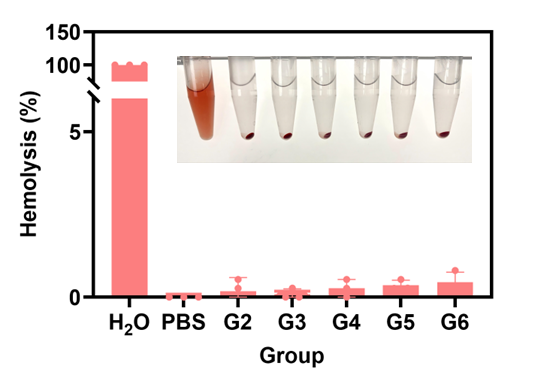


**Figure S54.** Hemolysis rates and digital photos of red blood cells after incubation with water and PBS solution with different nanomedicines (33 µg/mL). Data are expressed as mean ± SD (n = 3). Note, G2: CO, G3: CO&CHCA, G4: CZ@CC, G5: AuCZ@CC, G6: PEG@AuCZ@CC NPs.


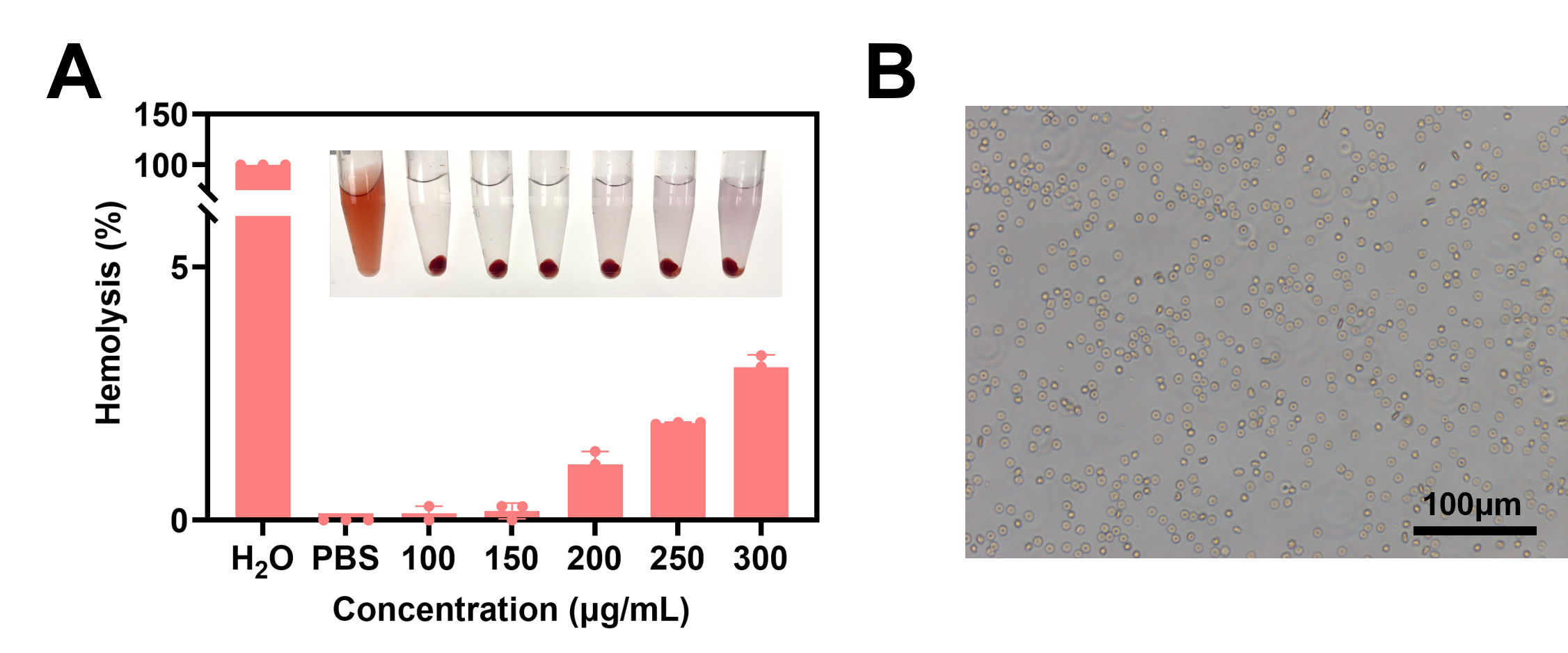


**Figure S55.** Hemolysis rates and digital photos of red blood cells after incubation with water and PEG@AuCZ@CC NPs in PBS solution with varied concentrations. (Scale bar: 100 µm). Data are expressed as mean ± SD (n = 3).


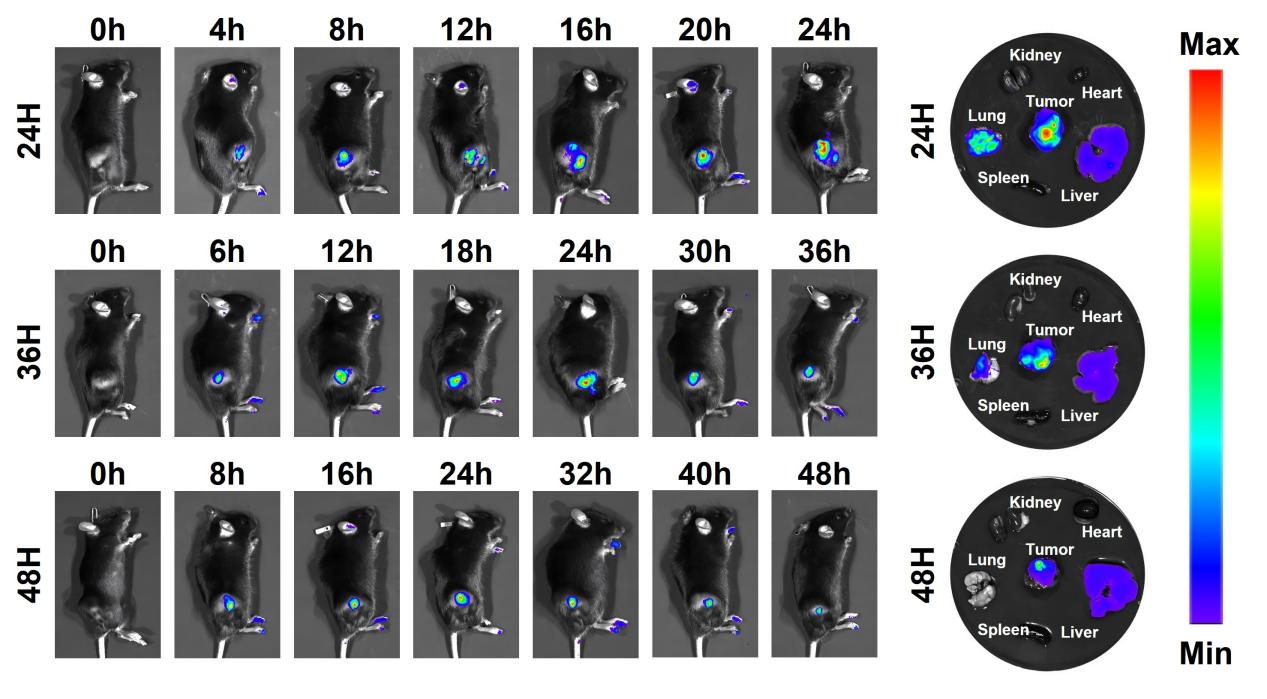


**Figure S56. (A)** Time-dependent fluorescence images in vivo of mice bearing Hepa1-6 tumor after injecting Cy5.5-labeled PEG@AuCZ@CC NPs. **(B)** ex vivo fluorescence images of collected tumor and normal organs after 24 h, 36h, and 48h post-injection.


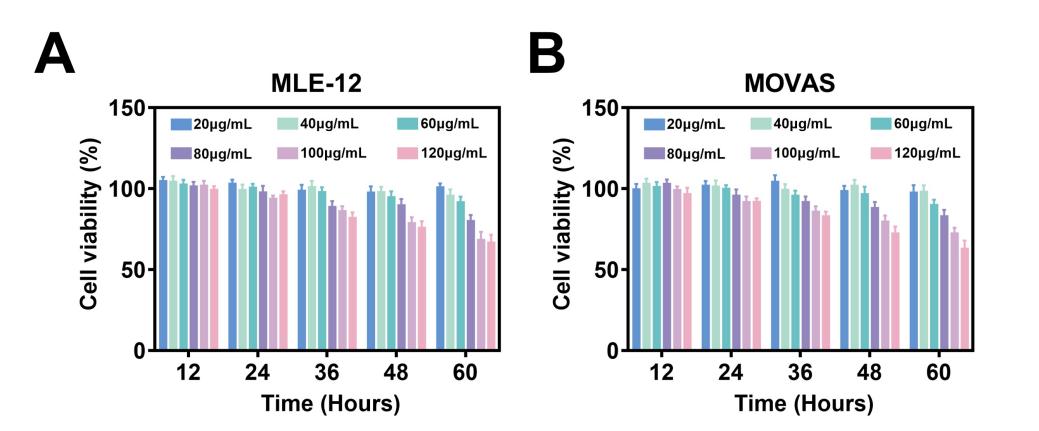


**Figure S57.** Cytotoxicity assay of **(A)** MLE-12 cells and **(B)** MOVAS cells after PEG@AuCZ@CC treatment for different time-points (12h, 24h, 36h, 48h, and 60h), n = 6.


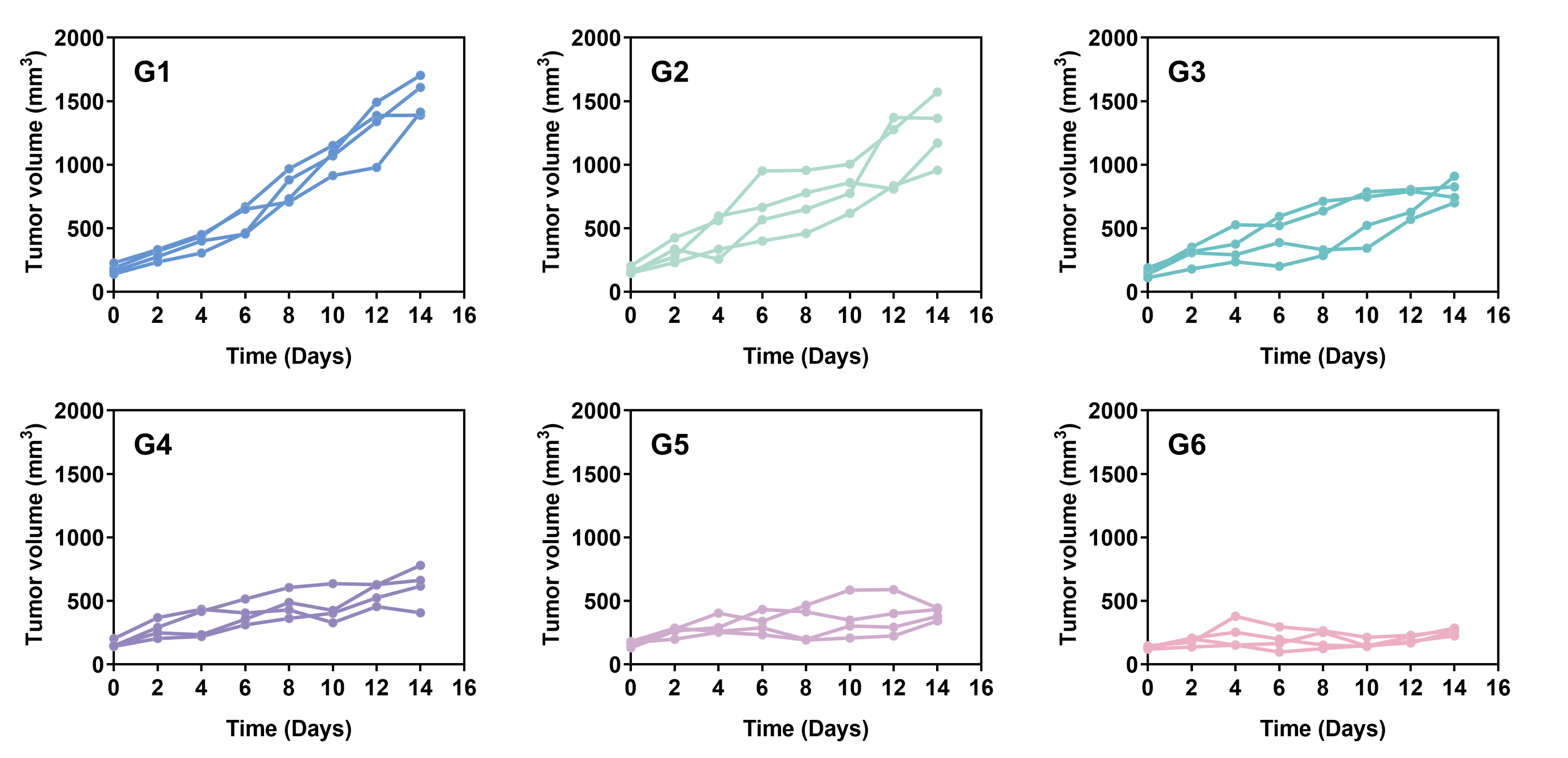


**Figure S58.** Tumor growth profile in each mouse as a function of time in G1-G6 that experienced corresponding treatments. G1: Control, G2: CO, G3: CO&CHCA, G4: CZ@CC, G5: AuCZ@CC, G6: PEG@AuCZ@CC.


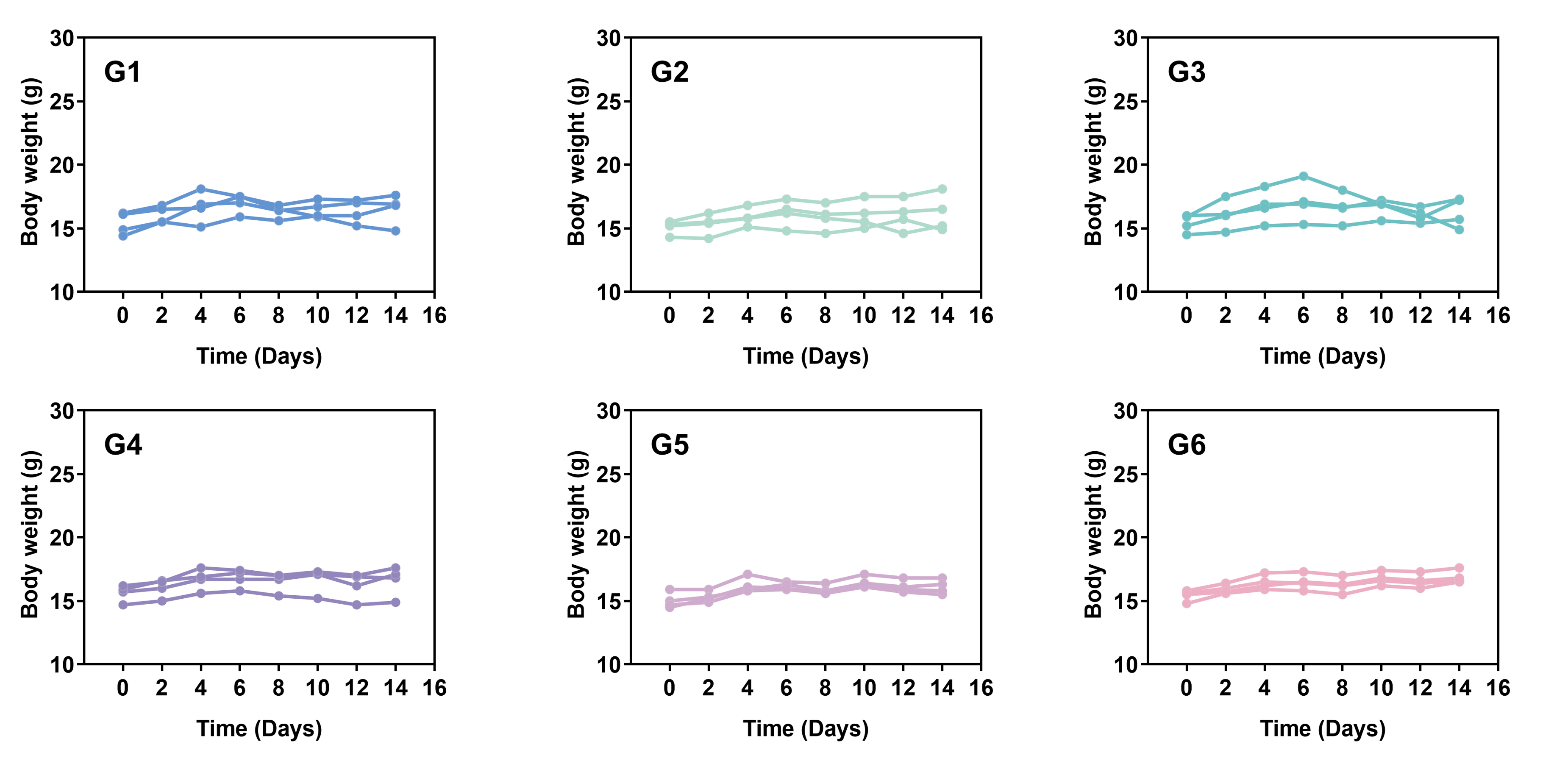


**Figure S59.** Time-dependent body weight of each mouse in G1-G6. G1: Control, G2: CO, G3: CO&CHCA, G4: CZ@CC, G5: AuCZ@CC, G6: PEG@AuCZ@CC.


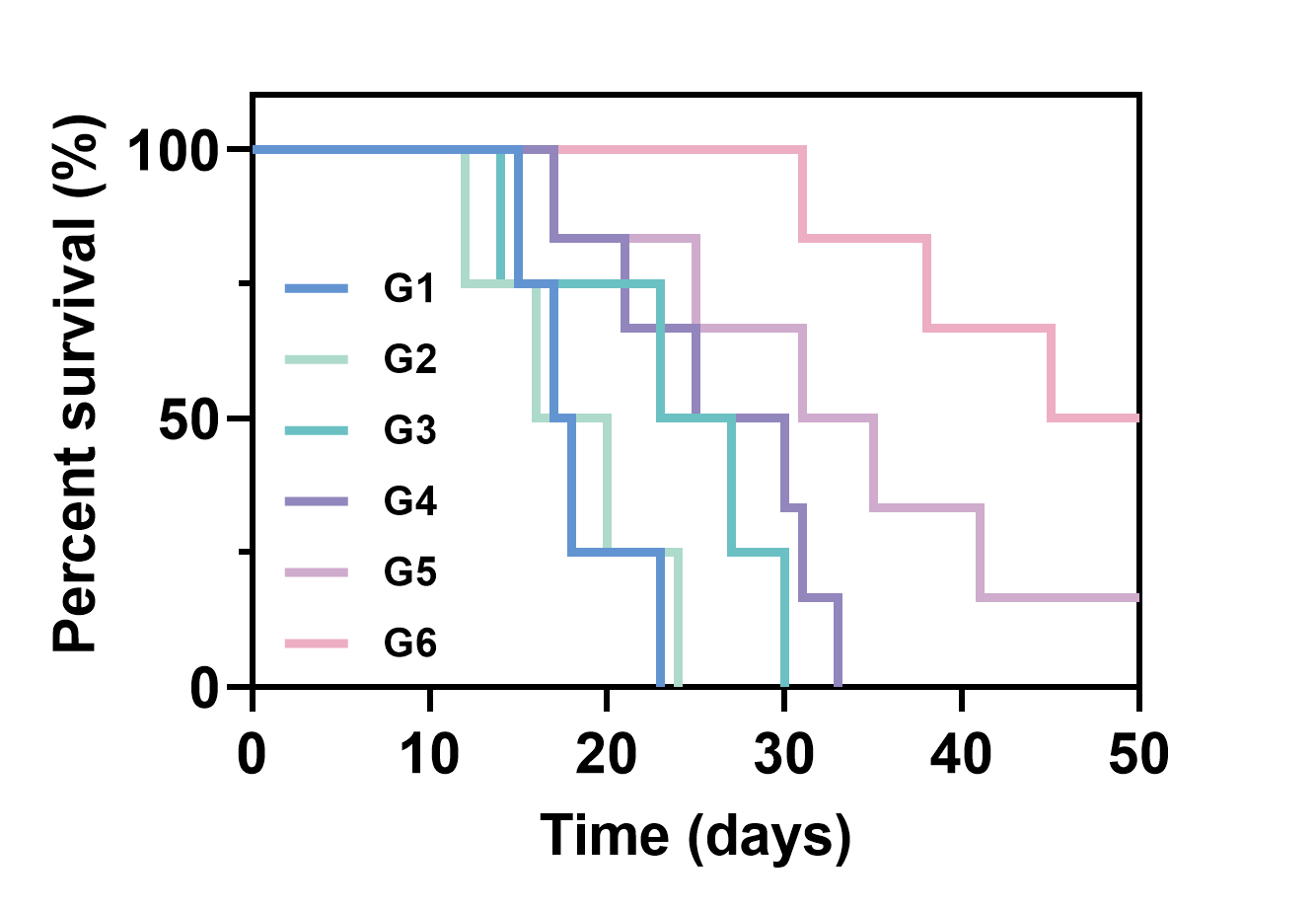


**Figure S60.** Time-dependent survival rate of mice in G1-G6. G1: Control, G2: CO, G3: CO&CHCA, G4: CZ@CC, G5: AuCZ@CC, G6: PEG@AuCZ@CC.


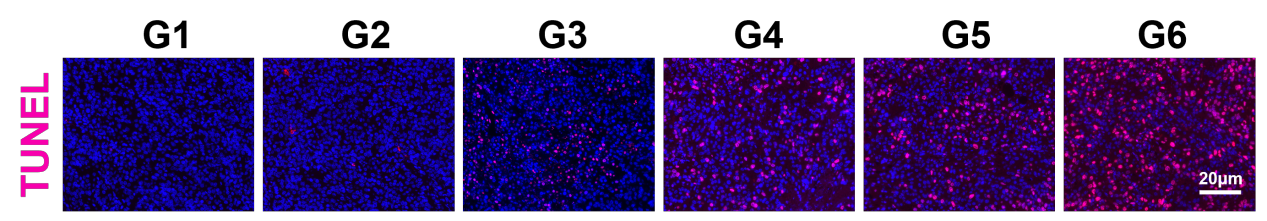


**Figure S61.** TUNEL immunofluorescence images of collected tumor slices in G1-G6 on Day 14. (Scale bar: 20 µm). G1: Control, G2: CO, G3: CO&CHCA, G4: CZ@CC, G5: AuCZ@CC, G6: PEG@AuCZ@CC.


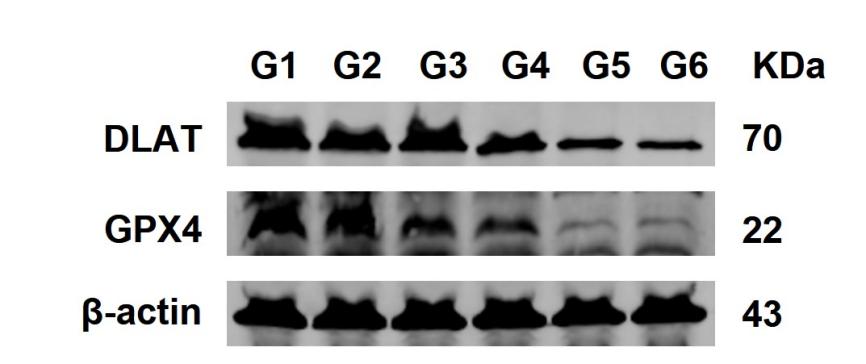


**Figure S62.** WB bands of main proteins (DLAT and GPX4) in collected tumor tissues from G1-G6.


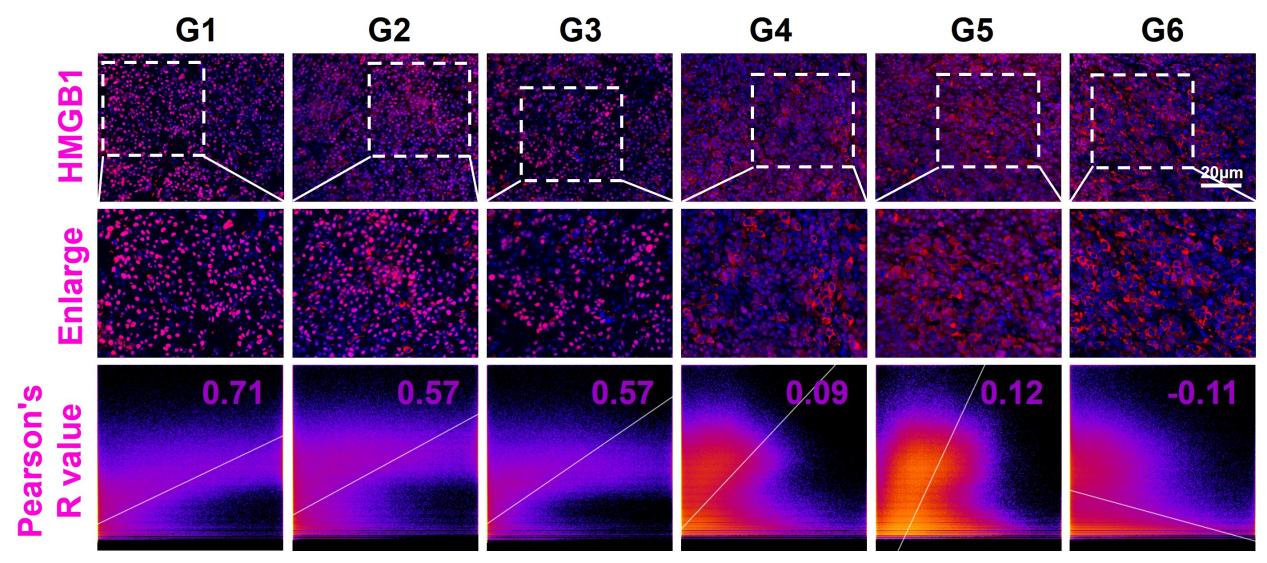


**Figure S63.** Immunofluorescence images and co-localization analysis of HMGB1 in tumor tissues after various treatments. (Scale bar: 20 µm).


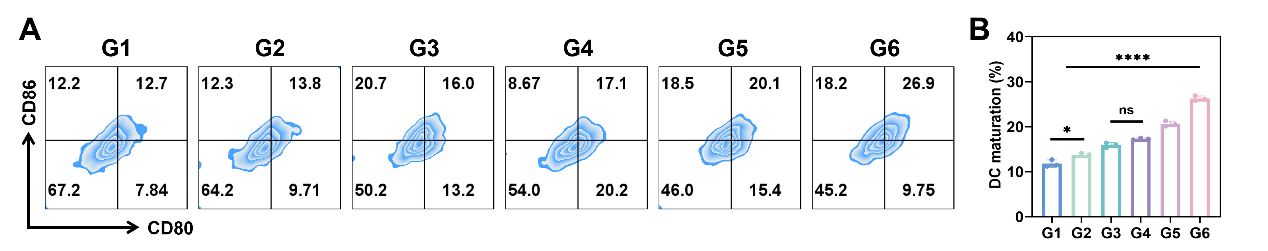


**Figure S64.** (A) Flow cytometry analysis and (B) histograms of dendritic cells (DCs) in the tumor of mice receiving various treatments. Data are expressed as mean ± SD (n = 3). One-way Anova or t-test was used to analyze statistical differences between groups. *P < 0.05, **P < 0.01, ***P < 0.001, ****p < 0.0001. "ns" denotes no significant difference. Note, G1: Control, G2: CO, G3: CO&CHCA, G4: CZ@CC, G5: AuCZ@CC, G6: PEG@AuCZ@CC NPs.


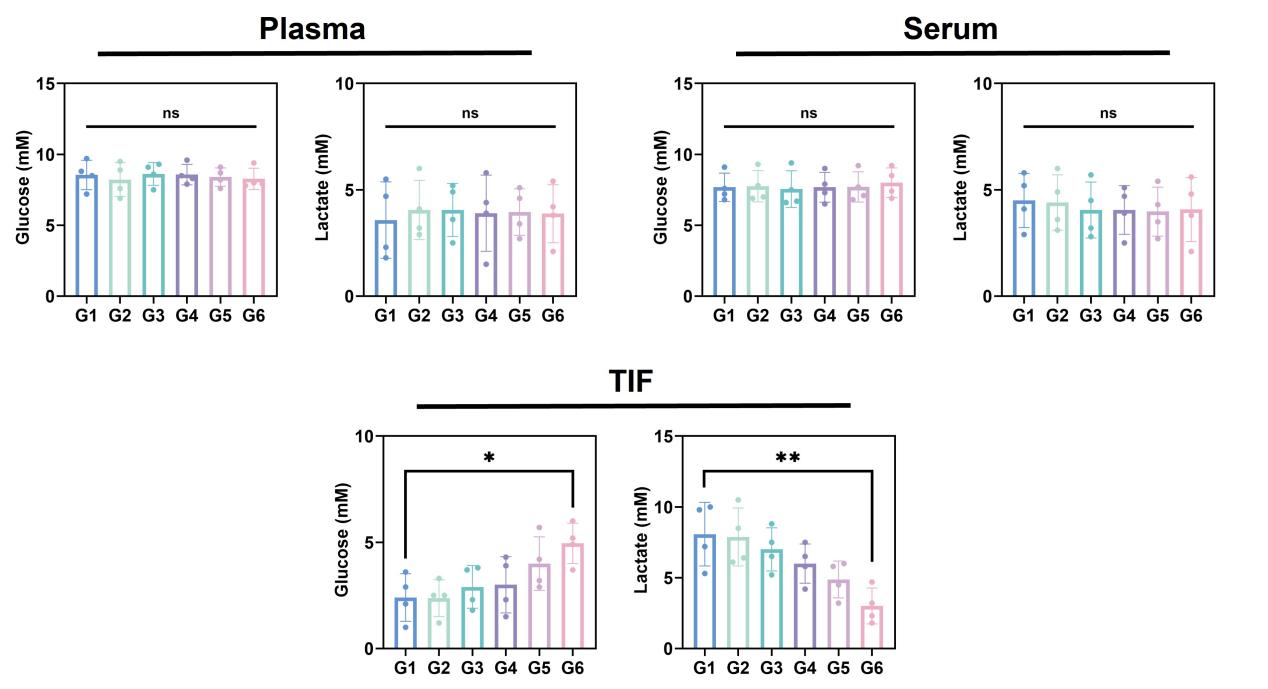


**Figure S65.** Glucose and lactate levels in serum, plasma, and tumor interstitial fluid after various treatments. Data are expressed as mean ± SD (n = 4). One-way Anova or t-test was used to analyze statistical differences between groups. *P < 0.05, **P < 0.01, ***P < 0.001, ****p < 0.0001. "ns" denotes no significant difference. Note, G1: Control, G2: CO, G3: CO&CHCA, G4: CZ@CC, G5: AuCZ@CC, G6: PEG@AuCZ@CC NPs.


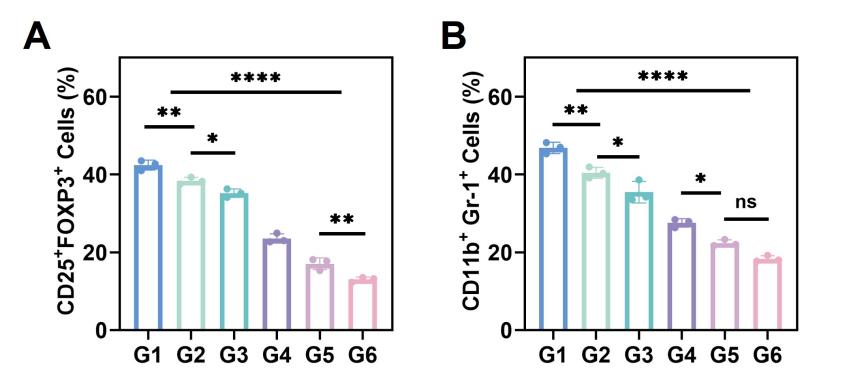


**Figure S66.** Statistical analysis of **(A)** Treg cells, and **(B)** MDSCs in Hepa1-6 tumor receiving various treatments. Data are expressed as mean ± SD (n = 3). One-way Anova or t-test was used to analyze statistical differences between groups. *P < 0.05, **P < 0.01, ***P < 0.001, ****p < 0.0001. "ns" denotes no significant difference. Note, G1: Control, G2: CO, G3: CO&CHCA, G4: CZ@CC, G5: AuCZ@CC, G6: PEG@AuCZ@CC NPs.


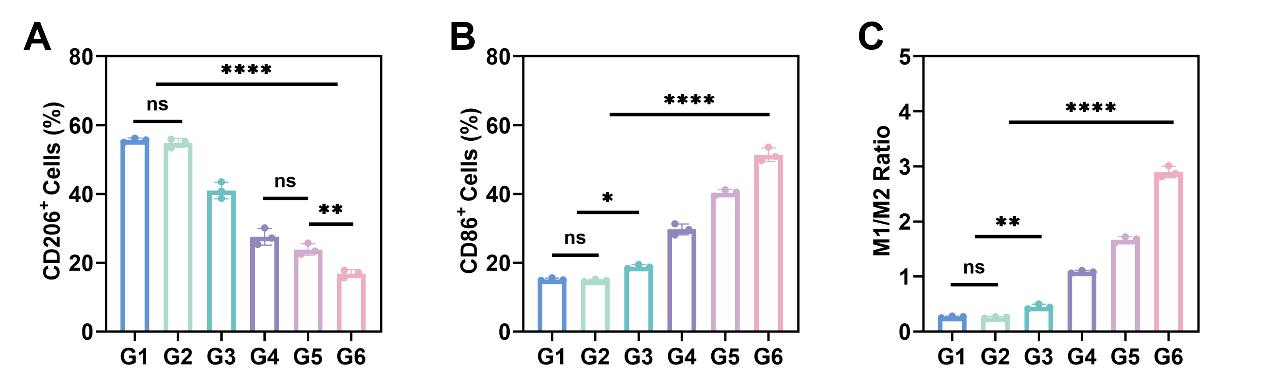


**Figure S67.** Percentage of **(A)** M2-TAMs (CD206+), **(B)** M1-TAMs (CD86+) and **(C)** the ratios of M1/M2 macrophages in tumors of mice after various treatments. Data are expressed as mean ± SD (n = 3). One-way Anova or t-test was used to analyze statistical differences between groups. *P < 0.05, **P < 0.01, ***P < 0.001, ****p < 0.0001. "ns" denotes no significant difference. Note, G1: Control, G2: CO, G3: CO&CHCA, G4: CZ@CC, G5: AuCZ@CC, G6: PEG@AuCZ@CC NPs.


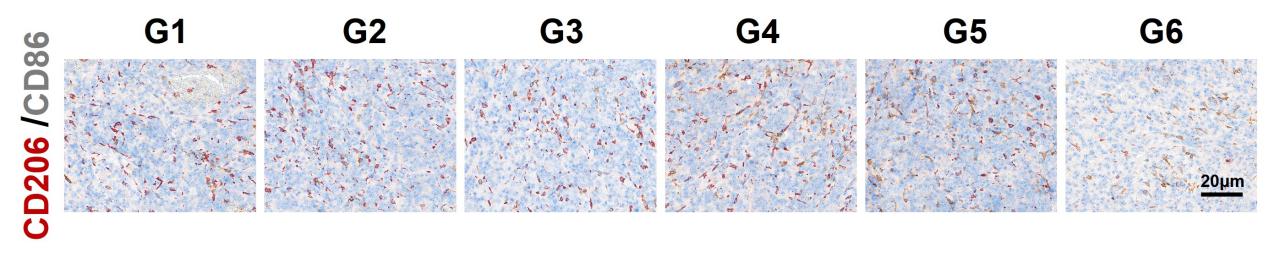


**Figure S68.** Immunohistochemistry images of CD206 (red) and CD86 (yellow) in tumor tissues after various treatments. (Scale bar: 20 µm).


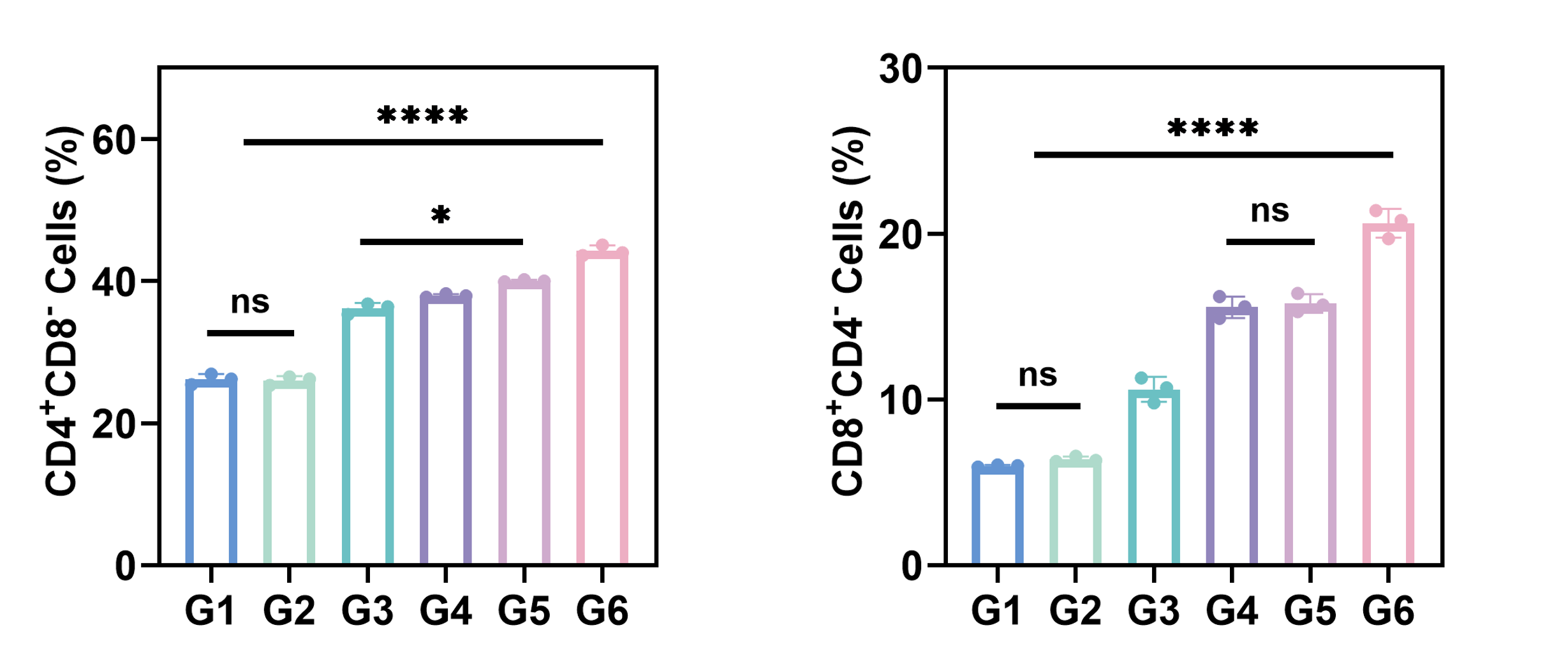


**Figure S69.** Statistical analysis of CD4^+^ T cells and CD8^+^ T cells in Hepa1-6 tumor receiving various treatments. Data are expressed as mean ± SD (n = 3). One-way Anova or t-test was used to analyze statistical differences between groups. *P < 0.05, **P < 0.01, ***P < 0.001, ****p < 0.0001. "ns" denotes no significant difference. Note, G1: Control, G2: CO, G3: CO&CHCA, G4: CZ@CC, G5: AuCZ@CC, G6: PEG@AuCZ@CC NPs.


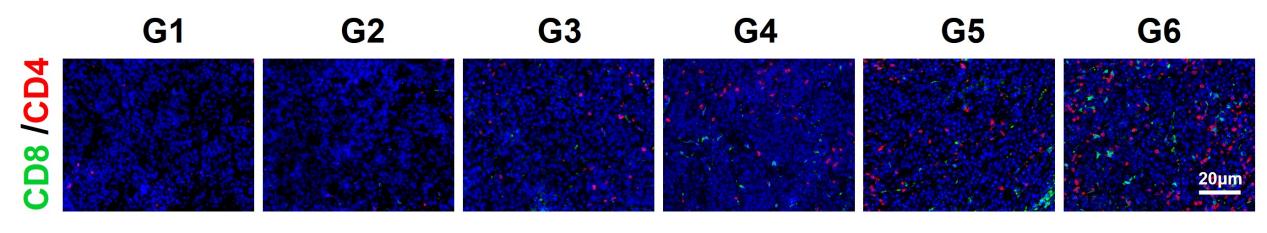


**Figure S70.** Immunofluorescence images of CD4^+^/CD8^+^ T cells in tumor tissues after various treatments. (Scale bar: 20 µm).


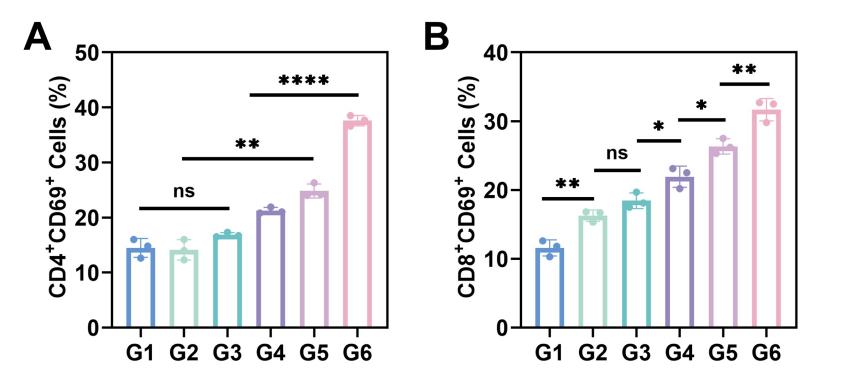


**Figure S71.** Statistical analysis of **(A)** CD4^+^CD69^+^ T cells and **(B)** CD8^+^CD69^+^ T cells in Hepa1-6 tumor receiving various treatments. Data are expressed as mean ± SD (n = 3). One-way Anova or t-test was used to analyze statistical differences between groups. *P < 0.05, **P < 0.01, ***P < 0.001, ****p < 0.0001. "ns" denotes no significant difference. Note, G1: Control, G2: CO, G3: CO&CHCA, G4: CZ@CC, G5: AuCZ@CC, G6: PEG@AuCZ@CC NPs.


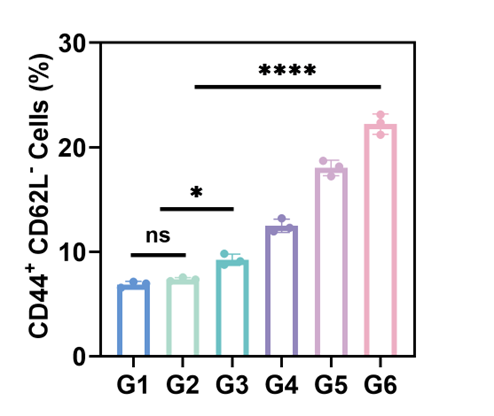


**Figure S72.** Statistical analysis of effector memory T cells in spleen receiving various treatments. Data are expressed as mean ± SD (n = 3). One-way Anova or t-test was used to analyze statistical differences between groups. *P < 0.05, **P < 0.01, ***P < 0.001, ****p < 0.0001. "ns" denotes no significant difference. Note, G1: Control, G2: CO, G3: CO&CHCA, G4: CZ@CC, G5: AuCZ@CC, G6: PEG@AuCZ@CC NPs.


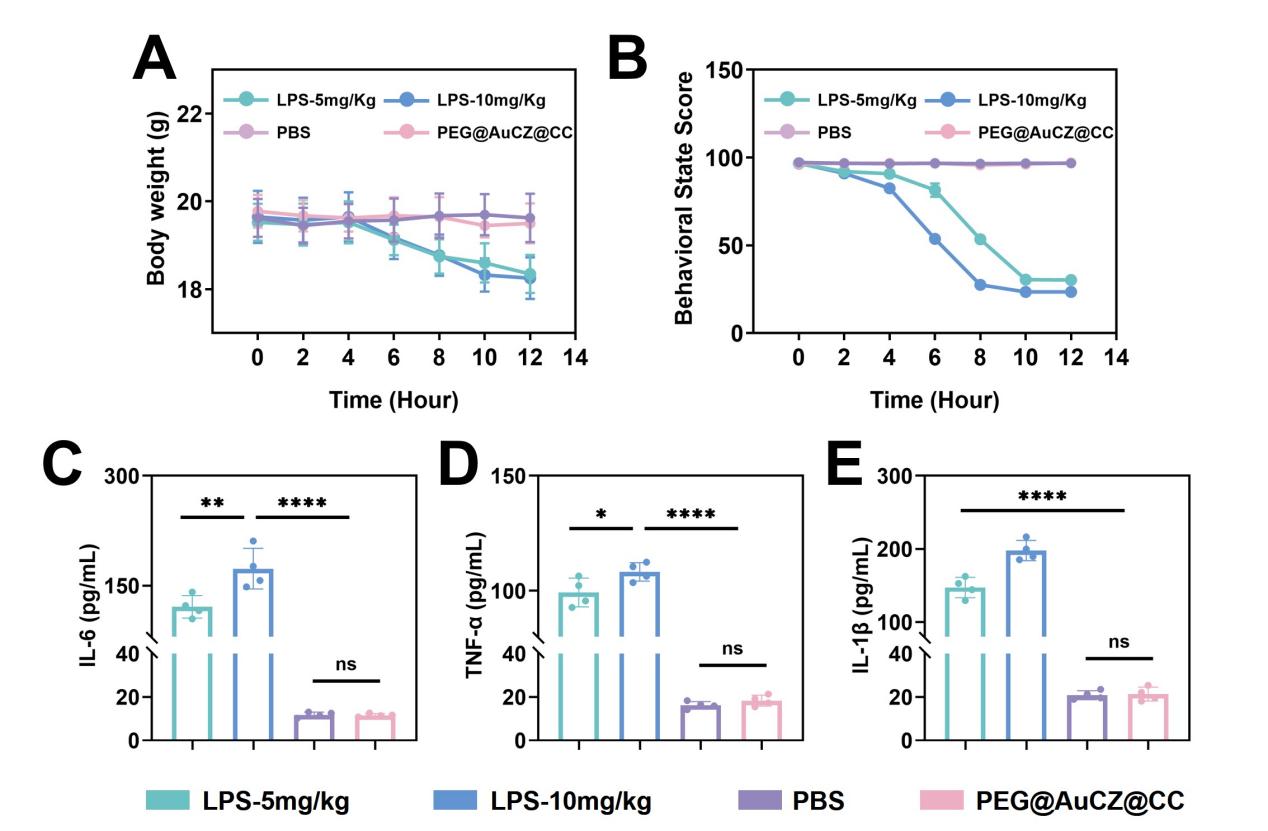


**Figure S73. (A)** Changes in body weight across differen groups. **(B)** Changes in behavioral status scores across different groups. The levels of **(C)** IL-6, **(D)** TNF-α, and **(E)** IL-1β in mouse serum across different groups. Data are expressed as mean ± SD (n = 4). One-way Anova or t-test was used to analyze statistical differences between groups. *P < 0.05, **P < 0.01, ***P < 0.001, ****p < 0.0001. "ns" denotes no significant difference.


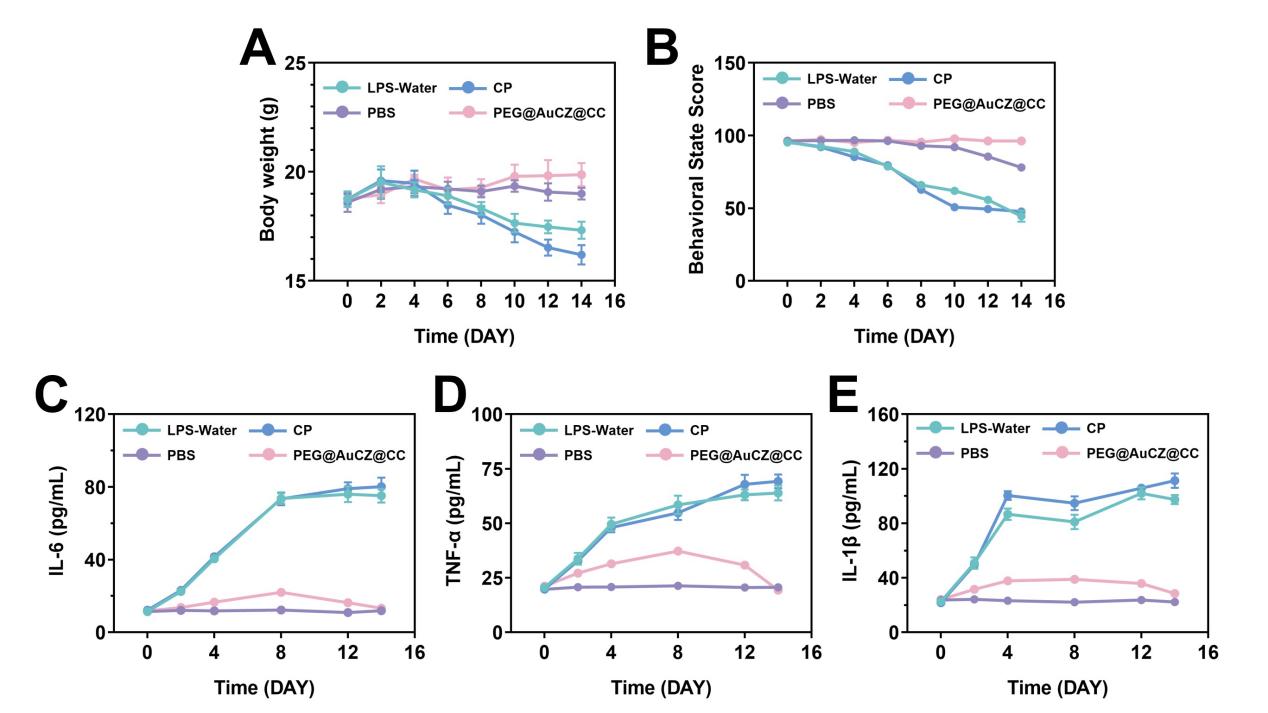


**Figure S74. (A)** Changes in body weight across differen groups. **(B)** Changes in behavioral status scores across different groups. The levels of **(C)** IL-6, **(D)** TNF-α, and **(E)** IL-1β in mouse serum across different groups and different time-points. Data are expressed as mean ± SD (n = 4). One-way Anova or t-test was used to analyze statistical differences between groups. *P < 0.05, **P < 0.01, ***P < 0.001, ****p < 0.0001. "ns" denotes no significant difference.


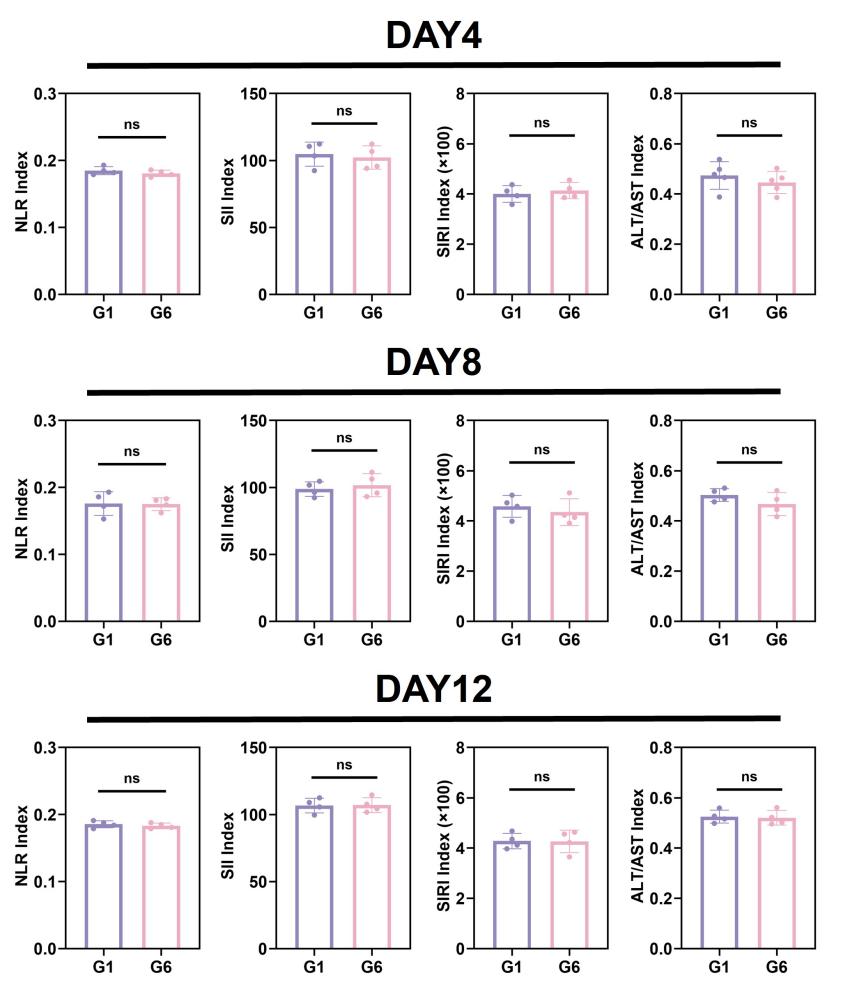


**Figure S75.** At three time points (days 4, 8, and 12), changes in NLR, SII, SIRI, and ALT/AST ratios between G1and G6. Data are expressed as mean ± SD. One-way Anova or t-test was used to analyze statistical differences between groups. *P < 0.05, **P < 0.01, ***P < 0.001, ****p < 0.0001. "ns" denotes no significant difference. Note, G1: PBS, G6: PEG@AuCZ@CC NPs.


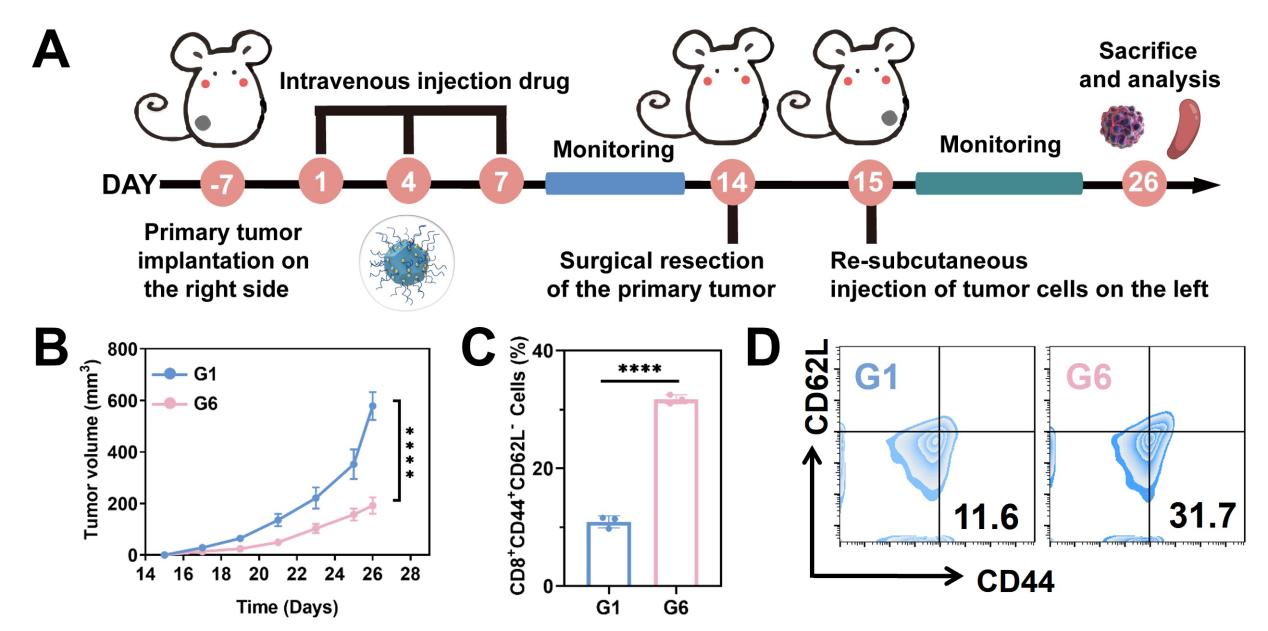


**Figure S76. (A)** Flowchart of the Tumor rechallenge Model. **(B)** Tumor volumes in each group as a function of time. **(C) (D)** Flow cytometry and statistical analysis of memory T cells in the spleen of mice receiving various treatments. Data are expressed as mean ± SD (n = 3). One-way Anova or t-test was used to analyze statistical differences between groups. *P < 0.05, **P < 0.01, ***P < 0.001, ****p < 0.0001. "ns" denotes no significant difference. Note, G1: Control, G2: CO, G3: CO&CHCA, G4: CZ@CC, G5: AuCZ@CC, G6: PEG@AuCZ@CC NPs.


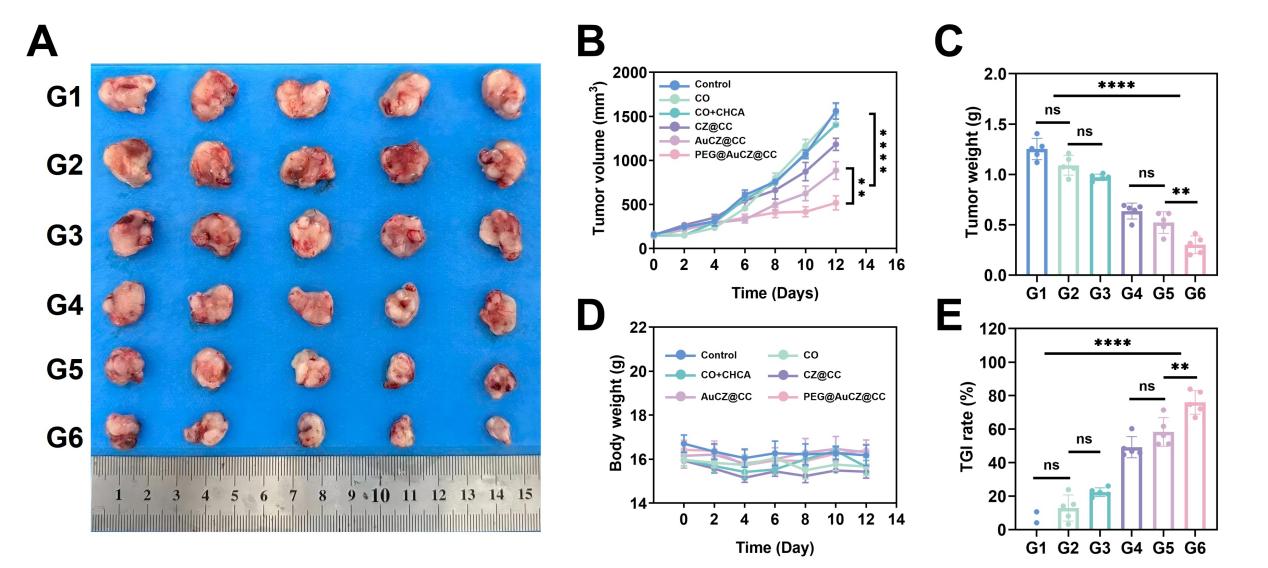


**Figure S77. (A)** Digital photograph of tumors after diverse treatments. **(B)** Tumor volumes in each group as a function of time in G1-G6 that experienced corresponding treatments. **(C)** Tumor weights collected on Day 12 in G1-G6. **(D)** Time-dependent body weight of mice in G1-G6. **(E)** TGI rates in each group. Data are expressed as mean ± SD (n = 5). One-way Anova or t-test was used to analyze statistical differences between groups. *P < 0.05, **P < 0.01, ***P < 0.001, ****p < 0.0001. "ns" denotes no significant difference. Note, G1: Control, G2: CO, G3: CO&CHCA, G4: CZ@CC, G5: AuCZ@CC, G6: PEG@AuCZ@CC NPs.


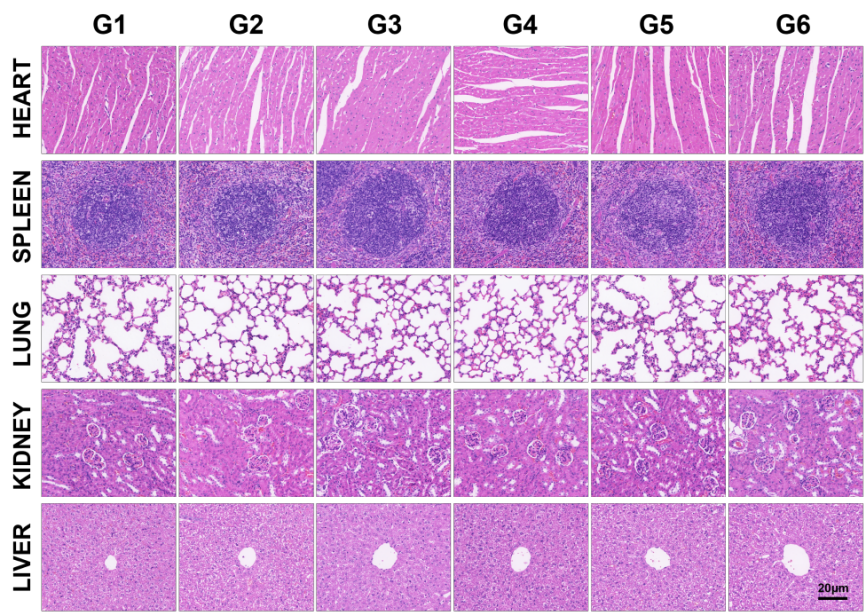


**Figure S78.** Optical photos of normal organ slices after different treatments. (Scale bar: 20 µm). G1: Control, G2: CO, G3: CO&CHCA, G4: CZ@CC, G5: AuCZ@CC, G6: PEG@AuCZ@CC NPs.


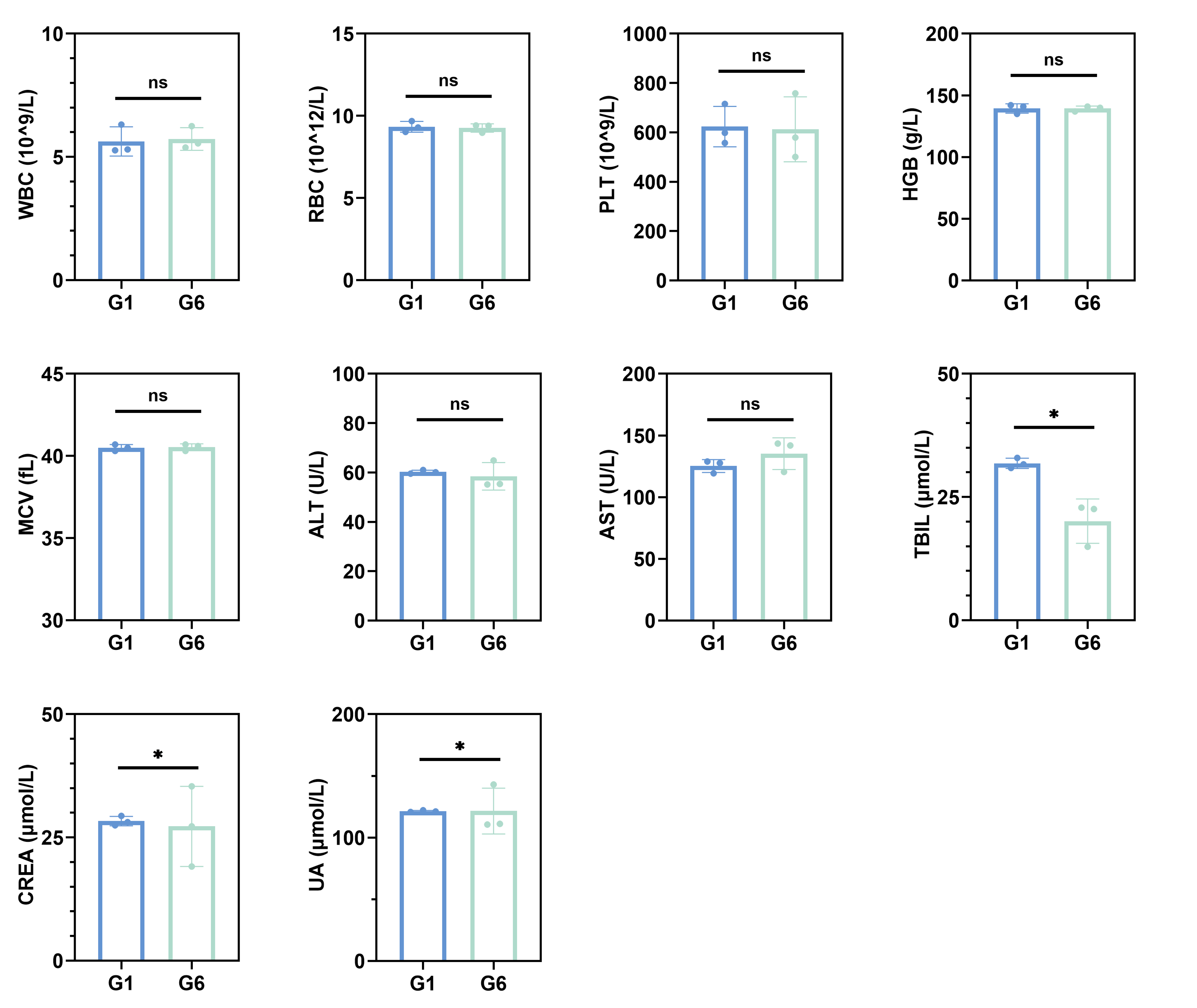


**Figure S79.** Serum levels of ALT, AST, TBIL, CREA, and UA in mice treated with PEG@AuCZ@CC NPs. Routine blood tests were performed to assess these parameters. Data are expressed as mean ± SD (n = 3). Statistical comparisons between groups were conducted using a t-test. *P < 0.05, **P < 0.01, ***P < 0.001, ****p < 0.0001. "ns" denotes no significant difference. Note, G1: Control, G6: PEG@AuCZ@CC NPs.


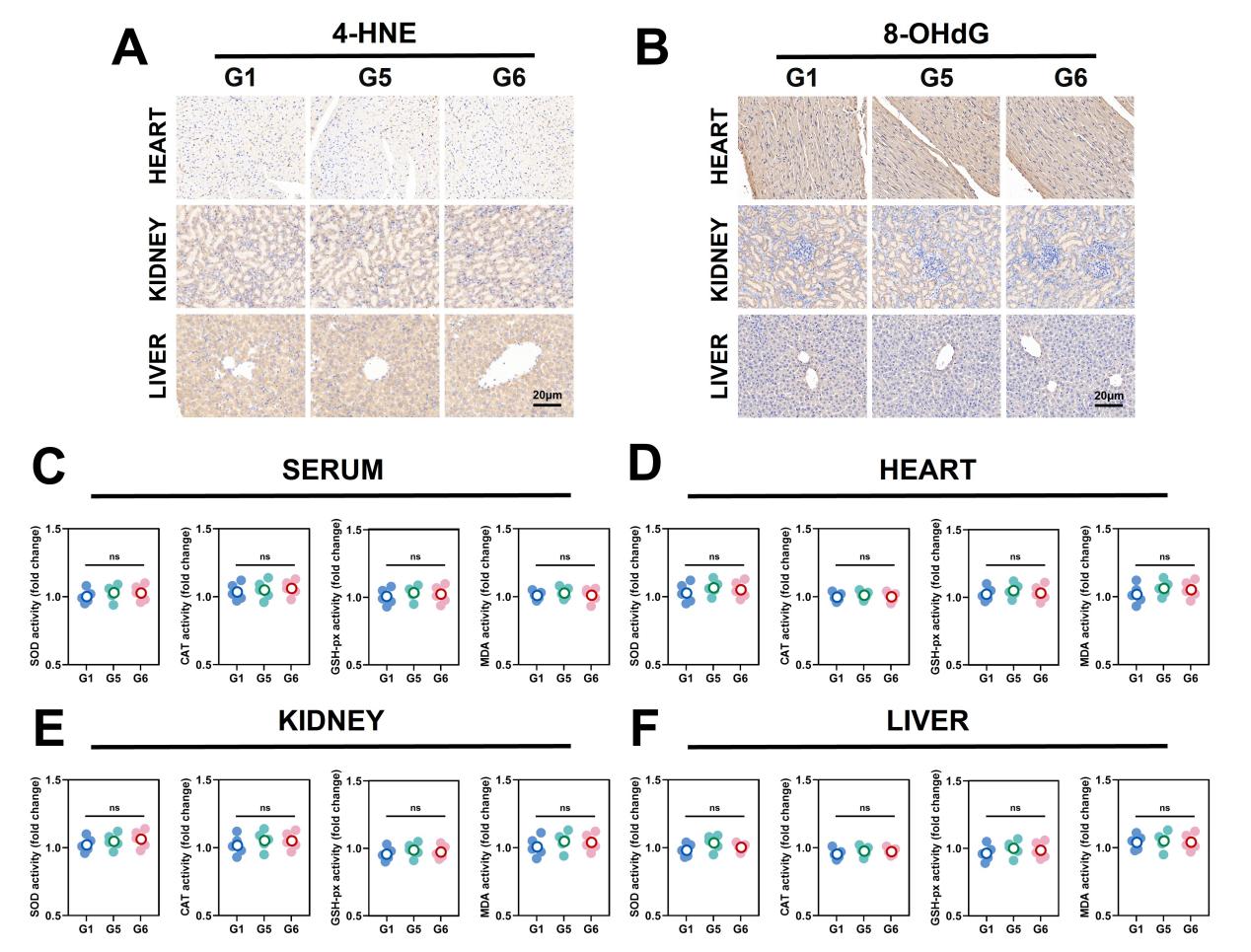


**Figure S80. (A-B)** Histological marker detection (4-HNE and 8-OHdG staining) in the liver, kidney, and heart tissues. **(C-F)** Functional enzyme activity assays (SOD, CAT, GSH-Px, and MDA) in the serum, liver, kidney, and heart. Data are expressed as mean ± SD. Statistical comparisons between groups were conducted using a t-test. *P < 0.05, **P < 0.01, ***P < 0.001, ****p < 0.0001. "ns" denotes no significant difference. Note, G1: Control, G5: AuCZ@CC, and G6: PEG@AuCZ@CC NPs.


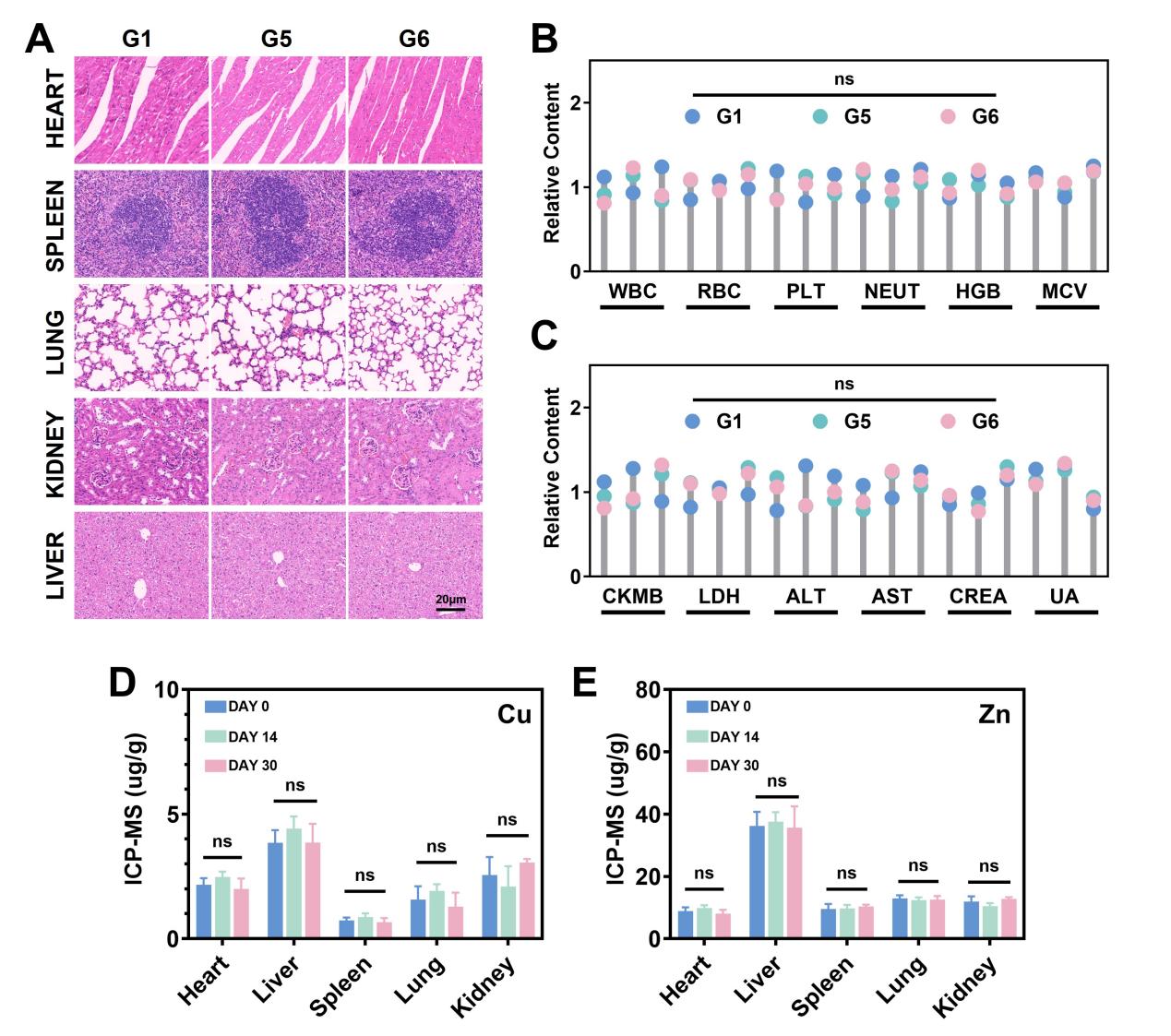


**Figure S81. (A)** Optical photos of normal organ slices after different treatments. (Scale bar: 20 µm). **(B-C)** Blood was collected from the mice for analysis (WBC, RBC, Hb, PLT, NEUT, HGB, MCV, CK-MB, LDH, ALT, AST, CREA, and UA). Quantitative levels of **(D)** Cu and **(E)** Zn in different organs at day 0/14/30 via the inductively coupled plasma mass spectrometry (ICP-MS) technology. Data are expressed as mean ± SD. Statistical comparisons between groups were conducted using a t-test. *P < 0.05, **P < 0.01, ***P < 0.001, ****p < 0.0001. "ns" denotes no significant difference. Note, G1: Control, G5: AuCZ@CC, and G6: PEG@AuCZ@CC NPs.
